# Mutagenic Analysis of a DNA Translocating Tube’s Interior Surface

**DOI:** 10.3390/v12060670

**Published:** 2020-06-22

**Authors:** Aaron P. Roznowski, Julia M. Fisher, Bentley A. Fane

**Affiliations:** 1 The BIO5 Institute, University of Arizona, Tucson, AZ 85721, USA; aroznowski@email.arizona.edu; 2 Statistics Consulting Lab, The BIO5 Institute, University of Arizona, Tucson, AZ 85721, USA; julia@statlab.bio5.org

**Keywords:** ϕX174, microviridae, penetration, DNA pilot protein

## Abstract

Bacteriophage ϕX174 uses a decamer of DNA piloting proteins to penetrate its host. These proteins oligomerize into a cell wall-spanning tube, wide enough for genome passage. While the inner surface of the tube is primarily lined with inward-facing amino acid side chains containing amide and guanidinium groups, there is a 28 Å-long section near the tube’s C-terminus that does not exhibit this motif. The majority of the inward-facing residues in this region are conserved across the three ϕX174-like clades, suggesting that they play an important role during genome delivery. To test this hypothesis, and explore the general function of the tube’s inner surface, non-glutamine residues within this region were mutated to glutamine, while existing glutamine residues were changed to serine. Four of the resulting mutants had temperature-dependent phenotypes. Virion assembly, host attachment, and virion eclipse, defined as the cell’s ability to inactivate the virus, were not affected. Genome delivery, however, was inhibited. The results support a model in which a balance of forces governs genome delivery: potential energy provided by the densely packaged viral genome and/or an osmotic gradient move the genome into the cell, while the tube’s inward facing glutamine residues exert a frictional force, or drag, that controls genome release.

## 1. Introduction

To establish an infection, bacteriophages must move hydrophilic genomes through hydrophobic host cell membranes. After binding a host surface receptor, virions typically undergo irreversible conformational changes that often allow specialized proteins to create channels through the host cell wall. Genome movement through this channel may be driven by potential energy stored within the highly condensed, packaged genome, or by the osmotic gradient existing between the host cytoplasm and the external environment [1,2]. 

Prokaryotic viruses use a diverse array of mechanisms to penetrate gram-negative cell walls. Myoviruses use contractile tails to push a rigid tube through the outer membrane and periplasm. This allows the tail’s tip to create an opening in the cytoplasmic membrane. The linear, dsDNA genome then moves through this tube and into the cytoplasm [3,4,5]. Like myoviruses, podo- and siphoviruses use tail structures to breach outer membranes. However, their tails are non-contractile and do not fully cross the periplasm. Instead, proteins ejected from the virion assemble a conduit traversing the remainder of the cell wall [6,7]. By contrast, the tail-less, icosahedral leviviruses use an attachment protein to bind host pili [8]. Pilus retraction pulls the virion to the cell surface. Afterwards, the attachment protein with the bound RNA genome enters the cell, via an unknown mechanism [9,10]. Similarly, filamentous inoviruses initially attach to host pili [11]. Again, retraction brings the virion within reach of the cytoplasmic membrane. Although the mechanistic details are sparse, interactions between the virion and a host periplasmic protein allow the viral coat proteins to melt into the cytoplasmic membrane, while simultaneously delivering the ssDNA genome to the cytoplasm [12,13]. 

The icosahedral, tail-less, ssDNA-containing microviruses utilize a host penetration mechanism that combines many features seen in the aforementioned phages. Microviruses first attach to host lipopolysaccharides, triggering the removal of a spike protein G complex at the membrane-interacting vertex [14]. Whether the spike complex melds into the membrane, as is seen with inovirus coat proteins, is not known. A portion of the newly exposed coat protein pentamer then interacts with the host’s outer membrane. This interaction most likely triggers the conformational changes that open a pore at the virion’s 5-fold axis of symmetry, a structural change reminiscent of myovirus base plates during penetration. Then, like the sipho and podovirus ejection proteins, 10 copies of the DNA pilot protein H emerge from the capsid, to form a genome translocating channel that has been visualized traversing the cell wall [15].

Protein H is monomeric during early assembly and within the procapsid [16,17,18,19,20]; however, 10 of the encapsidated monomers must oligomerize for genome delivery [15]. Although an H oligomer appears to exist in unopened virions bound to LPS-containing membranes [14], it is unknown whether the monomers oligomerize into a core structure prior to host contact, like the structure seen within bacteriophage T7 virions [5]. After the virion opens, the H proteins exit the virion and form a genome translocation channel. Then, both the H proteins and the genome become associated with the host’s cytoplasmic membrane. Full genome transport appears to be coupled to the synthesis of the complementary genomic strand [21,22,23]. 

**Figure 1:**
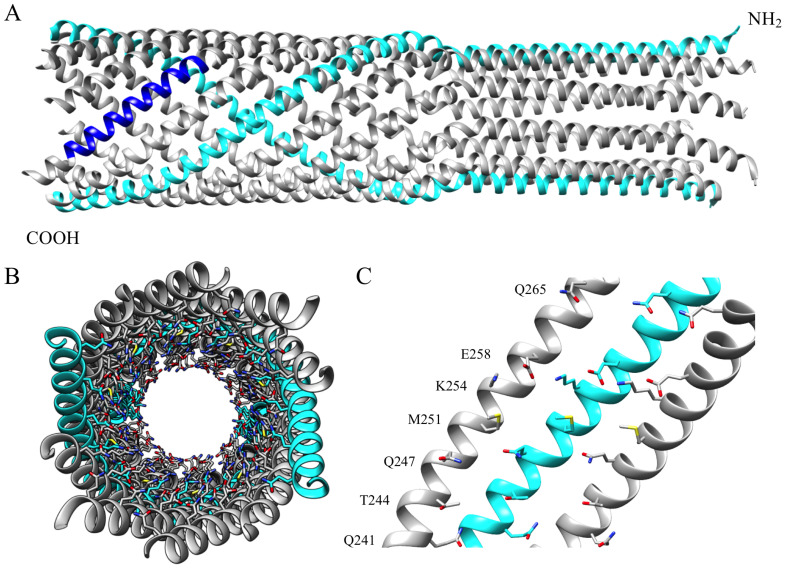
H-tube Structure. (**A**) The ϕX174 H protein’s coiled-coil domain when oligomerized as a decamer. The amino terminal end is facing up and two opposing monomers are colored in cyan (PDB: 4JPP). In one monomer, the region spanning Q241to Q265 is depicted in blue. (**B**) The tube, as seen from its C-terminal opening. Inward facing residues are modeled as sticks. (**C**) The inner surface of three α-helices between Q241 and Q265. Inward facing side chains are modeled as sticks. Inward facing side chain labels appear to the left of the helices.

An X-ray structure of the ϕX174 H protein’s central domain was solved to 2.4 Å resolution [15]. In the structure (Figure 1), H protein residues 151-271 form a decameric α-helical tube that is 170 Å long and 22–25 Å wide. H proteins likely adopt this structure during genome delivery, as the tube is long enough to span the host’s cell wall and wide enough to permit the passage of the circular genome’s two, antiparallel ssDNA strands [24]. The tube’s inner surface is primarily lined with amino acid side chains containing amide and guanidinium moieties. Notably, 18 of the 23 inward facing positions are occupied by asparagine, glutamine, and arginine side chains, with glutamine being the most abundant (Table 1). As these amino acids are frequently observed interacting with DNA nucleotides in other protein structures [25,26,27,28], by forming specific H-bonds with nitrogenous bases, they may facilitate the transit of the phage genome. This structural arrangement may be a general feature of biological conduits transporting nucleic acids. A recently reported structure of feline calicivirus bound to a portion of its receptor also features an α-helical tube with inward facing amide and guanidinium containing side-chains [29]. To test this hypothesis and explore the general role of the amide and guanidinium groups, the inward-facing amino acids in this region were mutated. Specifically, non-glutamine residues were changed to glutamine, and glutamine residues were changed to serine. The results of the subsequent genetic and biochemical analyses support a general model, in which the H-tube’s inner surface exerts a frictional force, or drag, on the passing viral genome. This may balance the force provided by capsid pressure and/or the osmotic gradient driving the genome into the cytoplasm, thereby regulating genome delivery. 

## 2. Materials and Methods 

### 2.1. Bacterial Strains, Phage Strains, and Plasmids 

The *Escherichia coli* C strains C122 (Su-), BAF8 (*supF*), and BAF30 (*recA*) have been previously described [30,31]. RY7211 contains a mutation in the mraY gene, which confers the resistance to viral E protein mediated lysis [Bernhardt, 2001 #396]. 

The Q241S, T244Q, Q247S, M251Q, K254Q, E258Q, Q265S, T244Q/M251Q, and Q241S/Q247S H genes were constructed by mutagenizing the previously described cloned ϕX174 H gene [32]. Abutting primers introducing the desired mutation(s) were used to PCR amplify the entire plasmid with Q5 DNA polymerase (New England Biolabs, Ipswich, MA, USA). The PCR product’s 5’ hydroxyl termini were phosphorylated with T4 polynucleotide kinase (New England Biolabs) and the resulting product was circularized with a T4 DNA ligase (New England Biolabs). Cloned nucleotide sequences were verified with direct DNA sequencing. 

Mutant phage strains *cs(H)T244Q*, *cs(H)M251Q*, *cs(H)T244Q/M251Q,* and *cs|ts(H)Q241S/Q247S* were generated via the recombination-rescue of gene H amber mutant, *am(H)M251*, with the T244Q, M251Q, T244Q/M251Q, and Q241S/Q247S H clones. Cells carrying mutant H constructs were infected with *am(H)M251*. Recombinants were then selected by plating the resulting progeny on C122 (Su-) and confirmed by sequencing the H genes. Strains *su(H)-F T204I*, *su(H)-F M330I*, *su(H)-F R386H*, *su(H)-F T204I/cs(H)T244Q/M251Q*, *su(H)-F M330I/cs(H)T244Q/M251Q*, and *su(H)-F R386H/cs(H)T244Q/M251Q* were generated via a site-directed mutagenesis, using purified phage ssDNA in the same manner used to create the mutant H constructs. 

### 2.2. Second-Site Genetic Analyses

Second-site suppressing mutations of the cold-sensitive (*cs*) phenotype were isolated in direct selections. *cs(H)T244Q*, *cs(H)M251Q*, and *cs(H)T244Q/M251Q* were first plated permissively on *E. coli* C122 at 37 °C. Plaques were allowed to develop and were collected and placed in 0.1 mL of water. These solutions were then plated at 22 °C, the restrictive temperature. Putative *cs*^+^ revertants were selected, isolated and re-plated at 22 °C to confirm the loss of the *cs* phenotype. *cs* revertant genomes were sequenced to identify the second-site suppressing mutation. Second-site suppressing mutations of the *cs|ts(H)Q241S/Q247S* temperature-sensitive (*ts*) phenotype were isolated by first plating *am(H)M251* on cells containing the Q241S/Q247S H clone at 33 °C. Independent recombinant plaques were propagated at 33 °C and *ts*^+^ revertants were then isolated at 42 °C.

### 2.3. Media, Buffers, Phage Plating Assays, and Mutant H Containing Particle Preparation

Media, buffers, phage plating assays, and phage stock preparation have been previously described [30]. Mutant H containing particles were generated in *E. coli C* strain RY7211, which is resistant to ϕX174-mediated lysis, carrying the cloned mutant H genes at 37 °C. Then, 100 mL of cells were grown to a concentration of 1.0 × 10^8^ cells/mL in TKY media (1.0% tryptone, 0.5% KCl, 0.5% yeast extract) and then infected with *am(H)M251* at a multiplicity of infection (MOI) of 1. At the time of infection, cultures were supplemented with 10 mM MgCl_2_ and 5.0 mM CaCl_2_, and cloned mutant H gene expression was induced by adding isopropyl β-d-1-thiogalactopyranoside (IPTG) to a final concentration of 40 μM. The cells were pelleted three hours after infection and resuspended in BE buffer (50 mM Na_2_B_4_O_7_/3.0 mM EDTA), at 1/100 the original culture volume. Egg white lysozyme was added to a final concentration of 1.0 mg/mL and the samples were incubated overnight at 4 °C. Afterwards, 150 μL chloroform was added and the lysates were vigorously vortexed. Cell debris was pelleted and the phage containing supernatant was collected.

### 2.4. Protein Electrophoresis, Rate Zonal Sedimentation, and Generation of ssDNA

The protocols for protein electrophoresis, rate zonal sedimentation, and ssDNA purification have been previously described [33,34,35].

### 2.5. Attachment, Eclipse, Coat Protein Quantification, and Burst Size Assays

Attachment and eclipse assays have been previously described [36,37]. Coat protein quantification as a surrogate for genome DNA delivery was performed as follows. RY7211 cells were grown 1.0 × 10^8^ cells/mL in TKY media, pelleted, and suspended in one tenth the original volume of HFB buffer [0.06 M NH4Cl/0.09 M NaCl/0.1 M KCl/0.1 M Tris-HCl (pH 7.4)/1.0 mM MgSO4/1.0 mM CaCl_2_] containing 10 mM MgCl_2_ and 5.0 mM CaCl_2_ at 4 °C. Phages were pre-attached to cells for 30 min at 4 °C. The cells and attached phage were pelleted and resuspended in chilled (4 °C) TKY media, containing 10 mM MgCl_2_ and 5.0 mM CaCl_2_. The samples were then diluted into TKY containing 10 mM MgCl_2_ and 5.0 mM CaCl_2_, prewarmed to 22 or 37 °C. At time points, 1.0 mL samples were collected, pelleted, and the pellets were frozen. After freezing, the pellets were suspended in 100 μL HFB. After this, 30 μL of the concentrated samples were run on SDS-PAGE gels as previously described [35]. SDS-PAGE gels were then stained with Coomassie brilliant blue and digitized with a LICOR scanner. Relative coat protein levels were determined by densitometry analysis using ImageJ software (NIH). Coat protein intensity was normalized to the intensity of the host protein band. 

Burst size assays followed the same protocol as the coat protein quantification assay, with the following changes. At time points, 1.0 mL samples were collected, pelleted, and suspended in 100 μL HFB. Then, 10 μL of the concentrated sample was diluted into 1.0 mL of BE buffer containing 1.0 mg/mL of lysozyme and incubated overnight at 4 °C. As the MOI of these infections were less than 1, bursts were calculated by dividing the time point’s total phage by the input phage.

### 2.6. Potassium Efflux Assays

K^+^ efflux experiments are based on those described by Cumby et al. [6] and adapted to microviruses [38]. Mutant H containing virions were generated as described above. The virions were dialyzed thrice against 1.0 L of SM buffer (100 mM NaCl, 10 mM MgSO_4_, 50 mM Tris pH = 7.5), at 4 °C to remove potassium. C122 was grown to 1.0 × 10^8^ cells/mL in TKY media at 37 °C. Cells were pelleted and washed in an equal volume of SM buffer, washed again in 1/10^th^ the volume SM buffer, and suspended in 1/100^th^ the volume iced SM+ buffer: SM buffer containing 10 mM MgCl_2_ and 5 mM CaCl_2_. The cell culture was divided into 1.0 mL aliquots, kept on ice, and the chilled virus was added to a MOI of 75. Iced SM+ buffer was added to equalize aliquot volumes. Infections were incubated for 15 min on ice, to allow virus pre-attachment. One at a time, infections were diluted into 9.0 mL 37 °C SM+ buffer every minute. Immediately after the first dilution, an Orion Ionplus potassium electrode (Thermo Scientific, Waltham, MA, USA) connected to a Sartorius PB-11 pH meter was inserted into an infection and a reading was taken after 1 min. This electrode was then rinsed with DI H_2_O, blotted dry, and placed into the dilution of the subsequent infection. The electrode was rotated between infections at one-minute intervals once all infections were diluted.

### 2.7. Statistical Analysis of the K^+^ Efflux Data

Raw K^+^ efflux data was modeled with a linear mixed effects model. Fixed effects were included for virus condition (levels: uninfected, wild-type, T244Q, M251Q, K254Q, and T244Q/M251Q), temperature (levels: 22 and 37°), and time. Because plots of the data showed a clear non-linear (half-parabolic) change in the extracellular K^+^ level over time, both linear and quadratic effects of time were incorporated into the model. Additionally, because different K^+^ efflux trajectories for each virus/temperature condition were desired, all fixed effect interactions were included. Finally, in order to capture some of the correlation structure likely present in the data due to the experimental design, two random intercepts were included: one for experiment day and another for the final test tube from which measurements were collected. Planned contrasts of the four mutant virus conditions versus both wild-type and uninfected were conducted at 25 and 150 min, in the 37 and 22° conditions, respectively.

The correlation and variance structure of the data are likely more complex than that described above. Firstly, there are actually four potential sources of random variation and consequent correlation in the data: virus batch, experiment day, original test tube, the tube before splitting into separate tubes for the two temperature conditions, and final test tube. Second, sequentially close measurements from the same final test tube are likely correlated. Third, evidence of temporal heteroscedasticity: variability of the random disturbance is different across elements of the vector, was revealed after modeling explorations. In order to determine whether incorporating this more complex variance and correlation structure into the model affected the contrast inferences, another model was fit to the K^+^ efflux data. It included an exponentially decreasing correlation and variance structures over time, and also random intercepts for virus batch, experiment day, original test tube, and final test tube. The fixed effects structure was identical to that of the simpler model, and the same planned contrasts were conducted. Because the two models’ inferential results were identical, the simpler model conclusions were presented. The complete details of the statistical analysis are provided in the Supplemental Material File S1.

### 2.8. Infected Cell Membrane Separations and qPCR

The membrane separation protocol was based on one described by Morein et al. [39] C122 was grown to OD_660_ = 0.1 in TKY at 37 °C. The cells were pelleted and washed in an equal volume of SM buffer, then concentrated 10-fold in SM buffer, and again concentrated 10-fold in iced SM+ buffer. The concentrated culture was divided into 2.0 mL aliquots and kept on ice. A chilled virus was added to a MOI of 1.0 and additional iced SM+ buffer was added to equalize infection volumes. The virus was allowed to pre-attach for 30 min at 4 °C. The infections were split and each half was diluted 10-fold in 37 or 22 °C SM+ buffer and incubated for 10 or 30 min, respectively. The infections were then diluted 10-fold in iced SM+ buffer, pelleted, and washed in 40 mL iced BE buffer. Pellets were suspended in 15 mL iced 10 mM Tris (pH = 7.5), containing 0.5 M sucrose. Lysozyme was added to a final concentration of 0.1 mg/mL. The infections were incubated for 30 min at 4 °C and were then slowly diluted (~3 mL/min) with 15 mL iced 10 mM Tris (pH = 7.5). EDTA was added to a final concentration of 1.0 mM and samples were sonicated in an ice bath with a Branson Sonifier 450. Samples were sonicated 15 s at a time, with a 45 s cool down between pulses. This cycle was repeated until the OD_600_ of the infection stopped decreasing. This took four to five cycles and an infection’s final OD_600_ was usually between 10 and 30% its original value. Samples were then spun at 1500× *g* for 10 min, to pellet any remaining intact cells. The supernatants were then centrifuged for 90 min at 257,000× *g* in a Beckman Type 70Ti rotor, to pellet membrane vesicles. The resulting supernatant was removed and the moist pellets were stored at 4 °C overnight in sealed containers. Membrane pellets were then suspended in 15 mL gradient buffer [10 mM Tris, 1 mM EDTA, 16.2% Percoll (*v*/*v*), pH = 7.5], with an 18-gauge syringe and vigorous vortexing. The mixtures were transferred to 15 mL glass corex tubes and spun for 50 min at 14,500× *g* in a Sorvall SA600 rotor. The resulting gradients were fractionated into 250 μL fractions from the bottom, with the peristaltic pump of a BioRad BioLogic LP connected to a model 2110 fraction collector. Fraction succinate dehydrogenase activity was tested with a Biovision succinate dehydrogenase activity colorimetric assay kit and KDO content was determined by the thiobarbituric acid method [40]. 

A qPCR analysis of fractions was performed on an Applied Biosystems 7300 Real-Time PCR System using Applied Biosystems SYBR Green PCR Master Mix. The thermal cycling program started with 2 min at 50 °C, followed by 3 min at 95 °C to activate the DNA polymerase. This was followed by 40 cycles of 95 °C for 15 s and 58 °C for 1 min. Absolute quantification was performed by generating a standard curve with purified ϕX174 ssDNA. Dissociation curves were performed to confirm primer fidelity.

## 3. Results

### 3.1. Mutating Inward Facing Residues Results in Temperature-Dependent Phenotypes

Amino acid side chains with amide and guanidinium groups; like those found in asparagine, glutamine, and arginine; can interact with nitrogenous bases [25,26,27,28]. Eighteen of the 23 side chains lining the H tube’s inner surface belong to N, Q, or R residues [15]. The remaining inward facing side chains, which lack amide or guanidinium groups, cluster within a 28 Å-long region near the structure’s C-terminus (Table 1, Figure 1C). This arrangement is highly conserved. Forty-two H protein sequences from all three ϕX174-like clades [41] were aligned using the PRALINE multiple sequence alignment program [42,43]. Of the seven inward facing residues, Q241, T244, Q247, and K254 are completely conserved, and the respective hydrophobic and acidic characteristics of M251 and E258 are maintained. By contrast, Q265 has been replaced with acidic residues in two clades. The high number of conserved residues suggests that this domain may regulate ssDNA transport. To test this hypothesis, codons T244, M251, K254 and E258 were individually mutated to glutamine codons in a cloned wild-type H gene, resulting in mutant constructs T244Q, M251Q, K254Q, and E258Q. A double mutant gene, T244Q/M251Q, was also constructed. The glutamine codons were mutated to serine codons, resulting in Q241S, Q247S, Q265S, and a double mutant, Q241S/Q247S. Glutamine → serine substitutions were chosen to maintain the polar nature of these side chain without introducing a charge. For all subsequent biochemical experiments, virions were generated by complementing a *null-H* mutant, *am(H)M251*, with cloned wild-type or mutant H genes. This complementation-dependent approach circumvents genetic differences arising from compensatory mutations that can accumulate during phage propagation. Thus, any phenotype or defect can be directly associated with the mutant H proteins. 

The mutant and wild-type genes were assayed for the ability to complement *am(H)M251* at 22, 37, and 42 °C (Table 2). The cloned K254Q, E258Q, and the three single Q→S genes complemented the *null-H* mutant at all temperatures. By contrast, the T244Q, M251Q, T244Q/M251Q, and Q241S/Q247S genes complemented poorly at 22 °C. The severity of the cold-sensitive (*cs*) phenotype varied. Plating efficiencies on M251Q, T244Q/M251Q, and Q241S/Q247S expressing cells was several orders of magnitude below those obtained at higher temperatures. Only a slight titer reduction was seen on cells expressing the T244Q gene at 22 °C; however, plaque size was greatly reduced. A similar phenomenon was also observed when plating on Q241S/Q247S expressing cells at 42 °C, indicating that this mutant is both *cs* and temperature-sensitive (*ts*).

### 3.2. Infectious Particles Containing Mutant H Proteins Are Produced at Restrictive Temperatures

Gene H mutations can prevent H protein incorporation during morphogenesis or result in H-containing, uninfectious particles [32,44]. To determine whether the H proteins were incorporated at 22 °C, lysis resistant cells expressing the wild-type, T244Q, M251Q, and T244Q/M251Q H genes were infected with *am(H)M251*. As stated above, this approach circumvented the possible effects of fitness-enhancing secondary mutations appearing within the phage genome. Thus, all other viral proteins should be identical. 

Lysis resistant cells and infections were prepared as described in Material and Methods. After a 5-min incubation at 37 °C to ensure rapid infection initiation, cultures were split, the infected cells were pelleted, resuspended in media pre-heated to 22 or 37 °C, and respectively incubated for 6 or 3 h. A similar protocol was followed with Q241S/Q247S expressing cells, with slight modifications to account for its dual *cs*/*ts* plating phenotype. For this experiment, cells were initially infected at 33 °C, split into three aliquots after a brief incubation, and pelleted. The pellets were then re-suspended in media at 22, 33, or 42 °C, and respectively incubated for 6, 4, or 3 h. Infected cell extracts were then applied to linear sucrose gradients and particles separated by rate zonal centrifugation. 

**Figure 2:**
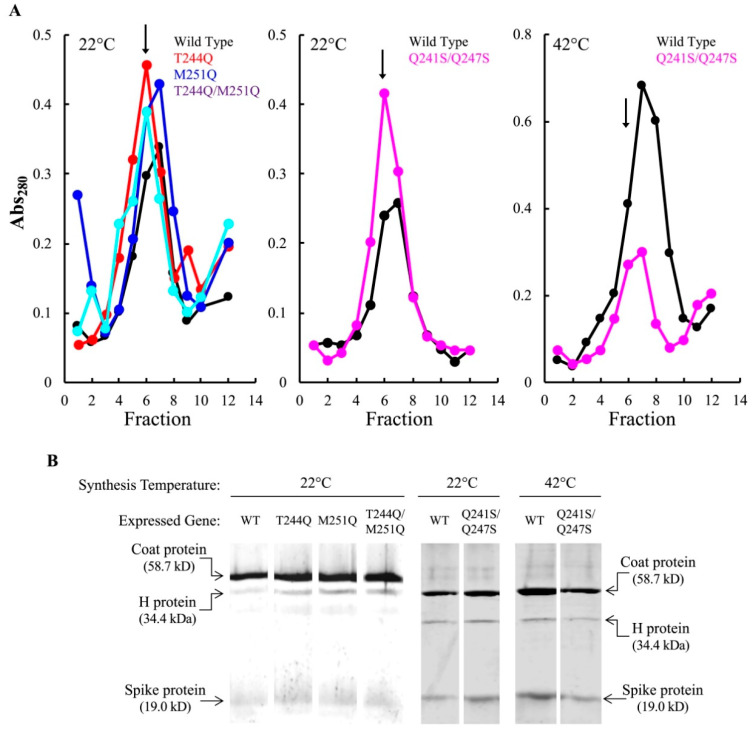
Assembled particles produced at restrictive conditions in mutant H expressing cells. (**A**) A_280_ profiles of infected cell extracts analyzed by rate zonal sedimentation. Fraction 1 represents the gradient bottom. The fraction with the highest specific infectivity (pfu/A_280_) is indicated with an arrow. Infection temperatures are indicated in the upper left of each graph. A color legend indicating the H gene allele resides in the upper right. (**B**) SDS-PAGE analysis of the fraction with the highest specific infectivity from panel (**A**). The white space between lanes indicates the removal of irrelevant lanes from the gel.

At the restrictive temperatures, each infection produced particles that sedimented at 114S, the S value of ϕX174 virions (Figure 2A). The restrictive sedimentation profiles did not significantly differ from those produced from permissive temperature infections (data not shown). The 114S peak fraction was run on an SDS-PAGE gel to determine protein content. The mutant H proteins were incorporated at levels comparable to the wild-type H protein (Figure 2B). Particle specific infectivity (plaque forming units/Abs_280_) was determined by titering the 114S particles at 37 °C on amber suppressing cells. Thus, any particle capable of infecting should produce a plaque. Although infectious particles were produced at all temperatures, the specific infectivity of mutant H protein particles was reduced compared to those containing the wild-type H protein (Table 3). The reduction was independent of synthesis temperature: populations synthesized at permissive and restrictive temperatures had nearly identical infectivity values. The greatest reductions were seen in the T244Q/M251Q and Q241S/Q247S H protein particles, which were less than 10% of their wild-type values. These data suggest that the primary defect involved mutant H protein function, and not H protein incorporation. 

### 3.3. Particles Containing Mutant H Proteins Do Not Efficiently Infect Cells at Restrictive Temperatures

At restrictive temperatures, DNA delivery can be indirectly monitored by observing the kinetics of viral coat protein accumulation within infected cells [32]. In all of the following experiments, virions were first produced in *am(H)M251* infected cells expressing the wild-type or mutant H genes. Each infection requires a specific multiplicity of infection (MOI), and this value was based on plaque forming units (PFUs), to control for the observed differences in specific infectivity. To monitor infection efficiency, *am(H)M251* virions carrying T244Q, M251Q, T244Q/M251Q, proteins were pre-attached to lysis resistant cells containing an inducible wild-type H gene. Differences in genome delivery can be attributed to the mutant H proteins, which were synthesized and incorporated into virions during the previous infection. After genome delivery, however, the *am(H)M251* mutation ensures that only wild-type H protein will be synthesized in infected cells, which carry a clone of the wild-type H protein. This strategy eliminates any differences that could arise from de novo H protein synthesis, which is required for optimal viral protein synthesis [32,45]. 

**Figure 3:**
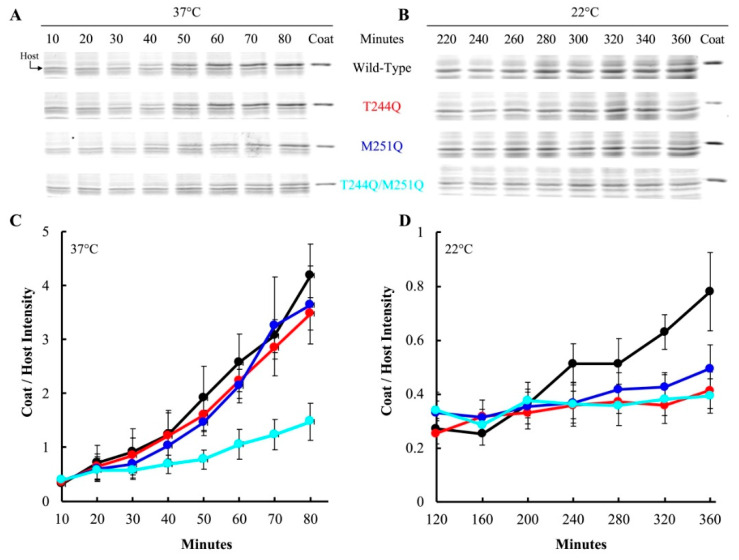
Viral coat protein levels in infected cells as a function of the infecting particles’ H protein. The infecting virions were generated in *am(H)M251* infected cells expressing different H gene clones. These particles were then used to infect lysis resistant cells expressing a clone of the wild-type H gene. (**A**,**B**) Representative SDS-PAGE analysis of infected cells incubated at 37 and 22 °C, respectively. (**C**,**D**) Coat to host protein ratios of infected cells seen in panels (**A**,**B**). The host protein band used in this analysis is indicated with an arrow in panel (**A**). A color legend indicating the H protein carried in the infecting virion is between panels (**A**,**B**). Error bars represent the standard error of the mean calculated from three biological replicates.

After pre-attachment, the infected cell culture was split and each aliquot was diluted into pre-heated media containing IPTG to induce the wild-type H gene. Infections with particles carrying the T244Q, M251Q, and T244Q/M251Q H proteins were diluted into media at 22 and 37 °C. At each time point, a sample was collected, the cells pelleted, and the whole cell lysates were run on an SDS-PAGE gel. The resulting gels were Coomassie stained and digitized. The intensity of the viral coat protein band was compared to a host protein band, to determine the relative amount of coat protein in each sample. Figure 3A,B depicts representative gels. Samples were taken throughout a time course in experiments were conducted with T244Q, M251Q, and T244Q/M251Q H containing particles, and the results of three biological replicates are plotted in Figure 3C,D. Virions carrying the wild-type H protein appeared to efficiently deliver their genomes at both 37 and 22 °C: viral coat protein levels started to respectively rise after 50 and 320 min, respectively. At 37 °C, no significant differences were observed between cells infected with T244Q, M251Q, and wild-type H protein containing virions. However, protein levels within the T244Q/M251Q infected cells appeared to be mildly reduced (Figure 3A,C). At 22 °C, viral coat protein accumulation at 22 °C was greatly reduced or entirely blocked in cells infected with T244Q, M251Q, and T244Q/M251Q H protein containing particles (Figure 3B,D). 

**Figure 4:**
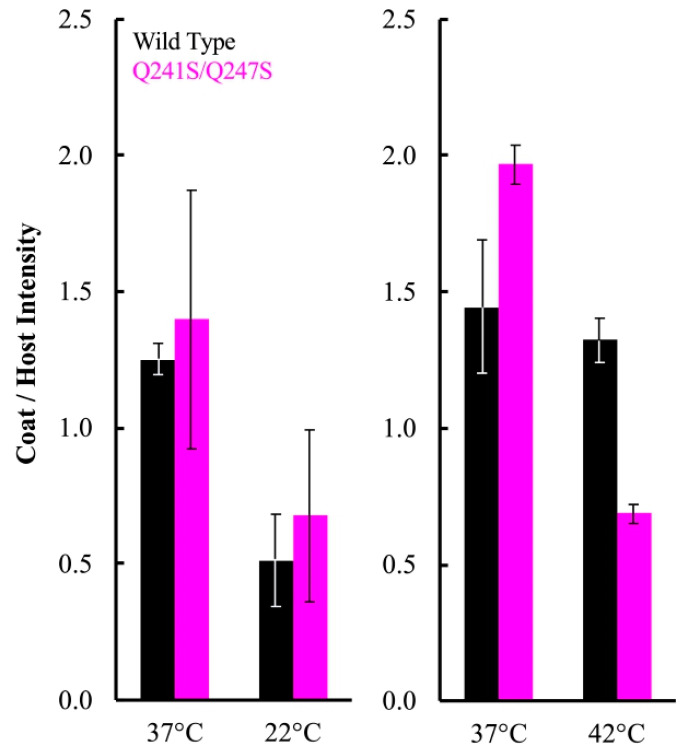
Coat to host protein ratios of cells as a function of the H protein within the infecting particle. The infecting virions were generated in *am(H)M251* infected cells, expressing different H gene clones. These particles were then used to infect lysis resistant cells, expressing a clone of the wild-type H gene. A color legend indicates the H protein found in the infecting particles. The infection temperatures are on the X-axes. Panel figures depict the results of two experiments conducted at one of the restrictive temperatures, 22 °C and 42 °C, each with a separate 37 °C control. Error bars represent the standard error of the mean calculated from three (**left**) or two (**right**) biological replicates.

A simplified version of this assay was repeated with particles containing the Q241S/Q247S H protein, to determine if genome transit was affected at 22 and 42 °C. The experimental details are similar to those described above, except that a single endpoint sample was taken. Infections at 22 and 42 °C were performed on separate days, and a 37 °C control infection was included in each experiment (Figure 4). Infection aliquots were incubated at 22 °C for 360 min, while those at 37 and 42 °C were incubated for 80 min. The infections were then pelleted and whole cell lysates were run on SDS-PAGE gels for a densitometric analysis (Figure 4). In contrast to the above results, there was not a significant difference in viral coat protein levels at 22 °C between cells infected with particles containing the wild-type H protein or the Q241S/Q247S H protein. However, significantly less coat protein was seen in cells infected by Q241S/Q247S H containing particles at 42 °C. These results suggest that the Q241S/Q247S H protein is fully functional at 22 °C in regards to DNA delivery, but this process is disrupted at 42 °C. Although suggestive, the experiments presented in Figure 3 and Figure 4 are based on the densitometry of SDS-PAGE gels, which can be error prone. Thus, alternative assays were developed and performed to confirm these results (see Section 3.5).

### 3.4. Mutant H Proteins Do Not Alter Attachment or Eclipse Kinetics at Temperatures Where Infection Is Inhibited

To initiate an infection, virions must first attach to and eclipse to host cells. Eclipse, also called irreversible attachment, is the committed step of early infection, during which virions undergo conformational changes and lose infectivity [14]. Defects affecting either event could produce the temperature-dependent phenotypes. Therefore, the kinetics of both extracellular steps were determined. 

**Figure 5:**
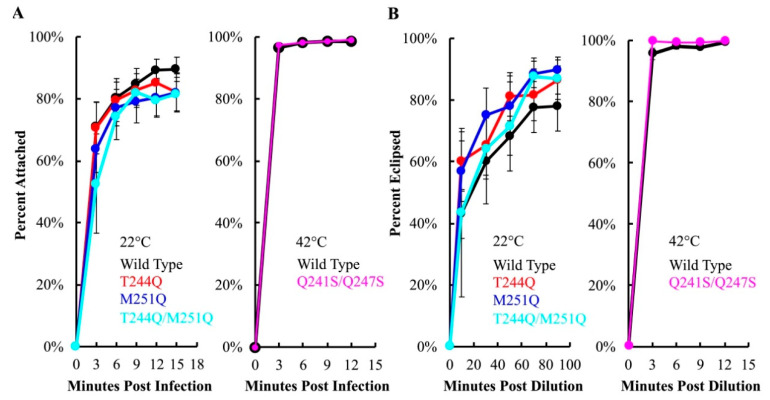
Attachment (**A**) and eclipse (**B**) kinetics of virions containing either the wild-type or mutant H proteins. A color legend and the assay temperature are provided in the lower right of each chart. Error bars represent the standard error of the mean calculated from either six biological replicates (22 °C attachment) or three biological replicates (all others).

To assay attachment kinetics, viruses and cells were mixed in liquid media containing 5.0 mM CaCl_2_, which is required for efficient attachment [46]. At selected times post infection, samples were centrifuged to separate attached and unattached particles. The latter remain in the supernatant, where they can be measured with a plating assay. No significant differences were observed between mutant and wild-type H-protein containing particles at 22 °C, or at 42 °C in the case of Q241S/Q247S H (Figure 5A). 

Eclipse kinetics were measured by pre-attaching virions at 4 °C, a temperature at which attachment occurs, but eclipse is inhibited. Subsequently, cells and attached phage were diluted into 22 or 42 °C media. At selected time points, attached particles were removed from cells by diluting samples into an EDTA solution. EDTA chelates the Ca^2+^ ions required for attachment. Thus, particles that have not eclipsed, and remain infectious, are released from cells. The proportion of uneclipsed, infectious particles was then determined by titering. Mutant eclipse kinetics did not significantly differ from those of the wild-type control at the assay temperatures (Figure 5B).

### 3.5. Temperature-Sensitive H-Tubes Inefficiently Open Cytoplasmic Channels, Whereas Cold-Sensitive H Proteins Open Cytoplasmic Channels but Inefficiently Transport DNA 

**Figure 6:**
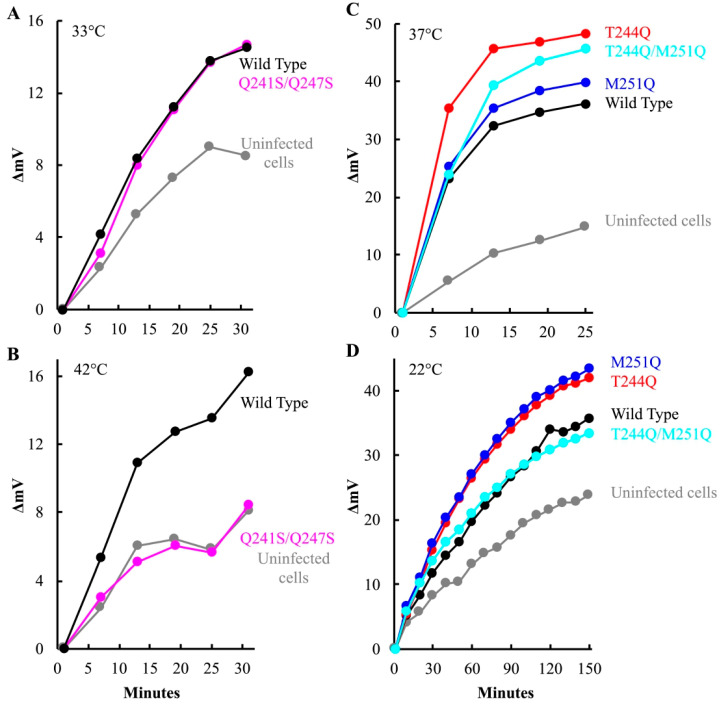
Potassium efflux curves produced by mutant H infected cells. Efflux values are reported as the increase in millivolts after the initial millivolt reading (ΔmV). Increasing positive values indicate increasing extracellular [K^+^]. The infection temperatures and H proteins found in the infecting virions are given in the figure.

After attachment and eclipse, the virus must open a channel to the cytoplasm, through which the infecting genome traverses. As with other phage systems, cytoplasmic K^+^ diffuses out of the cell through the genome delivery channel [6,38,47,48]. Thus, channel formation can be assayed by monitoring the release of intracellular K^+^ ions. To this end, *am(H)M251* virions containing mutant or wild-type H proteins were pre-attached to lysis resistant cells on ice. The infected cultures were split and diluted into K^+^ free starvation media, pre-warmed to the requisite temperature. A K^+^-selective electrode was used to monitor changes in extracellular K^+^ concentration. Representative plots are shown in Figure 6. K^+^ efflux triggered by particles carrying the Q241S/Q247S H protein was strongly temperature dependent. Efflux levels above the uninfected control were only observed at the lower temperature (Figure 6A,B). This suggests that the Q241S/Q247S H proteins either cannot open channels traversing the cell wall or cannot maintain the structure during genome transit at high temperature. By contrast, particles carrying the T244Q, M251Q, and T244Q/M251Q H proteins produced as much or more efflux at their restrictive temperature as virions carrying the wild-type protein, suggesting that the mutant H proteins can open a channel (Figure 6C,D).

At 22 °C, cells infected with T244Q and M251Q mutant particles exhibited more efflux than wild-type. The experiment in Figure 6D was conducted five times, and efflux triggered by the mutant H particles was always more robust when compared to the wild-type H control particles. The five biological replicates were analyzed with a linear mixed effects model (Material and Methods, Supplemental Material File S1). The results of this analysis suggest that the differences were statistically significant, at least by employed methodology (Discussion). The elevated efflux may be explained by using plaque assays to determine MOI. Mutant particles exhibit a lower specific infectivity than wild-type particles. Thus, although uninfectious, more H-containing particles were added, all of which may be capable of triggering K^+^ efflux.

Although particles carrying the T244Q, M251Q, and T244Q/M251Q H proteins can open channels traversing the host’s cell wall, the appearance of viral coat protein in infected cells was severely delayed. The delay could reflect slower delivery or less delivery. If fewer genomes are transported, it would take longer for coat protein levels to accumulate to detectable levels. If the mutant H proteins cause a significant portion of the infecting genomes to stall mid-transit, then a higher proportion of them may not reach the host’s inner membrane, the site of stage I DNA synthesis (ssDNA→dsDNA). To test this hypothesis, a qPCR analysis was performed on infected cell membranes fractions, outer versus inner, to track the infecting viral DNA. 

Before cells were infected at 22 or 37 °C, they were extensively washed in a starvation buffer (Materials and Methods) to inhibit de novo viral DNA replication. After a respective 10 or 30 min incubation at 37° and 22 °C, the infected cells were collected, washed twice to remove unattached and uneclipsed particles, converted to spheroplasts, and sonicated in the presence of EDTA. The resulting cytoplasmic and outer membrane vesicles were collected and separated in percoll gradients. Separation efficiency was determined by analyzing gradient fractions for succinate dehydrogenase activity and keto-deoxy-d-manno-8-octanoic acid (KDO) content, respective inner and outer membrane markers [39]. Fractions with the most KDO had a density of 1.03 g/mL. Those with the highest succinate dehydrogenase activity had a density of 1.01 g/mL. Both values were consistent with those previously reported when using this membrane separation protocol [39].

**Figure 7:**
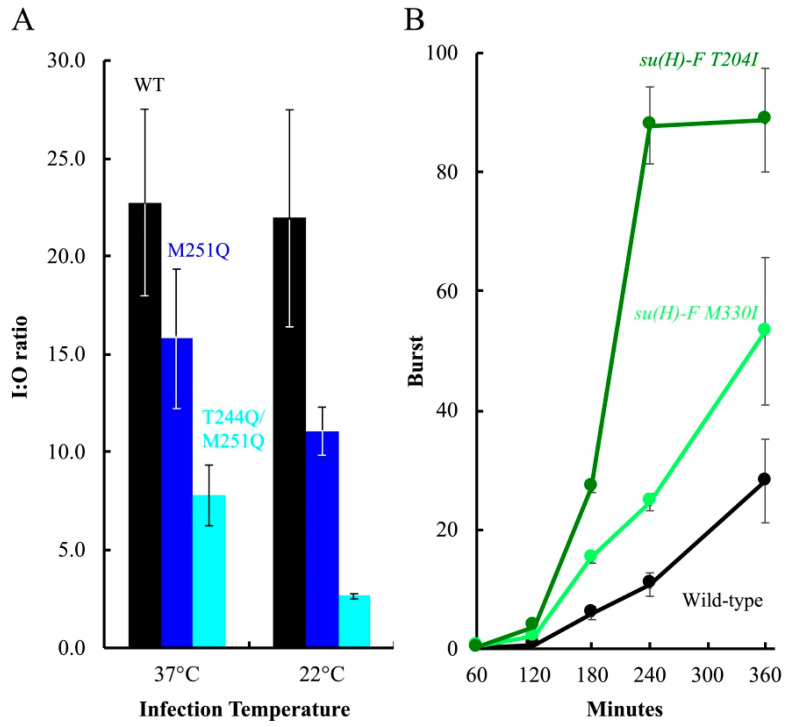
Infecting genome transit to the inner membrane and growth characteristics of the compensatory mutants. (**A**) Infecting genome transit to the inner membrane. Data are presented as I:O ratios, which is the genome copy number of the inner membrane over that of the outer membrane. I:O ratios of particles carrying wild-type H (black), M251Q H (blue), and T244Q/M251Q H (cyan) are shown. Error bars represent the standard deviation obtained from three technical replicates. (**B**) Growth characteristics of second site suppressors in an otherwise wild-type background. Titers of progeny produced in lysis-resistant cells infected with wild-type ϕX174 (black), *su(H)-F T204I* (dark green), or *su(H)-F M330I* (light green). Data are displayed as the viral burst size, which is calculated by dividing the total progeny by the total input phage. Experiments were conducted at MOIs less than 1.0 in lysis-resistant cells. Error bars depict the standard deviation obtained from three biological replicates.

The fractions were analyzed by qPCR to produce an inner/outer membrane genome copy-number ratio (I:O ratio). As depicted in Figure 7A, there was approximately 22-fold more wild-type genomic DNA associated with the inner membrane than the outer membrane at both temperatures (I:O ratio ≈ 22). Infecting cells with M251Q H containing particles resulted in lower I:O ratios, approximately 68 and 50% of the respective wild-type values at 37 °C and 22 °C. T244Q/M251Q H containing particles appeared to be even less efficient, producing ratios approximately 33% and 12% of the respective 37 °C and 22 °C wild-type values. As can be seen in Figure 7A, DNA transport was not entirely blocked for mutant H-tubes, especially at 22 °C. Several technical, structural, and biological factors may account for this (see Discussion). Nonetheless, these data indicate that DNA is less efficiently transported by the mutant H-tubes. 

### 3.6. Second-Site Genetic Analysis of Temperature-Dependent H Mutants

Second-site suppressing mutations often elucidate mechanisms by which the phage can overcome a defective process, providing mechanistic insights that are often difficult to obtain solely with biochemical data. To conduct the second-site reversion analysis, the T244Q, M251Q, T244Q/M251Q, and Q241S/Q247S mutations were moved into the phage genome by recombination rescue. As expected, each resulting mutant exhibited its respective *cs* or *ts*/*cs* phenotype (Table 4). The mutants were plated at their restrictive temperatures to select for suppressors. To ensure the recovery of a broad suppressor array, 26 independent sources of *cs(H)T244Q* and *cs(H)M251Q* were used in the analysis, along with 19 independent sources of *cs(H)T244Q/M251Q* and 12 of *cs|ts(H)Q241S/Q247S* [49]. The recovered suppressors are listed in Table 4. Eight unique mutations suppressing the *cs(H)T244Q* and *cs(H)M251Q* phenotypes were found in the coat protein gene F, and four in the piloting protein H gene. Two unique suppressors of *cs(H)T244Q/M251Q* and were recovered in gene H, while seven were recovered in gene H at 42 °C in the *cs|ts(H)Q241S/Q247S* background. The suppressors of *cs(H)T244Q*, *cs(H)M251Q*, and *cs(H)T244Q/M251Q* in gene H confer substitutions near the narrowest region of the H-tube X-ray structure (Figure 1A and Table 1), while those suppressing *cs|ts(H)Q241S/Q247S* tend to occur near the parental mutations. These likely function by altering H protein structure (see Discussion). The high temperature suppressors of *cs|ts(H)Q241S/Q247S* did not restore plaque formation at 22 °C (Table 4). Low temperature revertants could not be isolated for this mutant above a frequency of 2 × 10^−8^, suggesting that multiple changes are required to correct the defect. 

The coat protein substitutions isolated in the *cs(H)T244Q* and *cs(H)M251Q* backgrounds occurred near, adjacent to, or at sites previously isolated when selecting for suppressors of *cs* assembly defects. Two of the identified substitutions, T204I and M330I, were identical to previously isolated suppressors [30,34,50,51]. Although particle assembly and DNA transport are seemingly unrelated phenomena, second-site suppressor isolation is based on plaque formation, which can be affected by modest burst size increases. Thus, broad allele specificity can reflect general suppressing mechanisms [52,53]. To test this hypothesis, the F-T204I and F-M330I were moved into the wild-type background and the kinetics of phage production was monitored at 22 °C (Figure 7B). The burst size of both *su(H) F*-*T204I* and *su(H) F*-*M330I* were significantly higher than the wild-type control. 

The ability to rescue via a general mechanism may be related to the severity of the parental defect. The *cs* phenotype of the double mutant is significantly tighter than both single mutants. Suppressing mutations in gene F were only recovered for the single mutants. This could reflect a limited sample pool, as it is unlikely that the selections went to saturation, or it could be directly related to the defect severity conferred by the parental mutations. To distinguish between these two possibilities, the suppressors in gene F were moved into the *cs(H)T244Q/M251Q* background. As can be seen in Table 4, the suppressing mutations in gene F were unable to rescue the double mutant.

## 4. Discussion

### 4.1. Additional Glutamines, H-Tube Structure, and Defective Function

The H-tube’s inner surface is lined with side chains containing amide and guanidinium groups. However, there are four exceptions near the tube’s C-terminal end: T244, M251, K254, and E258 (Figure 1 and Table 1). This C-terminal “amide bald spot” is conserved across the three ϕX174-like clades, suggesting its requirement for optimal function. These four residues were mutated to glutamine. The K254Q and E258Q mutations did not result in a discernable phenotype, whereas the T244Q and M251Q mutations conferred *cs* phenotypes when expressed from plasmids in complementation studies or when place directly within the phage genome. The double mutant, T244Q/M251Q, displayed a particularly pronounced *cs* phenotype. The H-tube atomic structure may explain the phenotypic differences associated with these four sites. Unlike T244 and M251, K254 and E258 form an inter-helical salt bridge [15]. Thus, their side chains may not be entirely free to interact with the DNA as it passes through the tube. Moreover, glutamine can act as both a hydrogen bond donor and acceptor. Replacing K254 or E258 with glutamine may replace the inter-helical salt bridge with an inter-helical hydrogen bond.

The viral life cycle was investigated to determine which steps were affected by the T244Q, M251Q, and T244Q/M251Q substitutions. At 22 °C, the mutant H proteins were incorporated into virions as efficiently as wild-type. The resulting particles also displayed wild-type attachment and eclipse kinetics at the restrictive temperature. Lastly, the results of K^+^ efflux experiments indicate that they form channels through host cell walls. However, the viral DNA does not appear to efficiently reach the cytoplasmic membrane, the site of stage I viral DNA replication. 

To the best of our knowledge, tracking infecting genomes between separated cell wall components via qPCR has not been previously attempted in other phage systems. Thus, the extent that the results can be directly related to phenotype severity is not entirely known. The M251Q and T244Q/M251Q mutants have strong cold-sensitive phenotypes and the temperature affected the results of the qPCR assay. With the wild-type H protein, the same amount of genomic DNA became associated with the cytoplasmic membrane, regardless of temperature. By contrast, the samples generated with mutant H proteins exhibited a marked decrease of inner membrane associated viral DNA at 22 °C, which is consistent with a *cs* phenotype. However, inner membrane genome association was not entirely eliminated. Indeed, more DNA was consistently detected in the inner membrane fraction. For example, the I:O ratio for the T244Q/M251Q mutant at 22 °C was approximately 2, indicating that twice as much genomic DNA was detected in the inner membrane fraction. By comparison, the I:O ratio for wild-type was an order of magnitude higher. 

Several factors may affect I:O ratios. Firstly, cell wall fractionation is a messy affair; definitely not for the faint hearted or those distressed by impurities. The molecular markers used to distinguish between inner and outer membrane fractions typically differ by only 5–10 fold, regardless of protocol [39], indicating a significant amount of cross contamination. Secondly, it is unknown into which fraction the genome will segregate if it is within the H-tube, which spans the cell wall. Thirdly, the requisite experimental conditions under which the assay had to be performed differ from those occurring during a typical infection. However, all of these phenomena would equally affect wild-type and mutant samples. Thus, the results likely reflect the nature of the defective phenotype. 

### 4.2. Glutamine Removal, Tube Structure, and Defective Function

Surrounding and within the analyzed 28 Å region are three glutamine residues, Q241, Q247, and Q265 (Figure 1C). Of the three, Q241 and Q247 are perfectly conserved across the three ϕX174-like clades, while Q265 is replaced with a glutamic acid in other clades. These residues were replaced with serine to determine what effect, if any, amide removal has on genome delivery. Individual serine mutations at any one of the targeted sites did not alter plaque formation. The Q241S/Q247S mutation, however, conferred a *ts*/*cs* plating phenotype. This difference may be due to the tube’s structural redundancy. Since amide and guanidinium side chains line the majority of the tube’s lumen, mutations that only remove a single ring of glutamine side chains may not drastically change the lumen’s characteristics vis-à-vis genome transport. The removal of two sequential rings, by contrast, may reduce DNA-lumen interactions enough to disrupt DNA transport at high temperatures. The Q241S/Q247S mutation appears to inhibit channel formation at high temperatures. The prerequisite events, host attachment and infectivity loss, appear to occur at approximately the same rate as wild-type virions. This leaves three possible explanations for the *ts* phenotype: the virions may fail to eject their mutant H proteins, the H proteins may enter the cell wall but fail to open a channel, or the H proteins do open a channel but it is not integrous, and therefore cannot contain the transiting genome at 42 °C. 

Although channel formation was disrupted at 42 °C, our data show that the Q241S/Q247S H tube can be used to deliver genomic DNA at 22 °C. The defect preventing plaque formation at low temperatures was not definitively identified; however, the defect does not appear to strongly affect attachment, eclipse, channel formation, genome transport, or assembly. The effects conferred by the two mutations in *cis* could be pleiotropic, having small effects on several H-protein associated functions. However, assays examine each function individually, which requires the mutant to have a primary defect to be strong enough to be detected in at least one assay. 

### 4.3. The Function of the Amide and Guanidium Lined Surface

To some extent, the experimental results can be interpreted within a model in which a balance of opposing forces regulates genome movement. One force propels the genome inward. This could be driven by capsid pressure, created by the densely packaged viral genome, or the osmotic gradient existing between the host’s cytoplasm and the environment, or a combination of the two [1,2]. The opposing force may be friction or drag created by the H tube’s inward facing amide and guanidinium groups, which form hydrogen bonds with DNA nucleotides [25,26,27,28]. 

While invoking a force that opposes genome delivery seems counter-intuitive, systems more effectively perform work when potential energy is released in small, manageable increments. Hydroelectric dams, nuclear power plants, internal combustion engines, and oxidative phosphorylation, in which electrons are transferred from NADH to O_2_ via several intermediate reactions, all demonstrate this concept: releasing too much potential energy at one time can destructively overwhelm a system. Our results are consistent with this model and the observed temperature-dependent phenotypes. The additional amide groups, via interactions with DNA, may be increasing the frictional force. The removal of these groups, by contrast, may decrease this force. Hydrostatic and osmotic pressures are directly proportional to temperature. Thus, the force driving the genome into the cell may be decreased at a lower temperature. This, combined with the increased drag introduced by the additional amide groups, could perturb the optimal balance between the two forces, either kinetically trapping the genome or greatly slowing its delivery. In the reverse situation, in which amide groups are removed and temperature is high, the ssDNA may move through the tube too quickly. This may overwhelm the tube’s integrity. In an alternative model, the addition or removal of amide groups could have unanticipated effects on the H-tube’s structure, which could produce the same phenotype. Regardless of the molecular mechanism, the defect occurs while the cell wall is breached. 

The results of the K^+^ efflux studies indicate that the *cs* H proteins breached the inner membrane and the relative magnitude of phage-induced efflux at 22 °C was dependent on the H protein. This observation was found to be significant after constructing a linear mixed effects model built from five replicate data sets. To the best of our knowledge, a similar statistical analysis has not been conducted. Although a trend may be clearly visible in every experiment, a simple statistical analysis utilizing calculated means and error bars is difficult to apply, due to experimentally introduced variability between days. For example, the electrode’s scale is different every day it is turned on. K^+^ electrodes are extremely sensitive to NH_4_^+^ ions, a standard nitrogen source in biological media, to values as low as 2 ppm. Cells must be freshly prepared each time and the phage must be extensively dialyzed. Thus, different media and different stock solutions could be used on different days. This introduces a great deal of variation between days, but measurements taken on a single day with a single cell preparation are free of this problem. Consequently, to explore a consistent trend a more complicated and novel analysis needed to be conducted (Supplemental Results, Supplemental Material File S1). Thus, the biological significance of this observation cannot be rigorously adjudicated. Nonetheless, some speculation may be warranted. 

Single mutants effluxed more K^+^ than wild-type at 22 °C, whereas double mutant efflux resembled the wild-type. For the single mutants, this may be due to reduced specific infectivity. MOI was based on PFUs. Thus, if a population of virions had a reduced specific infectivity, as was observed for the mutants, more non-plaque forming, virus-like particles were added. These particles may still be capable of forming effluxing channels. However, particles containing the T244Q/M251Q H protein, which effluxed K^+^ like wild-type, exhibited one of the lowest specific infectivity values. It also exhibited the strongest *cs* phenotype and transported less DNA to the cytoplasmic membrane. Thus, an additional variable may be influencing this phenomenon, one directly related to the efflux through any one H-tube. The two extra glutamine residues within T244Q/M251Q tubes may grip the DNA so tightly that the tube becomes clogged or obstructed. Alternatively, as mentioned above, the added amide groups could have unanticipated effects on the H-tube’s structure, which may cause a percentage of double-mutant H-tubes to collapse.

### 4.4. Synergistic Effects and Compensatory Mechanisms

The results of the second-site genetic analysis further underscore the synergistic effects conferred by the two mutations and the mechanisms by which they can compensated. The *cs* phenotype of the single mutants *cs(H)T244Q* and *cs(H)M251Q* could be suppressed by intergenic and intragenic mechanisms, i.e., alterations to the viral coat or DNA pilot proteins, respectively. By contrast, only the intragenic mechanism appears to operate in the *cs(H)T244Q/M251Q* double mutant background. The suppressors in gene H confer changes at the H-tube’s narrowest point. In general, these mutations decrease side chain size, but did not radically alter chemical characteristics. The changes could be increasing the tube’s diameter and/or its elasticity. Suppressors of the *cs|ts(H)Q241S/Q247S* at 42 °C were also similar to those of *cs(H)T244Q/M251Q*, in that only intragenic mutations were recovered. In this case, suppressors clustered within and around the parental Q241S/Q247S mutations. Although the suppressing mutant residues’ hydropathic character was usually preserved, there was not a strong pattern vis-à-vis the mutant residue’s size or shape. Regardless of the precise suppression mechanism, the suppressing mutations within H are likely alleviating inhibited genome delivery by directly modifying the H-tube structure. 

In contrast to the intragenic suppressors, intergenic suppressors altering the viral coat protein appear to compensate for inhibited genome delivery instead of correcting it. These suppressors were similar or identical to previously isolated suppressors of *cs* assembly defects. When moved into the wild-type background, they increased the viral burst size at 22 °C. Thus, increasing the burst size may be compensating for inhibited genome delivery, which can be explained by the mechanics of plaque formation. As evinced by the leaky phenotypes of the single mutants, e.g., plating efficiencies of ~10^−2^ at 22 °C, DNA delivery is not entirely inhibited. However, to form a plaque, enough first-round progeny must successfully infect neighboring cells: a situation made more probable by a larger burst size. As can be seen in the piloting assay results, the *cs(H)T244Q/M251Q* phenotype is quite tight when compared to the single mutants. Its ability to associate DNA with the inner membrane is pronouncedly less efficient. Thus, its DNA delivery success rate may be too low and can only be rescued by an unobtainable burst size. Accordingly, when the suppressors found in the coat protein were placed directly within the double mutant background, they did not rescue on the level of plaque formation. 

### 4.5. Relationship to Other Phage Systems

Evoking a force that naturally counters the one propelling the genome into the cell seems counterintuitive. However, slow, regulated penetration may be common in other phage systems in which large amounts of potential energy, in the form of capsid pressure and/or an osmotic imbalance, must be controlled. For example, the complete transit of the phage λ genome requires approximately five minutes in vivo [54]. Moreover, in some phage systems, potential energy may not drive the entire genome into the cell. The efficient transfer of ϕX174 ssDNA appears to be coupled to the synthesis of the complementary genomic strand [21,22,23]. Thus, the H-tube may not be intended to deliver the entire genome to the host. The friction produced by the amide and guanidinium groups, or drag, may prevent complete transfer, so that only a small portion is presented to host DNA synthesis machinery. DNA penetration is often incomplete if hosts are not actively synthesizing DNA. The energy released by the hydrolysis of the dNTP’s during DNA synthesis is greater than the potential energy stored within the newly formed bonds of the DNA’s phosphate background. Thus, microvirus genome penetration may have evolved to exploit the unharnessed energy released in nucleic acid biosynthesis. Phages T5 and T7 may be other examples of phages that exploit the unharnessed energy released in cellular processes to perform work. They release their genomes in discrete steps [55,56] and internalization of the entire T7 or T5 genome may be facilitated by host transcription or translation machinery, respectively [57,58]. 

## Figures and Tables

**Table 1:** Primary sequence of the coiled-coil domain.

Position ^1^	Sequence ^2^	Name ^3^
151	QKELTKMQLDN	Hendecad 1
162	QKEIAEMQNET	Hendecad 2
173	QKEIAGIQSAT	Hendecad 3
184	SRQNTKDQVYA	Transition
195	QNEMLAY	Heptad 1
202	QQKESTA	Heptad 2
209	RVASIME	Heptad 3
216	NTNLSKQ	Heptad 4
223	QQVSEIM	Heptad 5
230	RQMLTQA	Heptad 6
237	QTAGQYF	Heptad 7
244	TNDQIKE	Heptad 8
251	MTRKVSA	Heptad 9
258	EVDLVHQ	Heptad 10
265	QTQNQRY	Heptad 11

^1^ H protein residues 151–271 form a long coiled-coil structure, consisting of 7,2 and 11,3 a-helices. Numbers refer to amino acid positions within the primary sequence. ^2^ Residues with inward facing side chains are colored red or blue. Blue residues were mutated in this study. ^3^ The name of the 11,3 (hendecad) and 7,2 (heptad) a-helices. Although helical, the transition region is kinked.

**Table 2:** *am(H)* plating efficiencies on cells expressing gene H clones.

Efficiency or Plating (EOP ^1^)			
Expressed H Gene	22 °C	37 °C	42 °C
Wild-Type	1.0	1.0	1.0
None	<1.3 × 10^−4^	<9.0 × 10^−5^	<4 × 10^−5^
T244Q	0.3 ^2^	1.0	0.5
M251Q	<1.3 × 10^−4^	1.9	0.7
K254Q	0.7	1.0	0.5
E258Q	0.8	2.8	0.6
T244Q/M251Q	<1.3 × 10^−4^	0.7	0.8
Wild Type	1.0	1.0	1.0
None	<4.1 × 10^−5^	1.3 × 10^−6^	<1.5 × 10^−6^
Q241S	0.1	0.8	0.7
Q247S	0.1	0.9	0.6
Q265S	0.4	0.8	0.5
Q241S/Q247S	<4.1 × 10^−5^	0.6	0.5 ^2^

^1^ Efficiency of plating: values are normalized to those obtained from cells expressing the wild-type H gene. Normalized values were generated by comparing EOP data generated on the same day, using the same plating cells. ^2^ Plaque size reduced by >50% of wild type.

**Table 3:** Specific infectivity ^1^ of 114S particles at 37 °C.

Synthesis Temperature	Expressed Gene	Specific Infectivity	
		Raw S.I. ^2^	Wild-Type Normalized ^3^
22 °C	Wild-Type	2.6 (±0.5) × 10^12^	1.0 (±0)
	None	3.3 (±1.6) × 10^8^	1.3 (±0.7) × 10^−4^
	T244Q	1.0 (±0.1) × 10^12^	0.4 (±0.1)
	M251Q	8.5 (±1.3) × 10^11^	0.4 (±0.1)
	T244Q/M251Q	1.7 (±0.4) × 10^11^	7.0 (±2.5) × 10^−2^
37 °C	Wild-Type	6.9 (±0.7) × 10^11^	1.0 (±0)
	None	3.1 (±1.1) × 10^8^	4.3 (±1.3) × 10^−4^
	T244Q	1.8 (±0.2) × 10^11^	0.3 (±0.02)
	M251Q	2.0 (±0.3) × 10^11^	0.3 (±0.1)
	T244Q/M251Q	4.6 (±0.7) × 10^10^	6.7 (±1.3) × 10^−2^
22 °C	Wild-Type	3.3 (±0.2) × 10^12^	1.0 (±0)
	None	4.5 (±1.7) × 10^8^	1.4 (±0.5) × 10^−4^
	Q241S/Q247S	2.9 (±0.9) × 10^11^	8.8 (±2.9) × 10^−2^
33 °C	Wild-Type	1.2 (±0.3) × 10^12^	1.0 (±0)
	None	2.0 (±1.3) × 10^6^	1.6 (±0.8) × 10^−6^
	Q241S/ Q247S	2.4 (±0.5) × 10^10^	1.9 (±0.3) × 10^−2^
42 °C	Wild-Type	5.8 (±0.2) × 10^11^	1.0 (±0)
	None	1.6 (±1.4) × 10^6^	2.8 (±2.3) × 10^−6^
	Q241S/Q247S	1.2 (±0.4) × 10^10^	2.1 (±0.8) × 10^−2^

^1^ Specific infectivity is defined as pfu’s/A_280_, regardless of the plaque forming particle’s genotype. ^2^ Raw S.I. reported in units of pfu’s/A_280_. Error values represent the standard deviation of three replicates. ^3^ Data are normalized to the specific infectivity of particles generated on the same day and in cells at the same temperature expressing the wild-type H gene. Error values represent the standard deviation of three replicates.

**Table 4:** Suppressing Mutations of H protein defects.

Strain ^b^	Independent Isolations ^c^	EOP ^a^		
		22 °C	33/37 °C	42 °C
Wild Type	NA ^d^	0.2	1.0	0.7
*cs(H)T244Q*	NA	4.8 × 10^−2^	1.0	1.0
*su(H)-F H73Y/cs(H)T244Q*	2	0.3	1.0	1.2
*su(H)-F Y158H/cs(H)T244Q*	3	0.2	1.0	0.7
*su(H)-F T204I/cs(H)T244Q*	1	0.2	1.0	1.3
*su(H)-F M330I/cs(H)T244Q*	1	0.3	1.0	0.9
*su(H)-F R386H/cs(H)T244Q*	1	0.3	1.0	0.9
*su(H)-H R185H/cs(H)T244Q*	1	1.0	<6.4 × 10^−5^	<6.4 × 10^−5^
*su(H)-H A194S/cs(H)T244Q*	1	0.2	1.0	1.0
*cs(H)M251Q*	NA	2.6 × 10^−2^	1.0	1.0
*su(H)-F T100A/cs(H)M251Q*	1	0.1	1.0	1.6
*su(H)-F Y158H/cs(H)M251Q*	1	0.3	1.0	1.2
*su(H)-F T204I/cs(H)M251Q*	1	0.2	1.0	1.1
*su(H)-F L319F/cs(H)M251Q*	1	0.1	1.0	0.6
*su(H)-F M330I/cs(H)M251Q*	2	0.3	1.0	0.8
*su(H)-F V333F/cs(H)M251Q*	1	0.1	1.0	1.2
*su(H)-F R386H/cs(H)M251Q*	1	0.1	1.0	0.7
*su(H)-H E197D/cs(H)M251Q*	1	0.4	1.0	1.1
*su(H)-H M198V/cs(H)M251Q*	1	0.5	1.0	0.9
*cs(H)T244Q/M251Q*	NA	<9.5 × 10^−6^	1.0	1.2
*su(H)-H M198I/cs(H)T244Q/M251Q*	1	0.1	1.0	1.2
*su(H)-H M198V/cs(H)T244Q/M251Q*	2	0.2	1.0	0.9
*su(H)-F T204I/cs(H)T244Q/M251Q*	SD ^d^	<1.9 × 10^−3^	1.0	<1.9 × 10^−3^
*su(H)-F M330I/cs(H)T244Q/M251Q*	SD ^d^	<6.9 × 10^−6^	1.0	0.5
*su(H)-F R386H/cs(H)T244Q/M251Q*	SD ^d^	<6.0 × 10^−6^	1.0	1.0
*cs|ts(H)Q241S/Q247S*	NA	<2.5 × 10^−4^	1.0	<2.5 × 10^−4^
*su(H)-H N216D/cs|ts(H)Q241S/Q247S*	1	<0.2	1.0	0.8
*su(H)-H T234I/cs|ts(H)Q241S/Q247S*	2	<0.05	1.0	0.9
*su(H)-H A239V/cs|ts(H)Q241S/Q247S*	1	<1.8 × 10^−5^	1.0	0.3
*su(H)-H Q235K/cs|ts(H)Q241S/Q247S*	1	<0.2	1.0	2.2
*su(H)-H F243L/cs|ts(H)Q241S/Q247S*	3	<8.0 × 10^−4^	1.0	0.6
*su(H)-H Q247L/cs|ts(H)Q241S/Q247S*	8	<1.0 × 10^−4^	1.0	1.2
*su(H)-H V255I/cs|ts(H)Q241S/Q247S*	1	<2.0 × 10^−3^	1.0	0.5

^a^ Efficiency of plating. Values are normalized to those obtained at 37 °C for *cs* mutants, and at 33 °C for *cs|ts* mutants. ^b^ Suppressing mutations are named with the gene in which the nucleotide substitution was found and the resulting amino acid change. Thus, *su(H)-F H73Y* indicates a suppressor of an H defect in gene F, conferring an H73Y substitution at amino acid 73. ^c^ For *cs* mutants, revertant plaques were picked from plates incubated at 22 °C. For *cs|ts* mutants, revertant plaques were picked from plates incubated at 42 °C. ^d^ NA: not applicable, parental strain.

## Supplementary Materials

The following are available online at can be found at https://www.mdpi.com/1999-4915/12/6/670/s1. File S1: A detailed explanation of the statistical analyses conducted on K^+^ efflux curves.

## References

[1] Evilevitch A. (2013). Physical evolution of pressure-driven viral infection. Biophys. J..

[2] Molineux I.J., Panja D. (2013). Popping the cork: Mechanisms of phage genome ejection. Nat. Rev. Microbiol..

[3] Aksyuk A.A., Leiman P.G., Kurochkina L.P., Shneider M.M., Kostyuchenko V.A., Mesyanzhinov V.V., Rossmann M.G. (2009). The tail sheath structure of bacteriophage T4: A molecular machine for infecting bacteria. EMBO J..

[4] Fokine A., Rossmann M.G. (2014). Molecular architecture of tailed double-stranded DNA phages. Bacteriophage.

[5] Hu B., Margolin W., Molineux I.J., Liu J. (2013). The bacteriophage t7 virion undergoes extensive structural remodeling during infection. Science.

[6] Cumby N., Reimer K., Mengin-Lecreulx D., Davidson A.R., Maxwell K.L. (2015). The phage tail tape measure protein, an inner membrane protein and a periplasmic chaperone play connected roles in the genome injection process of E. coli phage HK97. Mol. Microbiol..

[7] Jin Y., Sdao S.M., Dover J.A., Porcek N.B., Knobler C.M., Gelbart W.M., Parent K.N. (2015). Bacteriophage P22 ejects all of its internal proteins before its genome. Virology.

[8] Dent K.C., Thompson R., Barker A.M., Hiscox J.A., Barr J.N., Stockley P.G., Ranson N.A. (2013). The asymmetric structure of an icosahedral virus bound to its receptor suggests a mechanism for genome release. Structure.

[9] Krahn P.M., O’Callaghan R.J., Paranchych W. (1972). Stages in phage R17 infection. VI. Injection of A protein and RNA into the host cell. Virology.

[10] Toropova K., Stockley P.G., Ranson N.A. (2011). Visualising a viral RNA genome poised for release from its receptor complex. J. Mol. Biol..

[11] Russel M., Model P., Calendar R. (2006). Filamentous Phage. The Bacteriophages.

[12] Bennett N.J., Gagic D., Sutherland-Smith A.J., Rakonjac J. (2011). Characterization of a dual-function domain that mediates membrane insertion and excision of Ff filamentous bacteriophage. J. Mol. Biol..

[13] Bennett N.J., Rakonjac J. (2006). Unlocking of the filamentous bacteriophage virion during infection is mediated by the C domain of pIII. J. Mol. Biol..

[14] Sun Y., Roznowski A.P., Tokuda J.M., Klose T., Mauney A., Pollack L., Fane B.A., Rossmann M.G. (2017). Structural changes of tailless bacteriophage PhiX174 during penetration of bacterial cell walls. Proc. Natl. Acad. Sci. USA.

[15] Sun L., Young L.N., Zhang X., Boudko S.P., Fokine A., Zbornik E., Roznowski A.P., Molineux I.J., Rossmann M.G., Fane B.A. (2014). Icosahedral bacteriophage PhiX174 forms a tail for DNA transport during infection. Nature.

[16] Cherwa J.E., Organtini L.J., Ashley R.E., Hafenstein S.L., Fane B.A. (2011). In Vitro Assembly of the oX174 Procapsid from External Scaffolding Protein Oligomers and Early Pentameric Assembly Intermediates. J. Mol. Biol..

[17] Dokland T., Bernal R.A., Burch A., Pletnev S., Fane B.A., Rossmann M.G. (1999). The role of scaffolding proteins in the assembly of the small, single-stranded DNA virus phiX174. J. Mol. Biol..

[18] Dokland T., McKenna R., Ilag L.L., Bowman B.R., Incardona N.L., Fane B.A., Rossmann M.G. (1997). Structure of a viral procapsid with molecular scaffolding. Nature.

[19] Ilag L.L., Olson N.H., Dokland T., Music C.L., Cheng R.H., Bowen Z., McKenna R., Rossmann M.G., Baker T.S., Incardona N.L. (1995). DNA packaging intermediates of bacteriophage phi X174. Structure.

[20] Novak C.R., Fane B.A. (2004). The functions of the N terminus of the phiX174 internal scaffolding protein, a protein encoded in an overlapping reading frame in a two scaffolding protein system. J. Mol. Biol..

[21] Azuma J., Morita J., Komano T. (1980). Process of attachment of phi X174 parental DNA to the host cell membrane. J. Biochem..

[22] Mano Y., Sakai H., Komano T. (1979). Growth and DNA synthesis of bacteriophage phi x174 in a dnaP mutant of Escherichia coli. J. Virol..

[23] Murakami Y., Nagata T., Schwarz W., Wada C., Yura T. (1985). Novel dnaG mutation in a dnaP mutant of Escherichia coli. J. Bacteriol..

[24] Shepard W., Cruse W.B., Fourme R., de la Fortelle E., Prange T. (1998). A zipper-like duplex in DNA: The crystal structure of d(GCGAAAGCT) at 2.1 A resolution. Structure.

[25] Luscombe N.M., Laskowski R.A., Thornton J.M. (2001). Amino acid-base interactions: A three-dimensional analysis of protein-DNA interactions at an atomic level. Nucleic Acids Res..

[26] Luscombe N.M., Thornton J.M. (2002). Protein-DNA interactions: Amino acid conservation and the effects of mutations on binding specificity. J. Mol. Biol..

[27] Mandel-Gutfreund Y., Schueler O., Margalit H. (1995). Comprehensive analysis of hydrogen bonds in regulatory protein DNA-complexes: In search of common principles. J. Mol. Biol..

[28] Suzuki M. (1994). A framework for the DNA-protein recognition code of the probe helix in transcription factors: The chemical and stereochemical rules. Structure.

[29] Conley M.J., McElwee M., Azmi L., Gabrielsen M., Byron O., Goodfellow I.G., Bhella D. (2019). Calicivirus VP2 forms a portal-like assembly following receptor engagement. Nature.

[30] Fane B.A., Hayashi M. (1991). Second-site suppressors of a cold-sensitive prohead accessory protein of bacteriophage phi X174. Genetics.

[31] Fane B.A., Head S., Hayashi M. (1992). Functional relationship between the J proteins of bacteriophages phi X174 and G4 during phage morphogenesis. J. Bacteriol..

[32] Roznowski A.P., Fane B.A. (2016). Structure-Function Analysis of the varphiX174 DNA-Piloting Protein Using Length-Altering Mutations. J. Virol..

[33] Burch A.D., Ta J., Fane B.A. (1999). Cross-functional analysis of the Microviridae internal scaffolding protein. J. Mol. Biol..

[34] Gordon E.B., Knuff C.J., Fane B.A. (2012). Conformational Switch-Defective øX174 Internal Scaffolding Proteins Kinetically Trap Assembly Intermediates before Procapsid Formation. J. Virol..

[35] Uchiyama A., Fane B.A. (2005). Identification of an interacting coat-external scaffolding protein domain required for both the initiation of phiX174 procapsid morphogenesis and the completion of DNA packaging. J. Virol..

[36] Cherwa J.E., Sanchez-Soria P., Wichman H.A., Fane B.A. (2009). Viral adaptation to an antiviral protein enhances the fitness level to above that of the uninhibited wild type. J. Virol..

[37] Hafenstein S.L., Chen M., Fane B.A. (2004). Genetic and functional analyses of the oX174 DNA binding protein: The effects of substitutions for amino acid residues that spatially organize the two DNA binding domains. Virology.

[38] Roznowski A.P., Young R.J., Love S.D., Andromita A.A., Guzman V.A., Wilch M.H., Block A., McGill A., Lavelle M., Romanova A. (2019). Recessive Host Range Mutants and Unsusceptible Cells That Inactivate Virions without Genome Penetration: Ecological and Technical Implications. J. Virol..

[39] Morein S., Henricson D., Rilfors L. (1994). Separation of inner and outer membrane vesicles from Escherichia coli in self-generating Percoll gradients. Anal. Biochem..

[40] Karkhanis Y.D., Zeltner J.Y., Jackson J.J., Carlo D.J. (1978). A new and improved microassay to determine 2-keto-3-deoxyoctonate in lipopolysaccharide of Gram-negative bacteria. Anal. Biochem..

[41] Rokyta D.R., Burch C.L., Caudle S.B., Wichman H.A. (2006). Horizontal gene transfer and the evolution of microvirid coliphage genomes. J. Bacteriol..

[42] Simossis V.A., Heringa J. (2005). PRALINE: A multiple sequence alignment toolbox that integrates homology-extended and secondary structure information. Nucleic Acids Res..

[43] Simossis V.A., Kleinjung J., Heringa J. (2005). Homology-extended sequence alignment. Nucleic Acids Res..

[44] Young L.N., Hockenberry A.M., Fane B.A. (2014). Mutations in the N terminus of the oX174 DNA pilot protein H confer defects in both assembly and host cell attachment. J. Virol..

[45] Ruboyianes M.V., Chen M., Dubrava M.S., Cherwa J.E., Fane B.A. (2009). The expression of N-terminal deletion DNA pilot proteins inhibits the early stages of phiX174 replication. J. Virol..

[46] Ilag L.L., McKenna R., Yadav M.P., BeMiller J.N., Incardona N.L., Rossmann M.G. (1994). Calcium ion-induced structural changes in bacteriophage phi X174. J. Mol. Biol..

[47] Boulanger P., Letellier L. (1992). Ion channels are likely to be involved in the two steps of phage T5 DNA penetration into Escherichia coli cells. J. Biol. Chem..

[48] Keweloh H.W., Bakker E.P. (1984). Increased permeability and subsequent resealing of the host cell membrane early after infection of Escherichia coli with bacteriophage T1. J. Bacteriol..

[49] Luria S.E., Delbruck M. (1943). Mutations of Bacteria from Virus Sensitivity to Virus Resistance. Genetics.

[50] Fane B.A., Shien S., Hayashi M. (1993). Second-site suppressors of a cold-sensitive external scaffolding protein of bacteriophage phi X174. Genetics.

[51] Gordon E.B., Fane B.A. (2013). The effects of an early conformational switch defect during øX174 morphogenesis are belatedly manifested late in the assembly pathway. J. Virol..

[52] Floor E. (1970). Interaction of morphogenetic genes of bacteriophage T4. J. Mol. Biol..

[53] Sternberg N. (1976). A genetic analysis of bacteriophage lambda head assembly. Virology.

[54] Van Valen D., Wu D., Chen Y.J., Tuson H., Wiggins P., Phillips R. (2012). A single-molecule Hershey-Chase experiment. Curr. Biol..

[55] Garcia L.R., Molineux I.J. (1995). Rate of translocation of bacteriophage T7 DNA across the membranes of Escherichia coli. J. Bacteriol..

[56] Lanni Y.T. (1968). First-step-transfer deoxyribonucleic acid of bacteriophage T5. Bacteriol. Rev..

[57] Lanni Y.T. (1965). DNA transfer from phage T5 to host cells: Dependence on intercurrent protein synthesis. Proc. Natl. Acad. Sci. USA.

[58] Moffatt B.A., Studier F.W. (1988). Entry of bacteriophage T7 DNA into the cell and escape from host restriction. J. Bacteriol..

