# Supplementary material for: Mutagenic Analysis of a DNA Translocating Tube’s Interior Surface
Source: Viruses. 2020 Jun 22;12(6):670. doi: 10.3390/v12060670 (PMC7354561; doi:10.3390/v12060670)
Supplement: Supplementary file 1 [file viruses-12-00670-s001.pdf]

Aaron Roznowski's K+ Efflux Assay

Julia Fisher
<julia@statlab.bio5.org>
Ed Bedrick
<edwardjbedrick@email.arizona.edu>

February 6, 2018

Contents

1 Experimental Purpose and Design **2**

2 Raw Data Plot **6**

3 Modeling **7**
3.1 Full Model . . . 7
3.2 Basic Model . . . 9

4 Appendix: R Version, Data Processing, Functions for Data Processing, Raw Output, and Additional Model Checking **11**
4.1 Versions . . . 11
4.2 Citation for R Packages (Libraries) Used . . . 11
4.3 Datasets . . . 21
4.4 Raw Model Output . . . 22
4.4.1 Model with Four Random Effects and Variance and Correlation Structures . . . 22
4.4.2 Model with Two Random Effects (no Variance or Correlation Structures) . . . 26
4.4.3 Model with Four Random Effects (no Variance or Correlation Structures) . . . 29
4.4.4 Model with Four Random Effects and a Correlation Structure (no Variance Structure) . . . 33
4.5 Model Diagnostic Plots . . . 38
4.5.1 Model with Four Random Effects and Variance and Correlation Structures . . . 38
4.5.2 Model with Two Random Effects (no Variance or Correlation Structures) . . . 40
4.5.3 Model with Four Random Effects (no Variance or Correlation Structures) . . . 42
4.5.4 Model with Four Random Effects and a Correlation Structure (no Variance Structure) . . . 44

4.6 Code 46

1 Experimental Purpose and Design

In order for a virus to infect an E.coli bacterium, it needs to transfer its genome into the E.coli. It accomplishes this by using protein H to form a tube between the virus and the E.coli through which the genome can travel. The inside of this tube is coated with the amino acid glutamine with the exception of one section. This section is composed of four rings. One of those rings is made of the amino acide lysine (K254), another is made of the amino acid methionine (M251), and a third is made of the amino acid threonine (T244). The fourth ring is also not glutamine, but Aaron did not mention its specific makeup.

When the tubes form, potassium leaks out of the E.coli bacteria. Thus, one byproduct of tube formation (and possibly infection) is an increase in potassium in the extracellular media surrounding the viruses and bacteria. In order to begin understanding the role these non-glutamine rings play in the infection process, Aaron has systematically changed one to two of the three named amino acid rings to glutamine. The outcome he is examining is the amount of extracellular potassium in the media surrounding the cells.

The study design is as follows:

1. The day before a run of the experiment, Aaron Roznowski makes a batch of buffer solution. The same buffer solution is used in all parts of a given day's run, but a new batch is used per run.
2. The day of the experiment, he grows a fresh batch of E.coli bacteria from a stock culture. He tries to grow the culture to 10^7 cells/mL (as measured by absorbance). However, there will be variation in how close he gets to this concentration. Because a single batch of E.coli is used for a run, all the samples from a given day will have the same E.coli concentration.
3. After the E.coli bacteria have been grown to the requisite concentration, Aaron washes the E.coli cells to remove the growth media.
4. Following this, a fixed number (measured in mL) of E.coli cells are placed into each of six test tubes.
5. In five of the six test tubes, a fixed number of viruses are added to the E.coli. Four of the five days of data used the same batch of viruses. One day (Nov. 7) used a different batch. Aaron tries to add 75 viruses per bacterium. One tube is left without viruses. The viruses are added to the bacteria at a low temperature (on ice). At this temperature, the viruses will attach to the bacteria but will not start building infection tubes.
6. Next, buffer is added to each test tube. The amount of added buffer changes per test tube since Aaron has decided to fix the total volume (E.coli + viruses + buffer) in each.
7. The viruses in the five test tubes are all different. In one of the tubes, the viruses are unmodified (the wildtype condition). In the other test tubes, the viruses have been modified so that particular rings of the infection tubes they form will be glutamine instead of the original amino acid. In one condition, the viruses will make tubes where the lysine (K254) ring has been changed to glutamine. In another, the methionine (M251) ring will be glutamine. In the third, the threonine (T244) ring will be glutamine. In the fourth condition, both the methionine and the threonine (M251 + T244) rings be glutamine.
8. Next, Aaron splits each of the six test tubes into two separate test tubes. One set of six will be measured at 37 degrees. The other will be measured at 22 degrees. At 37 degrees (the permissive condition), all the viruses should be able to build infection tubes and try to infect the E.coli. At 22 degrees (the restrictive condition), the wildtype virus should be able to infect the E.coli, but the mutated viruses dont 'work'.

9. Aaron always runs the 37 degree condition first. To do this, he places one test tube of the uninfected cells in a 37 degree water bath. He inserts a potassium-selective electrode into the tube. The electrode takes about a minute to give a steady-state potassium reading. After the reading, he wipes down the electrode with a chemical wipe, places the next test tube (wildtype) into the water bath, and inserts the electrode into that test tube. He continues this process with the remaining test tubes (T244, M251, K254, then T244 + M251). Then, he returns to the first test tube and repeats the process. He gathers multiple potassium measurements (up to about 25 minutes) from each test tube at 37 degrees. During this time, the other six test tubes remain on ice. After the measurements for the 37 degree condition are done, he repeats the process but with a 22 degree water bath. Note that the 22 degree condition gets more time measurements than the 37 degree condition because it takes longer in this condition to see any differences.

There are some caveats that must be made to the inference in the study. First, because the 37 degree condition always occurs before the 22 degree condition, temperature batch order is confounded with temperature. Similarly, because the virus conditions are always started in the water baths in the same order, the virus condition is also confounded with the virus 'starting' order. Thus, technically, any differences between virus conditions could in fact be due to starting order.

The primary fixed predictors of potassium concentration are virus condition (uninfected, wildtype, T244, M251, K254, and T244 + M251), time (continuous), and water bath temperature (22 or 37 degrees). Individual trajectories for each virus by temperature condition were desired, and thus we planned to include all interactions of the three predictors in the model. However, an initial plot of the data (shown in the next section) revealed a clear nonlinear relationship between potassium concentration and time. Thus, we slightly modified the fixed-effects structure of the model as follows: we centered the predictor time and fit a model with all three-way interactions among virus, centered time, and temperature *and* all three-way interactions among virus condition, centered time squared, and temperature. This adjustment allows the model to capture a parabolic trajectory of potassium concentration with time for each virus by temperature condition.

It was clear from the experimental design that we needed to account for random sources of variation in the model. From the design, we indentified four such reasonable sources: virus batch, day, original test tube (before splitting in half), and final test tube. Initially, we fit a mixed effects model to the potassium concentration data. It included the fixed effects structure described in the previous paragragh and these four nested random effects. However, because the data are gathered over time, we felt it was likely that there would be an additional correlation between close time points (with that correlation possibly decreasing over time). After some investigation into the relationship of points separated over time using this first mixed effects model, we modified the model to include a decreasing exponential correlation structure to the data. After fitting this second model, it became apparent that there was additional evidence of heteroscedasticity over time. Specifically, there was more variation in the data at earlier time points. To account for this, we updated the second model to include an exponential variance structure. This final model is written formally below:

$$
\begin{aligned}
Y_{ijklm} = \mu &+ \text{virus batch}_i + \text{day}_{ij} + \text{virus condition}_k \\
&+ \text{original test tube}_{ijk} + \text{temp}_l + (\text{virus condition} : \text{temp})_{kl} \\
&+ \text{final test tube}_{ijkl} + \gamma_{\text{linear}}(x_{ijklm} - \bar{x}_{.....}) + \gamma_{\text{quadratic}}(x_{ijklm} - \bar{x}_{.....})^2 \\
&+ (\gamma_{\text{linear}} : \text{virus condition})(x_{ijklm} - \bar{x}_{.....}) + (\gamma_{\text{quadratic}} : \text{virus condition})(x_{ijklm} - \bar{x}_{.....})^2 \\
&+ (\gamma_{\text{linear}} : \text{temp})(x_{ijklm} - \bar{x}_{.....}) + (\gamma_{\text{quadratic}} : \text{temp})(x_{ijklm} - \bar{x}_{.....})^2 \\
&+ (\gamma_{\text{linear}} : \text{virus condition} : \text{temp})(x_{ijklm} - \bar{x}_{.....}) + (\gamma_{\text{quadratic}} : \text{virus condition} : \text{temp})(x_{ijklm} - \bar{x}_{.....})^2 \\
&+ \epsilon_{ijklm}
\end{aligned}
$$

where...

- $i \in \{1, 2\}$
- $j = 1$ or $j \in \{1, 2, 3, 4\}$
- $k \in \{1, 2, ..., 6\}$
- $l \in \{1, 2\}$
- $m \in \{1, 2, ..., 5\}$ or $m \in \{1, 2, ..., 16\}$
- virus $\text{batch}_i \sim N(0, \sigma^2_{\text{vb}})$
- $\text{day}_{ij} \sim N(0, \sigma^2_{\text{day}})$
- original test $\text{tube}_{ijk} \sim N(0, \sigma^2_{\text{ott}})$
- final test $\text{tube}_{ijkl} \sim N(0, \sigma^2_{\text{ftt}})$
- $\epsilon_{ijkl} \sim N(0, \sigma^2 \boldsymbol{\Lambda}_{ijkl})$
- $\boldsymbol{\Lambda}_{ijkl} = \boldsymbol{V}_{ijkl} \boldsymbol{C}_{ijkl} \boldsymbol{V}_{ijkl}$
- $\text{Var}(\epsilon_{ijklm} | \text{virus batch}_i, \text{day}_{ij}, \text{original test tube}_{ijk}, \text{final test tube}_{ijkl}) = \sigma^2 [\boldsymbol{V}_{ijkl}]^2_{mm}$ and $[\boldsymbol{V}_{ijkl}]_{mm} = \exp(\delta \cdot \text{time}_{ijklm})$ where δ is estimated from the data.
- Let s be the difference in minutes between the observations with errors ϵ_{ijklm} and $\epsilon_{ijklm'}$. Then,

$$
[\boldsymbol{C}_{ijkl}]_{mm'} = \text{cor}(\epsilon_{ijklm}, \epsilon_{ijklm'} | \text{virus batch}_i, \text{day}_{ij}, \text{original test tube}_{ijk}, \text{final test tube}_{ijkl}) = \begin{cases} (1 - c_0) \exp(-s/\rho) \ , & s > 0 \\ 1 \ , & s = 0 \end{cases}
$$

The values of c_0 and ρ are estimated from the data.

Because this final model is quite complex, we additionally fit a model with the same fixed effects structure but no correlation or variance structure and only two random effects — day and final test tube. The contrasts of primary interest (mutated virus conditions versus both wildtype and uninfected at 25 minutes in the 37 degree condition and at 150 minutes in the 22 degree condition) were highly similar for the two analyses. While the estimates and p-values varied a bit, the inference did not change between the analyses. Thus, while we feel the more complex model is more faithful to the random, variational, and correlation structure of the data, the less complex model is sufficient. Results could be reasonably reported from either model with a footnote that the other model was also fit and results did not change.

It is worth noting for future experiments that you might consider the following in order to simplify the modeling and inference a bit. (Of course, some of these may not be feasible!)

- Use either the same virus batch for all runs or a different virus batch for each run. This would eliminate the need for a "virus batch" random effect. In the first case, the effect would disappear since there wouldn't be any differences among virus batches. In the second case, the virus batch effect would get combined with the "day" random effect.
- When preparing the flasks initially, prepare twelve instead of six flasks. In other words, you could get rid of the "original test tube" random effect if you never split the six test tubes but rather started with twelve.
- Randomize the temperature order. Doing this will allow separation between any effect of temperature order and temperature.
- Randomize the order in which you place flasks into the water baths. Doing this will allow separation between any effect of test tube placement order and virus condition.
- If possible, keep your timepoints the same time apart. This will open up more options for correlation structures.

2 Raw Data Plot

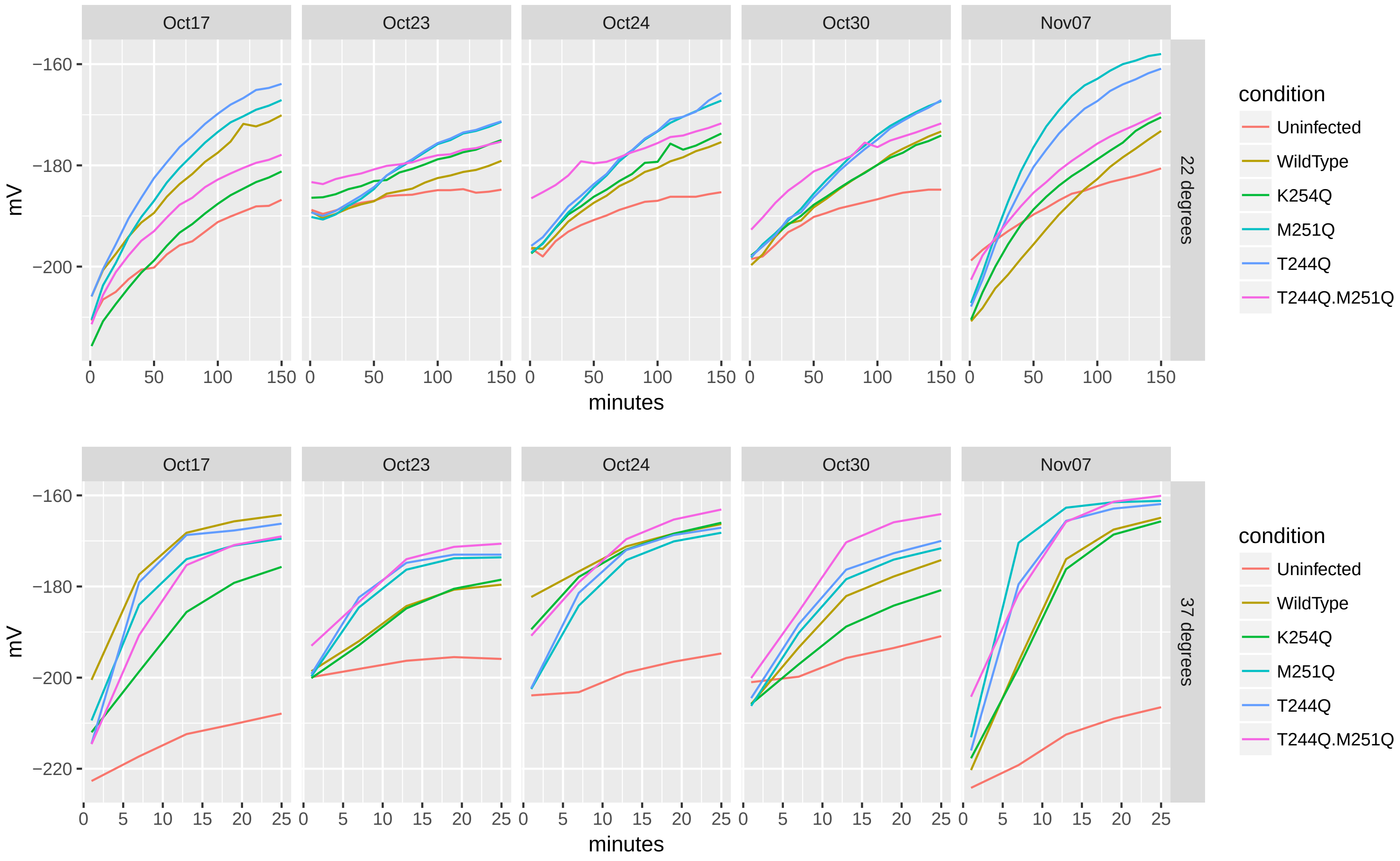

3 Modeling

The estimated model parameters and model diagnostic plots are included in the appendix. Below, we present plots of the model-estimated values with pointwise 95% confidence intervals and estimated differences between each of the mutated virus conditions and wildtype and between each of the mutated virus conditions and uninfected at 22 degrees, 150 minutes and 37 degrees, 25 minutes.

3.1 Full Model

This model is the full model described in the introduction. It has four levels of nested random effects and both a variance and a correlation structure.

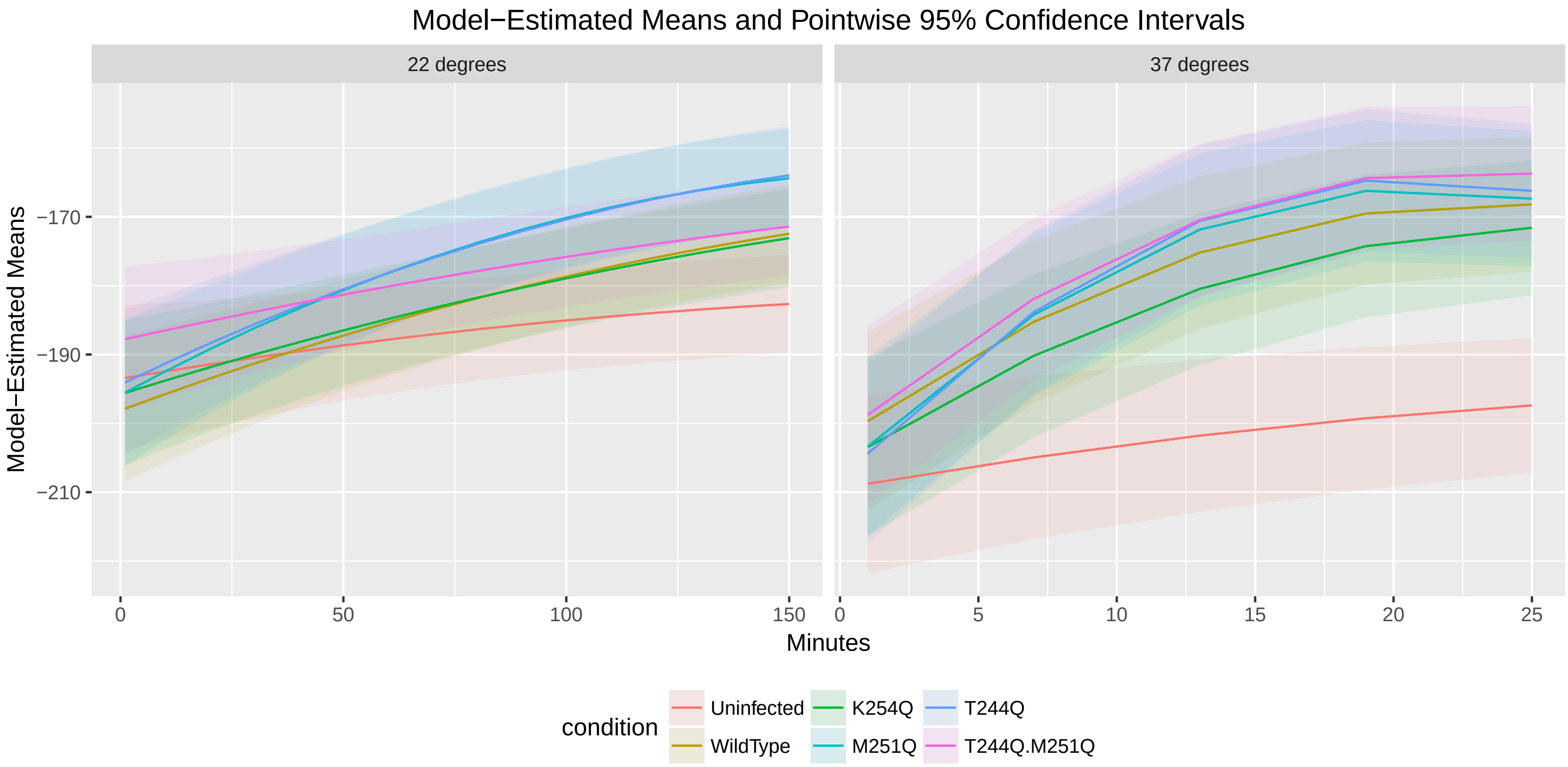

Contrast	Estimated Difference	SE	df	T	P-Value	95% CI
K254Q vs. WildType at 25 minutes in 37 degrees	-3.40	4.89	20	-0.70	0.49	(-13.59, 6.8)
M251Q vs. WildType at 25 minutes in 37 degrees	0.84	4.89	20	0.17	0.87	(-9.36, 11.03)
T244Q vs. WildType at 25 minutes in 37 degrees	1.96	4.89	20	0.40	0.69	(-8.23, 12.16)
T244Q.M251Q vs. WildType at 25 minutes in 37 degrees	4.47	4.89	20	0.92	0.37	(-5.72, 14.67)
K254Q vs. Uninfected at 25 minutes in 37 degrees	25.81	4.89	20	5.28	0.00	(15.62, 36)
M251Q vs. Uninfected at 25 minutes in 37 degrees	30.04	4.89	20	6.15	0.00	(19.85, 40.24)
T244Q vs. Uninfected at 25 minutes in 37 degrees	31.17	4.89	20	6.38	0.00	(20.98, 41.37)
T244Q.M251Q vs. Uninfected at 25 minutes in 37 degrees	33.68	4.89	20	6.89	0.00	(23.49, 43.87)
K254Q vs. WildType at 150 minutes in 22 degrees	-0.63	1.68	20	-0.38	0.71	(-4.14, 2.87)
M251Q vs. WildType at 150 minutes in 22 degrees	8.06	1.68	20	4.80	0.00	(4.55, 11.56)
T244Q vs. WildType at 150 minutes in 22 degrees	8.48	1.68	20	5.05	0.00	(4.98, 11.99)
T244Q.M251Q vs. WildType at 150 minutes in 22 degrees	1.04	1.68	20	0.62	0.54	(-2.46, 4.55)
K254Q vs. Uninfected at 150 minutes in 22 degrees	9.57	1.68	20	5.69	0.00	(6.06, 13.08)
M251Q vs. Uninfected at 150 minutes in 22 degrees	18.26	1.68	20	10.87	0.00	(14.76, 21.77)
T244Q vs. Uninfected at 150 minutes in 22 degrees	18.69	1.68	20	11.12	0.00	(15.18, 22.19)
T244Q.M251Q vs. Uninfected at 150 minutes in 22 degrees	11.25	1.68	20	6.69	0.00	(7.74, 14.75)

3.2 Basic Model

This model is the basic model described in the introduction. It has two levels of nested random effects but additional no variance no correlation structure.

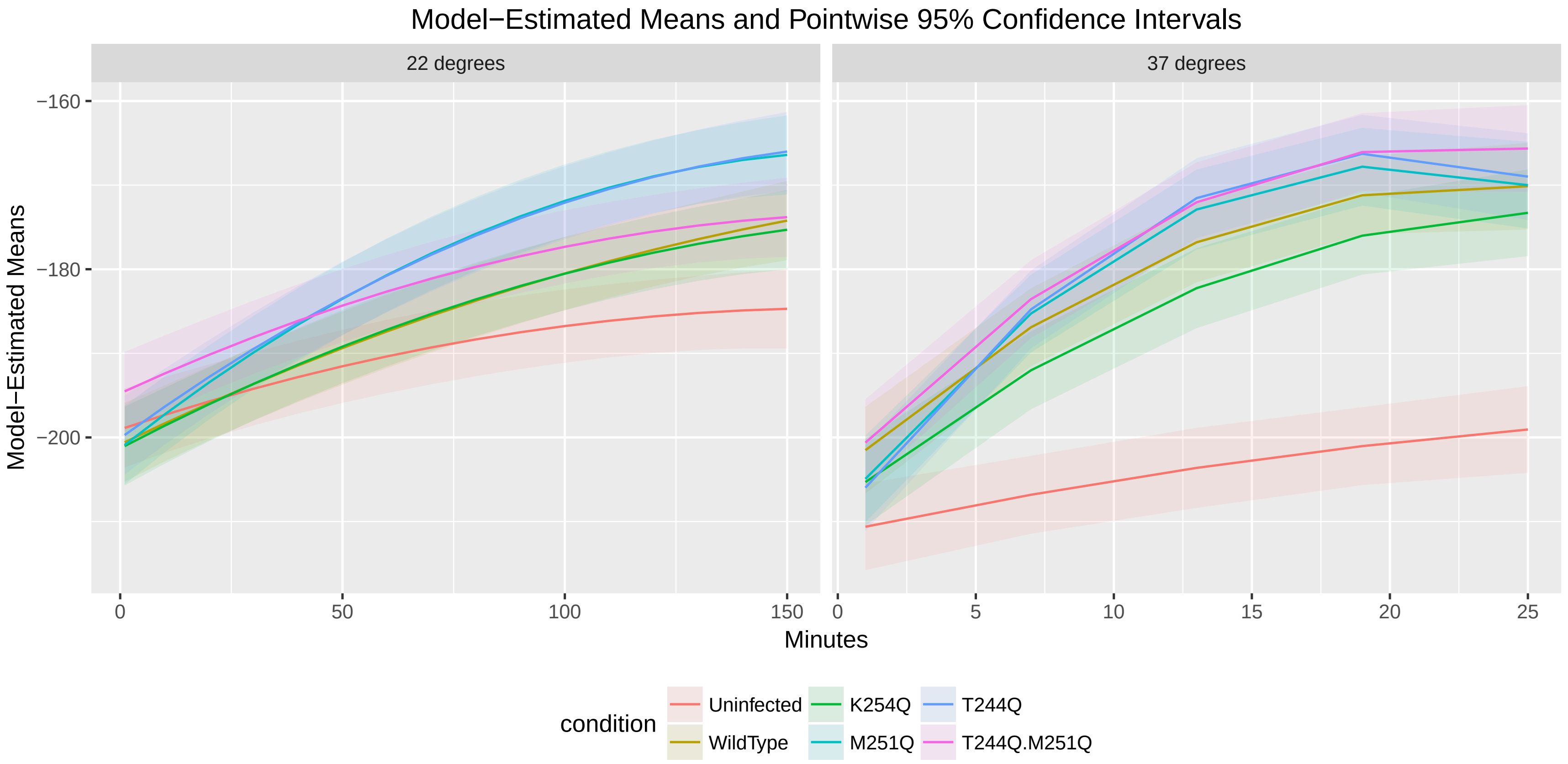

Contrast	Estimated Difference	SE	df	T	P-Value	95% CI
K254Q vs. WildType at 25 minutes in 37 degrees	-3.17	3.41	44	-0.93	0.36	(-10.04, 3.71)
M251Q vs. WildType at 25 minutes in 37 degrees	0.12	3.41	44	0.04	0.97	(-6.75, 7)
T244Q vs. WildType at 25 minutes in 37 degrees	1.14	3.41	44	0.33	0.74	(-5.74, 8.02)
T244Q.M251Q vs. WildType at 25 minutes in 37 degrees	4.48	3.41	44	1.31	0.20	(-2.4, 11.36)
K254Q vs. Uninfected at 25 minutes in 37 degrees	25.76	3.41	44	7.55	0.00	(18.88, 32.64)
M251Q vs. Uninfected at 25 minutes in 37 degrees	29.05	3.41	44	8.51	0.00	(22.18, 35.93)
T244Q vs. Uninfected at 25 minutes in 37 degrees	30.07	3.41	44	8.81	0.00	(23.19, 36.95)
T244Q.M251Q vs. Uninfected at 25 minutes in 37 degrees	33.41	3.41	44	9.79	0.00	(26.53, 40.28)
K254Q vs. WildType at 150 minutes in 22 degrees	-1.08	3.08	44	-0.35	0.73	(-7.29, 5.12)
M251Q vs. WildType at 150 minutes in 22 degrees	7.81	3.08	44	2.54	0.01	(1.61, 14.02)
T244Q vs. WildType at 150 minutes in 22 degrees	8.21	3.08	44	2.67	0.01	(2, 14.41)
T244Q.M251Q vs. WildType at 150 minutes in 22 degrees	0.41	3.08	44	0.13	0.89	(-5.79, 6.62)
K254Q vs. Uninfected at 150 minutes in 22 degrees	9.40	3.08	44	3.05	0.00	(3.2, 15.61)
M251Q vs. Uninfected at 150 minutes in 22 degrees	18.30	3.08	44	5.94	0.00	(12.09, 24.5)
T244Q vs. Uninfected at 150 minutes in 22 degrees	18.70	3.08	44	6.07	0.00	(12.49, 24.9)
T244Q.M251Q vs. Uninfected at 150 minutes in 22 degrees	10.90	3.08	44	3.54	0.00	(4.69, 17.1)

4 Appendix: R Version, Data Processing, Functions for Data Processing, Raw Output, and Additional Model Checking

4.1 Versions

The following is the session information for this analysis. This includes the version of R and versions of all packages used.

```
R version 3.4.3 (2017-11-30)
Platform: x86_64-apple-darwin15.6.0 (64-bit)
Running under: macOS High Sierra 10.13.3

Matrix products: default
BLAS: /Library/Frameworks/R.framework/Versions/3.4/Resources/lib/libRblas.0.dylib
LAPACK: /Library/Frameworks/R.framework/Versions/3.4/Resources/lib/libRlapack.dylib

locale:
[1] en_US.UTF-8/en_US.UTF-8/en_US.UTF-8/C/en_US.UTF-8/en_US.UTF-8

attached base packages:
[1] stats     graphics  grDevices utils     datasets  methods   base

other attached packages:
 [1] bindrcpp_0.2    nlme_3.1-131    xlsx_0.5.7      xlsxjars_0.6.1  rJava_0.9-9     xtable_1.8-2    pbkrtest_0.4-7  car_2.1-6       lsmeans_2.27-61
[10] lme4_1.1-15     Matrix_1.2-12   ggplot2_2.2.1   dplyr_0.7.4     tidyr_0.8.0     knitr_1.19

loaded via a namespace (and not attached):
 [1] zoo_1.8-1          tidyselect_0.2.3   reshape2_1.4.3     purrr_0.2.4        splines_3.4.3      lattice_0.20-35    colorspace_1.3-2
 [8] mgcv_1.8-22        survival_2.41-3    rlang_0.1.6        nloptr_1.0.4       pillar_1.1.0       glue_1.2.0         multcomp_1.4-8
[15] bindr_0.1          plyr_1.8.4         stringr_1.2.0      MatrixModels_0.4-1 munsell_0.4.3      gtable_0.2.0       mvtnorm_1.0-7
[22] codetools_0.2-15   coda_0.19-1        evaluate_0.10.1    labeling_0.3       SparseM_1.77       quantreg_5.34      parallel_3.4.3
[29] TH.data_1.0-8      Rcpp_0.12.15       scales_0.5.0       digest_0.6.13      stringi_1.1.6      grid_3.4.3         tools_3.4.3
[36] sandwich_2.4-0     magrittr_1.5       lazyeval_0.2.1     tibble_1.4.2       pkgconfig_2.0.1    MASS_7.3-47        estimability_1.2
[43] assertthat_0.2.0   minqa_1.2.4        R6_2.2.2           nnet_7.3-12        compiler_3.4.3
```

4.2 Citation for R Packages (Libraries) Used

Here is citation information for all the R packages used.

```
======================================================================
package: bindrcpp
----------------------------------------------------------------------

To cite package 'bindrcpp' in publications use:
```

```
  Kirill Mller (2017). bindrcpp: An 'Rcpp' Interface to Active Bindings. R package version 0.2.
  https://CRAN.R-project.org/package=bindrcpp

A BibTeX entry for LaTeX users is

  @Manual{,
    title = {bindrcpp: An 'Rcpp' Interface to Active Bindings},
    author = {Kirill Mller},
    year = {2017},
    note = {R package version 0.2},
    url = {https://CRAN.R-project.org/package=bindrcpp},
  }


======================================================================
package: nlme
----------------------------------------------------------------------

Pinheiro J, Bates D, DebRoy S, Sarkar D and R Core Team (2017). _nlme: Linear and Nonlinear Mixed Effects Models_. R package version
3.1-131, <URL: https://CRAN.R-project.org/package=nlme>.

A BibTeX entry for LaTeX users is

  @Manual{,
    title = {{nlme}: Linear and Nonlinear Mixed Effects Models},
    author = {Jose Pinheiro and Douglas Bates and Saikat DebRoy and Deepayan Sarkar and {R Core Team}},
    year = {2017},
    note = {R package version 3.1-131},
    url = {https://CRAN.R-project.org/package=nlme},
  }


======================================================================
package: xlsx
----------------------------------------------------------------------

To cite package 'xlsx' in publications use:

  Adrian A. Dragulescu (2014). xlsx: Read, write, format Excel 2007 and Excel 97/2000/XP/2003 files. R package version 0.5.7.
  https://CRAN.R-project.org/package=xlsx

A BibTeX entry for LaTeX users is

  @Manual{,
    title = {xlsx: Read, write, format Excel 2007 and Excel 97/2000/XP/2003 files},
    author = {Adrian A. Dragulescu},
    year = {2014},
    note = {R package version 0.5.7},
```

```
    url = {https://CRAN.R-project.org/package=xlsx},
  }

ATTENTION: This citation information has been auto-generated from the package DESCRIPTION file and may need manual editing, see
'help("citation")'.


======================================================================
package: xlsxjars
----------------------------------------------------------------------

To cite package 'xlsxjars' in publications use:

  Adrian A. Dragulescu (2014). xlsxjars: Package required POI jars for the xlsx package. R package version 0.6.1.
  https://CRAN.R-project.org/package=xlsxjars

A BibTeX entry for LaTeX users is

  @Manual{,
    title = {xlsxjars: Package required POI jars for the xlsx package},
    author = {Adrian A. Dragulescu},
    year = {2014},
    note = {R package version 0.6.1},
    url = {https://CRAN.R-project.org/package=xlsxjars},
  }


======================================================================
package: rJava
----------------------------------------------------------------------

To cite package 'rJava' in publications use:

  Simon Urbanek (2017). rJava: Low-Level R to Java Interface. R package version 0.9-9. https://CRAN.R-project.org/package=rJava

A BibTeX entry for LaTeX users is

  @Manual{,
    title = {rJava: Low-Level R to Java Interface},
    author = {Simon Urbanek},
    year = {2017},
    note = {R package version 0.9-9},
    url = {https://CRAN.R-project.org/package=rJava},
  }

ATTENTION: This citation information has been auto-generated from the package DESCRIPTION file and may need manual editing, see
'help("citation")'.
```

```
======================================================================
package: xtable
----------------------------------------------------------------------

To cite package 'xtable' in publications use:

  David B. Dahl (2016). xtable: Export Tables to LaTeX or HTML. R package version 1.8-2. https://CRAN.R-project.org/package=xtable

A BibTeX entry for LaTeX users is

  @Manual{,
    title = {xtable: Export Tables to LaTeX or HTML},
    author = {David B. Dahl},
    year = {2016},
    note = {R package version 1.8-2},
    url = {https://CRAN.R-project.org/package=xtable},
  }

ATTENTION: This citation information has been auto-generated from the package DESCRIPTION file and may need manual editing, see
'help("citation")'.


======================================================================
package: pbkrtest
----------------------------------------------------------------------

To cite pbkrtest in publications use:

  Ulrich Halekoh, Sren Hjsgaard (2014). A Kenward-Roger Approximation and Parametric Bootstrap Methods for Tests in Linear Mixed
  Models - The R Package pbkrtest. Journal of Statistical Software, 59(9), 1-30. URL http://www.jstatsoft.org/v59/i09/.

A BibTeX entry for LaTeX users is

  @Article{,
    title = {A Kenward-Roger Approximation and Parametric Bootstrap Methods for Tests in Linear Mixed Models -- The {R} Package {pbkrtest}},
    author = {Ulrich Halekoh and S{\o}ren H{\o}jsgaard},
    journal = {Journal of Statistical Software},
    year = {2014},
    volume = {59},
    number = {9},
    pages = {1--30},
    url = {http://www.jstatsoft.org/v59/i09/},
  }


======================================================================
package: car
----------------------------------------------------------------------
```

```
To cite the car package in publications use:

  John Fox and Sanford Weisberg (2011). An {R} Companion to Applied Regression, Second Edition. Thousand Oaks CA: Sage. URL:
  http://socserv.socsci.mcmaster.ca/jfox/Books/Companion

A BibTeX entry for LaTeX users is

  @Book{,
    title = {An {R} Companion to Applied Regression},
    edition = {Second},
    author = {John Fox and Sanford Weisberg},
    year = {2011},
    publisher = {Sage},
    address = {Thousand Oaks {CA}},
    url = {http://socserv.socsci.mcmaster.ca/jfox/Books/Companion},
  }


======================================================================
package: lsmeans
----------------------------------------------------------------------

To cite lsmeans in publications use:

  Russell V. Lenth (2016). Least-Squares Means: The R Package lsmeans. Journal of Statistical Software, 69(1), 1-33.
  doi:10.18637/jss.v069.i01

A BibTeX entry for LaTeX users is

  @Article{,
    title = {Least-Squares Means: The {R} Package {lsmeans}},
    author = {Russell V. Lenth},
    journal = {Journal of Statistical Software},
    year = {2016},
    volume = {69},
    number = {1},
    pages = {1--33},
    doi = {10.18637/jss.v069.i01},
  }


======================================================================
package: lme4
----------------------------------------------------------------------

To cite lme4 in publications use:

  Douglas Bates, Martin Maechler, Ben Bolker, Steve Walker (2015). Fitting Linear Mixed-Effects Models Using lme4. Journal of
```

```
  Statistical Software, 67(1), 1-48. doi:10.18637/jss.v067.i01.

A BibTeX entry for LaTeX users is

  @Article{,
    title = {Fitting Linear Mixed-Effects Models Using {lme4}},
    author = {Douglas Bates and Martin M{\"a}chler and Ben Bolker and Steve Walker},
    journal = {Journal of Statistical Software},
    year = {2015},
    volume = {67},
    number = {1},
    pages = {1--48},
    doi = {10.18637/jss.v067.i01},
  }


======================================================================
package: Matrix
----------------------------------------------------------------------

To cite package 'Matrix' in publications use:

  Douglas Bates and Martin Maechler (2017). Matrix: Sparse and Dense Matrix Classes and Methods. R package version 1.2-12.
  https://CRAN.R-project.org/package=Matrix

A BibTeX entry for LaTeX users is

  @Manual{,
    title = {Matrix: Sparse and Dense Matrix Classes and Methods},
    author = {Douglas Bates and Martin Maechler},
    year = {2017},
    note = {R package version 1.2-12},
    url = {https://CRAN.R-project.org/package=Matrix},
  }


======================================================================
package: ggplot2
----------------------------------------------------------------------

To cite ggplot2 in publications, please use:

  H. Wickham. ggplot2: Elegant Graphics for Data Analysis. Springer-Verlag New York, 2009.

A BibTeX entry for LaTeX users is

  @Book{,
    author = {Hadley Wickham},
```

```
    title = {ggplot2: Elegant Graphics for Data Analysis},
    publisher = {Springer-Verlag New York},
    year = {2009},
    isbn = {978-0-387-98140-6},
    url = {http://ggplot2.org},
  }


======================================================================
package: dplyr
----------------------------------------------------------------------

To cite package 'dplyr' in publications use:

  Hadley Wickham, Romain Francois, Lionel Henry and Kirill Mller (2017). dplyr: A Grammar of Data Manipulation. R package version
  0.7.4. https://CRAN.R-project.org/package=dplyr

A BibTeX entry for LaTeX users is

  @Manual{,
    title = {dplyr: A Grammar of Data Manipulation},
    author = {Hadley Wickham and Romain Francois and Lionel Henry and Kirill Mller},
    year = {2017},
    note = {R package version 0.7.4},
    url = {https://CRAN.R-project.org/package=dplyr},
  }


======================================================================
package: tidyr
----------------------------------------------------------------------

To cite package 'tidyr' in publications use:

  Hadley Wickham and Lionel Henry (2018). tidyr: Easily Tidy Data with 'spread()' and 'gather()' Functions. R package version 0.8.0.
  https://CRAN.R-project.org/package=tidyr

A BibTeX entry for LaTeX users is

  @Manual{,
    title = {tidyr: Easily Tidy Data with 'spread()' and 'gather()' Functions},
    author = {Hadley Wickham and Lionel Henry},
    year = {2018},
    note = {R package version 0.8.0},
    url = {https://CRAN.R-project.org/package=tidyr},
  }
```

```
======================================================================
package: knitr
----------------------------------------------------------------------

To cite the 'knitr' package in publications use:

  Yihui Xie (2018). knitr: A General-Purpose Package for Dynamic Report Generation in R. R package version 1.19.

  Yihui Xie (2015) Dynamic Documents with R and knitr. 2nd edition. Chapman and Hall/CRC. ISBN 978-1498716963

  Yihui Xie (2014) knitr: A Comprehensive Tool for Reproducible Research in R. In Victoria Stodden, Friedrich Leisch and Roger D.
  Peng, editors, Implementing Reproducible Computational Research. Chapman and Hall/CRC. ISBN 978-1466561595

To see these entries in BibTeX format, use 'print(<citation>, bibtex=TRUE)', 'toBibtex(.)', or set 'options(citation.bibtex.max=999)'.


======================================================================
package: stats
----------------------------------------------------------------------

The 'stats' package is part of R.  To cite R in publications use:

  R Core Team (2017). R: A language and environment for statistical computing. R Foundation for Statistical Computing, Vienna,
  Austria. URL https://www.R-project.org/.

A BibTeX entry for LaTeX users is

  @Manual{,
    title = {R: A Language and Environment for Statistical Computing},
    author = {{R Core Team}},
    organization = {R Foundation for Statistical Computing},
    address = {Vienna, Austria},
    year = {2017},
    url = {https://www.R-project.org/},
  }

We have invested a lot of time and effort in creating R, please cite it when using it for data analysis. See also
'citation("pkgname")' for citing R packages.


======================================================================
package: graphics
----------------------------------------------------------------------

The 'graphics' package is part of R.  To cite R in publications use:

  R Core Team (2017). R: A language and environment for statistical computing. R Foundation for Statistical Computing, Vienna,
  Austria. URL https://www.R-project.org/.
```

```
A BibTeX entry for LaTeX users is

  @Manual{,
    title = {R: A Language and Environment for Statistical Computing},
    author = {{R Core Team}},
    organization = {R Foundation for Statistical Computing},
    address = {Vienna, Austria},
    year = {2017},
    url = {https://www.R-project.org/},
  }

We have invested a lot of time and effort in creating R, please cite it when using it for data analysis. See also
'citation("pkgname")' for citing R packages.


======================================================================
package: grDevices
----------------------------------------------------------------------

The 'grDevices' package is part of R.  To cite R in publications use:

  R Core Team (2017). R: A language and environment for statistical computing. R Foundation for Statistical Computing, Vienna,
  Austria. URL https://www.R-project.org/.

A BibTeX entry for LaTeX users is

  @Manual{,
    title = {R: A Language and Environment for Statistical Computing},
    author = {{R Core Team}},
    organization = {R Foundation for Statistical Computing},
    address = {Vienna, Austria},
    year = {2017},
    url = {https://www.R-project.org/},
  }

We have invested a lot of time and effort in creating R, please cite it when using it for data analysis. See also
'citation("pkgname")' for citing R packages.


======================================================================
package: utils
----------------------------------------------------------------------

The 'utils' package is part of R.  To cite R in publications use:

  R Core Team (2017). R: A language and environment for statistical computing. R Foundation for Statistical Computing, Vienna,
  Austria. URL https://www.R-project.org/.
```

```
A BibTeX entry for LaTeX users is

  @Manual{,
    title = {R: A Language and Environment for Statistical Computing},
    author = {{R Core Team}},
    organization = {R Foundation for Statistical Computing},
    address = {Vienna, Austria},
    year = {2017},
    url = {https://www.R-project.org/},
  }

We have invested a lot of time and effort in creating R, please cite it when using it for data analysis. See also
'citation("pkgname")' for citing R packages.


======================================================================
package: datasets
----------------------------------------------------------------------

The 'datasets' package is part of R.  To cite R in publications use:

  R Core Team (2017). R: A language and environment for statistical computing. R Foundation for Statistical Computing, Vienna,
  Austria. URL https://www.R-project.org/.

A BibTeX entry for LaTeX users is

  @Manual{,
    title = {R: A Language and Environment for Statistical Computing},
    author = {{R Core Team}},
    organization = {R Foundation for Statistical Computing},
    address = {Vienna, Austria},
    year = {2017},
    url = {https://www.R-project.org/},
  }

We have invested a lot of time and effort in creating R, please cite it when using it for data analysis. See also
'citation("pkgname")' for citing R packages.


======================================================================
package: methods
----------------------------------------------------------------------

The 'methods' package is part of R.  To cite R in publications use:

  R Core Team (2017). R: A language and environment for statistical computing. R Foundation for Statistical Computing, Vienna,
  Austria. URL https://www.R-project.org/.
```

```
A BibTeX entry for LaTeX users is

  @Manual{,
    title = {R: A Language and Environment for Statistical Computing},
    author = {{R Core Team}},
    organization = {R Foundation for Statistical Computing},
    address = {Vienna, Austria},
    year = {2017},
    url = {https://www.R-project.org/},
  }

We have invested a lot of time and effort in creating R, please cite it when using it for data analysis. See also
'citation("pkgname")' for citing R packages.


======================================================================
package: base
----------------------------------------------------------------------

To cite R in publications use:

  R Core Team (2017). R: A language and environment for statistical computing. R Foundation for Statistical Computing, Vienna,
  Austria. URL https://www.R-project.org/.

A BibTeX entry for LaTeX users is

  @Manual{,
    title = {R: A Language and Environment for Statistical Computing},
    author = {{R Core Team}},
    organization = {R Foundation for Statistical Computing},
    address = {Vienna, Austria},
    year = {2017},
    url = {https://www.R-project.org/},
  }

We have invested a lot of time and effort in creating R, please cite it when using it for data analysis. See also
'citation("pkgname")' for citing R packages.
```

4.3 Datasets

The following datasets were used in this document:

`/Users/jmfisher/Documents/Projects/Roznowski/KplusEffluxAssay/data/PhiX174RawPotassiumEfflux22Degrees.csv`

`/Users/jmfisher/Documents/Projects/Roznowski/KplusEffluxAssay/data/PhiX174RawPotassiumEfflux37Degrees.csv`

4.4 Raw Model Output

Summaries of the primary (complex) model, the simple comparison model, and the two intermediate-stage models are shown below.

4.4.1 Model with Four Random Effects and Variance and Correlation Structures

```
Linear mixed-effects model fit by REML
 Data: dta
       AIC      BIC    logLik
  2504.513 2697.535 -1208.256

Random effects:
 Formula: ~1 | virus_batch
        (Intercept)
StdDev:    4.397825

 Formula: ~1 | date %in% virus_batch
        (Intercept)
StdDev:    1.587116

 Formula: ~1 | test_tube %in% date %in% virus_batch
        (Intercept)
StdDev:    1.836431

 Formula: ~1 | unit %in% test_tube %in% date %in% virus_batch
        (Intercept) Residual
StdDev:    1.825281 11.97949

Correlation Structure: Exponential spatial correlation
 Formula: ~minutes_cent | virus_batch/date/test_tube/unit
 Parameter estimate(s):
       range       nugget
221.49428727   0.01685641
Variance function:
 Structure: Exponential of variance covariate
 Formula: ~minutes
 Parameter estimates:
      expon
-0.01990992
Fixed effects: mV ~ condition * degrees * minutes_cent + condition * degrees *      I(minutes_cent^2)
                                          Value Std.Error  DF   t-value p-value
(Intercept)                          -222.81510  4.494345 546 -49.57677  0.0000
condition1                             29.08176  6.949617  20   4.18466  0.0005
condition2                              5.72079  6.949617  20   0.82318  0.4201
condition3                             16.78634  6.949617  20   2.41543  0.0254
condition4                            -21.59415  6.949617  20  -3.10724  0.0056
condition5                            -28.22502  6.949617  20  -4.06138  0.0006
```

```
degrees1                                  40.44410  3.089825  24  13.08945  0.0000
minutes_cent                              -2.34992  0.137049 546 -17.14654  0.0000
I(minutes_cent^2)                         -0.03274  0.001492 546 -21.93486  0.0000
condition1:degrees1                      -34.53175  6.909059  24  -4.99804  0.0000
condition2:degrees1                       -8.64106  6.909059  24  -1.25069  0.2231
condition3:degrees1                      -19.21649  6.909059  24  -2.78135  0.0104
condition4:degrees1                       25.86200  6.909059  24   3.74320  0.0010
condition5:degrees1                       32.47790  6.909059  24   4.70077  0.0001
condition1:minutes_cent                    2.19787  0.306451 546   7.17203  0.0000
condition2:minutes_cent                    0.22750  0.306451 546   0.74236  0.4582
condition3:minutes_cent                    0.77736  0.306451 546   2.53666  0.0115
condition4:minutes_cent                   -1.21819  0.306451 546  -3.97516  0.0001
condition5:minutes_cent                   -1.57651  0.306451 546  -5.14441  0.0000
degrees1:minutes_cent                      2.51749  0.137049 546  18.36927  0.0000
condition1:I(minutes_cent^2)               0.02808  0.003337 546   8.41318  0.0000
condition2:I(minutes_cent^2)               0.00215  0.003337 546   0.64309  0.5204
condition3:I(minutes_cent^2)               0.00807  0.003337 546   2.41667  0.0160
condition4:I(minutes_cent^2)              -0.01466  0.003337 546  -4.39234  0.0000
condition5:I(minutes_cent^2)              -0.01876  0.003337 546  -5.62041  0.0000
degrees1:I(minutes_cent^2)                 0.03224  0.001492 546  21.60258  0.0000
condition1:degrees1:minutes_cent          -2.28568  0.306451 546  -7.45856  0.0000
condition2:degrees1:minutes_cent          -0.21024  0.306451 546  -0.68606  0.4930
condition3:degrees1:minutes_cent          -0.78343  0.306451 546  -2.55646  0.0108
condition4:degrees1:minutes_cent           1.28855  0.306451 546   4.20475  0.0000
condition5:degrees1:minutes_cent           1.63388  0.306451 546   5.33161  0.0000
condition1:degrees1:I(minutes_cent^2)     -0.02783  0.003337 546  -8.33876  0.0000
condition2:degrees1:I(minutes_cent^2)     -0.00212  0.003337 546  -0.63384  0.5265
condition3:degrees1:I(minutes_cent^2)     -0.00791  0.003337 546  -2.37140  0.0181
condition4:degrees1:I(minutes_cent^2)      0.01421  0.003337 546   4.25662  0.0000
condition5:degrees1:I(minutes_cent^2)      0.01850  0.003337 546   5.54443  0.0000
 Correlation: 
                                      (Intr) cndtn1 cndtn2 cndtn3 cndtn4 cndtn5 degrs1 mnts_c I(_^2) cnd1:1 cnd2:1 cnd3:1 cnd4:1 cnd5:1 cnd1:_ cnd2:_
condition1                             0.000                                                                                                        
condition2                             0.000 -0.200                                                                                                 
condition3                             0.000 -0.200 -0.200                                                                                          
condition4                             0.000 -0.200 -0.200 -0.200                                                                                   
condition5                             0.000 -0.200 -0.200 -0.200 -0.200                                                                            
degrees1                              -0.670  0.000  0.000  0.000  0.000  0.000                                                                     
minutes_cent                           0.666  0.000  0.000  0.000  0.000  0.000 -0.977                                                              
I(minutes_cent^2)                      0.662  0.000  0.000  0.000  0.000  0.000 -0.959  0.983                                                       
condition1:degrees1                    0.000 -0.968  0.194  0.194  0.194  0.194  0.000  0.000  0.000                                                
condition2:degrees1                    0.000  0.194 -0.968  0.194  0.194  0.194  0.000  0.000  0.000 -0.200                                         
condition3:degrees1                    0.000  0.194  0.194 -0.968  0.194  0.194  0.000  0.000  0.000 -0.200 -0.200                                  
condition4:degrees1                    0.000  0.194  0.194  0.194 -0.968  0.194  0.000  0.000  0.000 -0.200 -0.200 -0.200                           
condition5:degrees1                    0.000  0.194  0.194  0.194  0.194 -0.968  0.000  0.000  0.000 -0.200 -0.200 -0.200 -0.200                    
condition1:minutes_cent                0.000  0.963 -0.193 -0.193 -0.193 -0.193  0.000  0.000  0.000 -0.977  0.195  0.195  0.195  0.195             
condition2:minutes_cent                0.000 -0.193  0.963 -0.193 -0.193 -0.193  0.000  0.000  0.000  0.195 -0.977  0.195  0.195  0.195 -0.200      
condition3:minutes_cent                0.000 -0.193 -0.193  0.963 -0.193 -0.193  0.000  0.000  0.000  0.195  0.195 -0.977  0.195  0.195 -0.200 -0.200
```

```
condition4:minutes_cent              0.000 -0.193 -0.193 -0.193  0.963 -0.193  0.000  0.000  0.000  0.195  0.195  0.195 -0.977  0.195 -0.200 -0.200
condition5:minutes_cent              0.000 -0.193 -0.193 -0.193 -0.193  0.963  0.000  0.000  0.000  0.195  0.195  0.195  0.195 -0.977 -0.200 -0.200
degrees1:minutes_cent               -0.672  0.000  0.000  0.000  0.000  0.000  0.969 -0.996 -0.985  0.000  0.000  0.000  0.000  0.000  0.000  0.000
condition1:I(minutes_cent^2)         0.000  0.957 -0.191 -0.191 -0.191 -0.191  0.000  0.000  0.000 -0.959  0.192  0.192  0.192  0.192  0.983 -0.197
condition2:I(minutes_cent^2)         0.000 -0.191  0.957 -0.191 -0.191 -0.191  0.000  0.000  0.000  0.192 -0.959  0.192  0.192  0.192 -0.197  0.983
condition3:I(minutes_cent^2)         0.000 -0.191 -0.191  0.957 -0.191 -0.191  0.000  0.000  0.000  0.192  0.192 -0.959  0.192  0.192 -0.197 -0.197
condition4:I(minutes_cent^2)         0.000 -0.191 -0.191 -0.191  0.957 -0.191  0.000  0.000  0.000  0.192  0.192  0.192 -0.959  0.192 -0.197 -0.197
condition5:I(minutes_cent^2)         0.000 -0.191 -0.191 -0.191 -0.191  0.957  0.000  0.000  0.000  0.192  0.192  0.192  0.192 -0.959 -0.197 -0.197
degrees1:I(minutes_cent^2)          -0.659  0.000  0.000  0.000  0.000  0.000  0.962 -0.985 -0.999  0.000  0.000  0.000  0.000  0.000  0.000  0.000
condition1:degrees1:minutes_cent     0.000 -0.972  0.194  0.194  0.194  0.194  0.000  0.000  0.000  0.969 -0.194 -0.194 -0.194 -0.194 -0.996  0.199
condition2:degrees1:minutes_cent     0.000  0.194 -0.972  0.194  0.194  0.194  0.000  0.000  0.000 -0.194  0.969 -0.194 -0.194 -0.194  0.199 -0.996
condition3:degrees1:minutes_cent     0.000  0.194  0.194 -0.972  0.194  0.194  0.000  0.000  0.000 -0.194 -0.194  0.969 -0.194 -0.194  0.199  0.199
condition4:degrees1:minutes_cent     0.000  0.194  0.194  0.194 -0.972  0.194  0.000  0.000  0.000 -0.194 -0.194 -0.194  0.969 -0.194  0.199  0.199
condition5:degrees1:minutes_cent     0.000  0.194  0.194  0.194  0.194 -0.972  0.000  0.000  0.000 -0.194 -0.194 -0.194 -0.194  0.969  0.199  0.199
condition1:degrees1:I(minutes_cent^2)  0.000 -0.953  0.191  0.191  0.191  0.191  0.000  0.000  0.000  0.962 -0.192 -0.192 -0.192 -0.192 -0.985  0.197
condition2:degrees1:I(minutes_cent^2)  0.000  0.191 -0.953  0.191  0.191  0.191  0.000  0.000  0.000 -0.192  0.962 -0.192 -0.192 -0.192  0.197 -0.985
condition3:degrees1:I(minutes_cent^2)  0.000  0.191  0.191 -0.953  0.191  0.191  0.000  0.000  0.000 -0.192 -0.192  0.962 -0.192 -0.192  0.197  0.197
condition4:degrees1:I(minutes_cent^2)  0.000  0.191  0.191  0.191 -0.953  0.191  0.000  0.000  0.000 -0.192 -0.192 -0.192  0.962 -0.192  0.197  0.197
condition5:degrees1:I(minutes_cent^2)  0.000  0.191  0.191  0.191  0.191 -0.953  0.000  0.000  0.000 -0.192 -0.192 -0.192 -0.192  0.962  0.197  0.197
                                    cnd3:_ cnd4:_ cnd5:_ dgr1:_ c1:I(_ c2:I(_ c3:I(_ c4:I(_ c5:I(_ d1:I(_ c1:1:_ c2:1:_ c3:1:_ c4:1:_ c5:1:_ c1:1:I
condition1
condition2
condition3
condition4
condition5
degrees1
minutes_cent
I(minutes_cent^2)
condition1:degrees1
condition2:degrees1
condition3:degrees1
condition4:degrees1
condition5:degrees1
condition1:minutes_cent
condition2:minutes_cent
condition3:minutes_cent
condition4:minutes_cent             -0.200
condition5:minutes_cent             -0.200 -0.200
degrees1:minutes_cent                0.000  0.000  0.000
condition1:I(minutes_cent^2)        -0.197 -0.197 -0.197  0.000
condition2:I(minutes_cent^2)        -0.197 -0.197 -0.197  0.000 -0.200
condition3:I(minutes_cent^2)         0.983 -0.197 -0.197  0.000 -0.200 -0.200
condition4:I(minutes_cent^2)        -0.197  0.983 -0.197  0.000 -0.200 -0.200 -0.200
condition5:I(minutes_cent^2)        -0.197 -0.197  0.983  0.000 -0.200 -0.200 -0.200 -0.200
degrees1:I(minutes_cent^2)           0.000  0.000  0.000  0.983  0.000  0.000  0.000  0.000  0.000
condition1:degrees1:minutes_cent     0.199  0.199  0.199  0.000 -0.985  0.197  0.197  0.197  0.197  0.000
condition2:degrees1:minutes_cent     0.199  0.199  0.199  0.000  0.197 -0.985  0.197  0.197  0.197  0.000 -0.200
condition3:degrees1:minutes_cent    -0.996  0.199  0.199  0.000  0.197  0.197 -0.985  0.197  0.197  0.000 -0.200 -0.200
```

```
condition4:degrees1:minutes_cent       0.199 -0.996  0.199  0.000  0.197  0.197  0.197 -0.985  0.197  0.000 -0.200 -0.200 -0.200
condition5:degrees1:minutes_cent       0.199  0.199 -0.996  0.000  0.197  0.197  0.197  0.197 -0.985  0.000 -0.200 -0.200 -0.200 -0.200
condition1:degrees1:I(minutes_cent^2)  0.197  0.197  0.197  0.000 -0.999  0.200  0.200  0.200  0.200  0.000  0.983 -0.197 -0.197 -0.197 -0.197
condition2:degrees1:I(minutes_cent^2)  0.197  0.197  0.197  0.000  0.200 -0.999  0.200  0.200  0.200  0.000 -0.197  0.983 -0.197 -0.197 -0.197 -0.200
condition3:degrees1:I(minutes_cent^2) -0.985  0.197  0.197  0.000  0.200  0.200 -0.999  0.200  0.200  0.000 -0.197 -0.197  0.983 -0.197 -0.197 -0.200
condition4:degrees1:I(minutes_cent^2)  0.197 -0.985  0.197  0.000  0.200  0.200  0.200 -0.999  0.200  0.000 -0.197 -0.197 -0.197  0.983 -0.197 -0.200
condition5:degrees1:I(minutes_cent^2)  0.197  0.197 -0.985  0.000  0.200  0.200  0.200  0.200 -0.999  0.000 -0.197 -0.197 -0.197 -0.197  0.983 -0.200
                                      c2:1:I c3:1:I c4:1:I
condition1
condition2
condition3
condition4
condition5
degrees1
minutes_cent
I(minutes_cent^2)
condition1:degrees1
condition2:degrees1
condition3:degrees1
condition4:degrees1
condition5:degrees1
condition1:minutes_cent
condition2:minutes_cent
condition3:minutes_cent
condition4:minutes_cent
condition5:minutes_cent
degrees1:minutes_cent
condition1:I(minutes_cent^2)
condition2:I(minutes_cent^2)
condition3:I(minutes_cent^2)
condition4:I(minutes_cent^2)
condition5:I(minutes_cent^2)
degrees1:I(minutes_cent^2)
condition1:degrees1:minutes_cent
condition2:degrees1:minutes_cent
condition3:degrees1:minutes_cent
condition4:degrees1:minutes_cent
condition5:degrees1:minutes_cent
condition1:degrees1:I(minutes_cent^2)
condition2:degrees1:I(minutes_cent^2)
condition3:degrees1:I(minutes_cent^2) -0.200
condition4:degrees1:I(minutes_cent^2) -0.200 -0.200
condition5:degrees1:I(minutes_cent^2) -0.200 -0.200 -0.200

Standardized Within-Group Residuals:
        Min          Q1         Med          Q3         Max
-2.66052130 -0.76335837 -0.07624293  0.53944836  2.16614447
```

```
Number of Observations: 630
Number of Groups:
                              virus_batch                          date %in% virus_batch          test_tube %in% date %in% virus_batch
                                        2                                              5                                            30
unit %in% test_tube %in% date %in% virus_batch
                                       60
```

4.4.2 Model with Two Random Effects (no Variance or Correlation Structures)

```
Linear mixed-effects model fit by REML
 Data: dta
       AIC      BIC    logLik
  3780.107 3951.195 -1851.053

Random effects:
 Formula: ~1 | date
        (Intercept)
StdDev:    1.892305

 Formula: ~1 | unit %in% date
        (Intercept) Residual
StdDev:    4.272279   3.5023

Fixed effects: mV ~ condition * degrees * minutes_cent + condition * degrees *      I(minutes_cent^2)
                                           Value Std.Error  DF   t-value p-value
(Intercept)                           -228.66195  5.298086 546 -43.15935  0.0000
condition1                              33.62417 11.694776  44   2.87514  0.0062
condition2                               7.45997 11.694776  44   0.63789  0.5269
condition3                              20.76469 11.694776  44   1.77555  0.0827
condition4                             -26.77816 11.694776  44  -2.28975  0.0269
condition5                             -34.74679 11.694776  44  -2.97114  0.0048
degrees1                                43.87739  5.230063  44   8.38946  0.0000
minutes_cent                            -2.49261  0.225111 546 -11.07280  0.0000
I(minutes_cent^2)                       -0.03447  0.002374 546 -14.52101  0.0000
condition1:degrees1                    -39.17269 11.694776  44  -3.34959  0.0017
condition2:degrees1                    -10.01567 11.694776  44  -0.85642  0.3964
condition3:degrees1                    -23.11349 11.694776  44  -1.97639  0.0544
condition4:degrees1                     30.94591 11.694776  44   2.64613  0.0113
condition5:degrees1                     38.85165 11.694776  44   3.32214  0.0018
condition1:minutes_cent                  2.38959  0.503363 546   4.74724  0.0000
condition2:minutes_cent                  0.27988  0.503363 546   0.55602  0.5784
condition3:minutes_cent                  0.94091  0.503363 546   1.86924  0.0621
condition4:minutes_cent                 -1.42836  0.503363 546  -2.83763  0.0047
condition5:minutes_cent                 -1.84102  0.503363 546  -3.65744  0.0003
degrees1:minutes_cent                    2.69016  0.225111 546  11.95040  0.0000
condition1:I(minutes_cent^2)             0.02997  0.005308 546   5.64647  0.0000
condition2:I(minutes_cent^2)             0.00288  0.005308 546   0.54332  0.5871
```

```
condition3:I(minutes_cent^2)            0.00964  0.005308 546   1.81671  0.0698
condition4:I(minutes_cent^2)           -0.01672  0.005308 546  -3.14988  0.0017
condition5:I(minutes_cent^2)           -0.02139  0.005308 546  -4.03040  0.0001
degrees1:I(minutes_cent^2)              0.03368  0.002374 546  14.18876  0.0000
condition1:degrees1:minutes_cent       -2.47550  0.503363 546  -4.91792  0.0000
condition2:degrees1:minutes_cent       -0.28483  0.503363 546  -0.56586  0.5717
condition3:degrees1:minutes_cent       -0.94487  0.503363 546  -1.87711  0.0610
condition4:degrees1:minutes_cent        1.49984  0.503363 546   2.97965  0.0030
condition5:degrees1:minutes_cent        1.90187  0.503363 546   3.77832  0.0002
condition1:degrees1:I(minutes_cent^2)  -0.02973  0.005308 546  -5.60062  0.0000
condition2:degrees1:I(minutes_cent^2)  -0.00261  0.005308 546  -0.49205  0.6229
condition3:degrees1:I(minutes_cent^2)  -0.00954  0.005308 546  -1.79776  0.0728
condition4:degrees1:I(minutes_cent^2)   0.01628  0.005308 546   3.06611  0.0023
condition5:degrees1:I(minutes_cent^2)   0.02113  0.005308 546   3.97974  0.0001
 Correlation:
                                      (Intr) cndtn1 cndtn2 cndtn3 cndtn4 cndtn5 degrs1 mnts_c I(_^2) cnd1:1 cnd2:1 cnd3:1 cnd4:1 cnd5:1 cnd1:_ cnd2:_
condition1                             0.000
condition2                             0.000 -0.200
condition3                             0.000 -0.200 -0.200
condition4                             0.000 -0.200 -0.200 -0.200
condition5                             0.000 -0.200 -0.200 -0.200 -0.200
degrees1                              -0.975  0.000  0.000  0.000  0.000  0.000
minutes_cent                           0.978  0.000  0.000  0.000  0.000  0.000 -0.991
I(minutes_cent^2)                      0.969  0.000  0.000  0.000  0.000  0.000 -0.982  0.997
condition1:degrees1                    0.000 -0.988  0.198  0.198  0.198  0.198  0.000  0.000  0.000
condition2:degrees1                    0.000  0.198 -0.988  0.198  0.198  0.198  0.000  0.000  0.000 -0.200
condition3:degrees1                    0.000  0.198  0.198 -0.988  0.198  0.198  0.000  0.000  0.000 -0.200 -0.200
condition4:degrees1                    0.000  0.198  0.198  0.198 -0.988  0.198  0.000  0.000  0.000 -0.200 -0.200 -0.200
condition5:degrees1                    0.000  0.198  0.198  0.198  0.198 -0.988  0.000  0.000  0.000 -0.200 -0.200 -0.200 -0.200
condition1:minutes_cent                0.000  0.991 -0.198 -0.198 -0.198 -0.198  0.000  0.000  0.000 -0.991  0.198  0.198  0.198  0.198
condition2:minutes_cent                0.000 -0.198  0.991 -0.198 -0.198 -0.198  0.000  0.000  0.000  0.198 -0.991  0.198  0.198  0.198 -0.200
condition3:minutes_cent                0.000 -0.198 -0.198  0.991 -0.198 -0.198  0.000  0.000  0.000  0.198  0.198 -0.991  0.198  0.198 -0.200 -0.200
condition4:minutes_cent                0.000 -0.198 -0.198 -0.198  0.991 -0.198  0.000  0.000  0.000  0.198  0.198  0.198 -0.991  0.198 -0.200 -0.200
condition5:minutes_cent                0.000 -0.198 -0.198 -0.198 -0.198  0.991  0.000  0.000  0.000  0.198  0.198  0.198  0.198 -0.991 -0.200 -0.200
degrees1:minutes_cent                 -0.978  0.000  0.000  0.000  0.000  0.000  0.991 -1.000 -0.997  0.000  0.000  0.000  0.000  0.000  0.000  0.000
condition1:I(minutes_cent^2)           0.000  0.982 -0.196 -0.196 -0.196 -0.196  0.000  0.000  0.000 -0.982  0.196  0.196  0.196  0.196  0.997 -0.199
condition2:I(minutes_cent^2)           0.000 -0.196  0.982 -0.196 -0.196 -0.196  0.000  0.000  0.000  0.196 -0.982  0.196  0.196  0.196 -0.199  0.997
condition3:I(minutes_cent^2)           0.000 -0.196 -0.196  0.982 -0.196 -0.196  0.000  0.000  0.000  0.196  0.196 -0.982  0.196  0.196 -0.199 -0.199
condition4:I(minutes_cent^2)           0.000 -0.196 -0.196 -0.196  0.982 -0.196  0.000  0.000  0.000  0.196  0.196  0.196 -0.982  0.196 -0.199 -0.199
condition5:I(minutes_cent^2)           0.000 -0.196 -0.196 -0.196 -0.196  0.982  0.000  0.000  0.000  0.196  0.196  0.196  0.196 -0.982 -0.199 -0.199
degrees1:I(minutes_cent^2)            -0.970  0.000  0.000  0.000  0.000  0.000  0.982 -0.997 -0.999  0.000  0.000  0.000  0.000  0.000  0.000  0.000
condition1:degrees1:minutes_cent       0.000 -0.991  0.198  0.198  0.198  0.198  0.000  0.000  0.000  0.991 -0.198 -0.198 -0.198 -0.198 -1.000  0.200
condition2:degrees1:minutes_cent       0.000  0.198 -0.991  0.198  0.198  0.198  0.000  0.000  0.000 -0.198  0.991 -0.198 -0.198 -0.198  0.200 -1.000
condition3:degrees1:minutes_cent       0.000  0.198  0.198 -0.991  0.198  0.198  0.000  0.000  0.000 -0.198 -0.198  0.991 -0.198 -0.198  0.200  0.200
condition4:degrees1:minutes_cent       0.000  0.198  0.198  0.198 -0.991  0.198  0.000  0.000  0.000 -0.198 -0.198 -0.198  0.991 -0.198  0.200  0.200
condition5:degrees1:minutes_cent       0.000  0.198  0.198  0.198  0.198 -0.991  0.000  0.000  0.000 -0.198 -0.198 -0.198 -0.198  0.991  0.200  0.200
condition1:degrees1:I(minutes_cent^2)  0.000 -0.982  0.196  0.196  0.196  0.196  0.000  0.000  0.000  0.982 -0.196 -0.196 -0.196 -0.196 -0.997  0.199
condition2:degrees1:I(minutes_cent^2)  0.000  0.196 -0.982  0.196  0.196  0.196  0.000  0.000  0.000 -0.196  0.982 -0.196 -0.196 -0.196  0.199 -0.997
```

```
condition3:degrees1:I(minutes_cent^2)  0.000  0.196  0.196 -0.982  0.196  0.196  0.000  0.000  0.000 -0.196 -0.196  0.982 -0.196 -0.196  0.199  0.199
condition4:degrees1:I(minutes_cent^2)  0.000  0.196  0.196  0.196 -0.982  0.196  0.000  0.000  0.000 -0.196 -0.196 -0.196  0.982 -0.196  0.199  0.199
condition5:degrees1:I(minutes_cent^2)  0.000  0.196  0.196  0.196  0.196 -0.982  0.000  0.000  0.000 -0.196 -0.196 -0.196 -0.196  0.982  0.199  0.199
                                      cnd3:_ cnd4:_ cnd5:_ dgr1:_ c1:I(_ c2:I(_ c3:I(_ c4:I(_ c5:I(_ d1:I(_ c1:1:_ c2:1:_ c3:1:_ c4:1:_ c5:1:_ c1:1:I
condition1
condition2
condition3
condition4
condition5
degrees1
minutes_cent
I(minutes_cent^2)
condition1:degrees1
condition2:degrees1
condition3:degrees1
condition4:degrees1
condition5:degrees1
condition1:minutes_cent
condition2:minutes_cent
condition3:minutes_cent
condition4:minutes_cent               -0.200
condition5:minutes_cent               -0.200 -0.200
degrees1:minutes_cent                  0.000  0.000  0.000
condition1:I(minutes_cent^2)          -0.199 -0.199 -0.199  0.000
condition2:I(minutes_cent^2)          -0.199 -0.199 -0.199  0.000 -0.200
condition3:I(minutes_cent^2)           0.997 -0.199 -0.199  0.000 -0.200 -0.200
condition4:I(minutes_cent^2)          -0.199  0.997 -0.199  0.000 -0.200 -0.200 -0.200
condition5:I(minutes_cent^2)          -0.199 -0.199  0.997  0.000 -0.200 -0.200 -0.200 -0.200
degrees1:I(minutes_cent^2)             0.000  0.000  0.000  0.997  0.000  0.000  0.000  0.000  0.000
condition1:degrees1:minutes_cent       0.200  0.200  0.200  0.000 -0.997  0.199  0.199  0.199  0.199  0.000
condition2:degrees1:minutes_cent       0.200  0.200  0.200  0.000  0.199 -0.997  0.199  0.199  0.199  0.000 -0.200
condition3:degrees1:minutes_cent      -1.000  0.200  0.200  0.000  0.199  0.199 -0.997  0.199  0.199  0.000 -0.200 -0.200
condition4:degrees1:minutes_cent       0.200 -1.000  0.200  0.000  0.199  0.199  0.199 -0.997  0.199  0.000 -0.200 -0.200 -0.200
condition5:degrees1:minutes_cent       0.200  0.200 -1.000  0.000  0.199  0.199  0.199  0.199 -0.997  0.000 -0.200 -0.200 -0.200 -0.200
condition1:degrees1:I(minutes_cent^2)  0.199  0.199  0.199  0.000 -0.999  0.200  0.200  0.200  0.200  0.000  0.997 -0.199 -0.199 -0.199 -0.199
condition2:degrees1:I(minutes_cent^2)  0.199  0.199  0.199  0.000  0.200 -0.999  0.200  0.200  0.200  0.000 -0.199  0.997 -0.199 -0.199 -0.199 -0.200
condition3:degrees1:I(minutes_cent^2) -0.997  0.199  0.199  0.000  0.200  0.200 -0.999  0.200  0.200  0.000 -0.199 -0.199  0.997 -0.199 -0.199 -0.200
condition4:degrees1:I(minutes_cent^2)  0.199 -0.997  0.199  0.000  0.200  0.200  0.200 -0.999  0.200  0.000 -0.199 -0.199 -0.199  0.997 -0.199 -0.200
condition5:degrees1:I(minutes_cent^2)  0.199  0.199 -0.997  0.000  0.200  0.200  0.200  0.200 -0.999  0.000 -0.199 -0.199 -0.199 -0.199  0.997 -0.200
                                      c2:1:I c3:1:I c4:1:I
condition1
condition2
condition3
condition4
condition5
degrees1
minutes_cent
I(minutes_cent^2)
```

```
condition1:degrees1
condition2:degrees1
condition3:degrees1
condition4:degrees1
condition5:degrees1
condition1:minutes_cent
condition2:minutes_cent
condition3:minutes_cent
condition4:minutes_cent
condition5:minutes_cent
degrees1:minutes_cent
condition1:I(minutes_cent^2)
condition2:I(minutes_cent^2)
condition3:I(minutes_cent^2)
condition4:I(minutes_cent^2)
condition5:I(minutes_cent^2)
degrees1:I(minutes_cent^2)
condition1:degrees1:minutes_cent
condition2:degrees1:minutes_cent
condition3:degrees1:minutes_cent
condition4:degrees1:minutes_cent
condition5:degrees1:minutes_cent
condition1:degrees1:I(minutes_cent^2)
condition2:degrees1:I(minutes_cent^2)
condition3:degrees1:I(minutes_cent^2) -0.200
condition4:degrees1:I(minutes_cent^2) -0.200 -0.200
condition5:degrees1:I(minutes_cent^2) -0.200 -0.200 -0.200

Standardized Within-Group Residuals:
         Min           Q1          Med           Q3          Max
-4.535512492 -0.441673220 -0.008280167  0.502118159  3.359374056

Number of Observations: 630
Number of Groups:
          date unit %in% date
             5            60
```

4.4.3 Model with Four Random Effects (no Variance or Correlation Structures)

```
Linear mixed-effects model fit by REML
 Data: dta
       AIC      BIC    logLik
  3780.467 3960.329 -1849.233

Random effects:
 Formula: ~1 | virus_batch
        (Intercept)
```

```
StdDev: 0.001382475

 Formula: ~1 | date %in% virus_batch
        (Intercept)
StdDev:    1.687449

 Formula: ~1 | test_tube %in% date %in% virus_batch
        (Intercept)
StdDev:    2.789745

 Formula: ~1 | unit %in% test_tube %in% date %in% virus_batch
        (Intercept) Residual
StdDev:     3.32719 3.502683

Fixed effects: mV ~ condition * degrees * minutes_cent + condition * degrees *      I(minutes_cent^2)
                                          Value Std.Error  DF   t-value p-value
(Intercept)                           -228.66195  5.297988 546 -43.16015  0.0000
condition1                              33.62417 11.725865  20   2.86752  0.0095
condition2                               7.45997 11.725865  20   0.63620  0.5319
condition3                              20.76469 11.725865  20   1.77084  0.0918
condition4                             -26.77816 11.725865  20  -2.28368  0.0335
condition5                             -34.74679 11.725865  20  -2.96326  0.0077
degrees1                                43.87739  5.219172  24   8.40696  0.0000
minutes_cent                            -2.49261  0.225135 546 -11.07160  0.0000
I(minutes_cent^2)                       -0.03447  0.002374 546 -14.51942  0.0000
condition1:degrees1                    -39.17269 11.670424  24  -3.35658  0.0026
condition2:degrees1                    -10.01567 11.670424  24  -0.85821  0.3993
condition3:degrees1                    -23.11349 11.670424  24  -1.98052  0.0592
condition4:degrees1                     30.94591 11.670424  24   2.65165  0.0140
condition5:degrees1                     38.85165 11.670424  24   3.32907  0.0028
condition1:minutes_cent                  2.38959  0.503418 546   4.74672  0.0000
condition2:minutes_cent                  0.27988  0.503418 546   0.55595  0.5785
condition3:minutes_cent                  0.94091  0.503418 546   1.86903  0.0622
condition4:minutes_cent                 -1.42836  0.503418 546  -2.83732  0.0047
condition5:minutes_cent                 -1.84102  0.503418 546  -3.65704  0.0003
degrees1:minutes_cent                    2.69016  0.225135 546  11.94909  0.0000
condition1:I(minutes_cent^2)             0.02997  0.005309 546   5.64585  0.0000
condition2:I(minutes_cent^2)             0.00288  0.005309 546   0.54326  0.5872
condition3:I(minutes_cent^2)             0.00964  0.005309 546   1.81651  0.0698
condition4:I(minutes_cent^2)            -0.01672  0.005309 546  -3.14953  0.0017
condition5:I(minutes_cent^2)            -0.02139  0.005309 546  -4.02996  0.0001
degrees1:I(minutes_cent^2)               0.03368  0.002374 546  14.18721  0.0000
condition1:degrees1:minutes_cent        -2.47550  0.503418 546  -4.91738  0.0000
condition2:degrees1:minutes_cent        -0.28483  0.503418 546  -0.56580  0.5718
condition3:degrees1:minutes_cent        -0.94487  0.503418 546  -1.87691  0.0611
condition4:degrees1:minutes_cent         1.49984  0.503418 546   2.97932  0.0030
condition5:degrees1:minutes_cent         1.90187  0.503418 546   3.77790  0.0002
condition1:degrees1:I(minutes_cent^2)   -0.02973  0.005309 546  -5.60001  0.0000
```

```
condition2:degrees1:I(minutes_cent^2)   -0.00261  0.005309 546  -0.49200  0.6229
condition3:degrees1:I(minutes_cent^2)   -0.00954  0.005309 546  -1.79756  0.0728
condition4:degrees1:I(minutes_cent^2)    0.01628  0.005309 546   3.06578  0.0023
condition5:degrees1:I(minutes_cent^2)    0.02113  0.005309 546   3.97930  0.0001
 Correlation: 
                                      (Intr) cndtn1 cndtn2 cndtn3 cndtn4 cndtn5 degrs1 mnts_c I(_^2) cnd1:1 cnd2:1 cnd3:1 cnd4:1 cnd5:1 cnd1:_ cnd2:_
condition1                             0.000                                                                                                          
condition2                             0.000 -0.200                                                                                                   
condition3                             0.000 -0.200 -0.200                                                                                            
condition4                             0.000 -0.200 -0.200 -0.200                                                                                     
condition5                             0.000 -0.200 -0.200 -0.200 -0.200                                                                              
degrees1                              -0.977  0.000  0.000  0.000  0.000  0.000                                                                       
minutes_cent                           0.978  0.000  0.000  0.000  0.000  0.000 -0.993                                                                
I(minutes_cent^2)                      0.969  0.000  0.000  0.000  0.000  0.000 -0.984  0.997                                                         
condition1:degrees1                    0.000 -0.988  0.198  0.198  0.198  0.198  0.000  0.000  0.000                                                  
condition2:degrees1                    0.000  0.198 -0.988  0.198  0.198  0.198  0.000  0.000  0.000 -0.200                                           
condition3:degrees1                    0.000  0.198  0.198 -0.988  0.198  0.198  0.000  0.000  0.000 -0.200 -0.200                                    
condition4:degrees1                    0.000  0.198  0.198  0.198 -0.988  0.198  0.000  0.000  0.000 -0.200 -0.200 -0.200                             
condition5:degrees1                    0.000  0.198  0.198  0.198  0.198 -0.988  0.000  0.000  0.000 -0.200 -0.200 -0.200 -0.200                      
condition1:minutes_cent                0.000  0.988 -0.198 -0.198 -0.198 -0.198  0.000  0.000  0.000 -0.993  0.199  0.199  0.199  0.199               
condition2:minutes_cent                0.000 -0.198  0.988 -0.198 -0.198 -0.198  0.000  0.000  0.000  0.199 -0.993  0.199  0.199  0.199 -0.200        
condition3:minutes_cent                0.000 -0.198 -0.198  0.988 -0.198 -0.198  0.000  0.000  0.000  0.199  0.199 -0.993  0.199  0.199 -0.200 -0.200
condition4:minutes_cent                0.000 -0.198 -0.198 -0.198  0.988 -0.198  0.000  0.000  0.000  0.199  0.199  0.199 -0.993  0.199 -0.200 -0.200
condition5:minutes_cent                0.000 -0.198 -0.198 -0.198 -0.198  0.988  0.000  0.000  0.000  0.199  0.199  0.199  0.199 -0.993 -0.200 -0.200
degrees1:minutes_cent                 -0.978  0.000  0.000  0.000  0.000  0.000  0.993 -1.000 -0.997  0.000  0.000  0.000  0.000  0.000  0.000  0.000
condition1:I(minutes_cent^2)           0.000  0.979 -0.196 -0.196 -0.196 -0.196  0.000  0.000  0.000 -0.984  0.197  0.197  0.197  0.197  0.997 -0.199
condition2:I(minutes_cent^2)           0.000 -0.196  0.979 -0.196 -0.196 -0.196  0.000  0.000  0.000  0.197 -0.984  0.197  0.197  0.197 -0.199  0.997
condition3:I(minutes_cent^2)           0.000 -0.196 -0.196  0.979 -0.196 -0.196  0.000  0.000  0.000  0.197  0.197 -0.984  0.197  0.197 -0.199 -0.199
condition4:I(minutes_cent^2)           0.000 -0.196 -0.196 -0.196  0.979 -0.196  0.000  0.000  0.000  0.197  0.197  0.197 -0.984  0.197 -0.199 -0.199
condition5:I(minutes_cent^2)           0.000 -0.196 -0.196 -0.196 -0.196  0.979  0.000  0.000  0.000  0.197  0.197  0.197  0.197 -0.984 -0.199 -0.199
degrees1:I(minutes_cent^2)            -0.970  0.000  0.000  0.000  0.000  0.000  0.984 -0.997 -0.999  0.000  0.000  0.000  0.000  0.000  0.000  0.000
condition1:degrees1:minutes_cent       0.000 -0.988  0.198  0.198  0.198  0.198  0.000  0.000  0.000  0.993 -0.199 -0.199 -0.199 -0.199 -1.000  0.200
condition2:degrees1:minutes_cent       0.000  0.198 -0.988  0.198  0.198  0.198  0.000  0.000  0.000 -0.199  0.993 -0.199 -0.199 -0.199  0.200 -1.000
condition3:degrees1:minutes_cent       0.000  0.198  0.198 -0.988  0.198  0.198  0.000  0.000  0.000 -0.199 -0.199  0.993 -0.199 -0.199  0.200  0.200
condition4:degrees1:minutes_cent       0.000  0.198  0.198  0.198 -0.988  0.198  0.000  0.000  0.000 -0.199 -0.199 -0.199  0.993 -0.199  0.200  0.200
condition5:degrees1:minutes_cent       0.000  0.198  0.198  0.198  0.198 -0.988  0.000  0.000  0.000 -0.199 -0.199 -0.199 -0.199  0.993  0.200  0.200
condition1:degrees1:I(minutes_cent^2)  0.000 -0.980  0.196  0.196  0.196  0.196  0.000  0.000  0.000  0.984 -0.197 -0.197 -0.197 -0.197 -0.997  0.199
condition2:degrees1:I(minutes_cent^2)  0.000  0.196 -0.980  0.196  0.196  0.196  0.000  0.000  0.000 -0.197  0.984 -0.197 -0.197 -0.197  0.199 -0.997
condition3:degrees1:I(minutes_cent^2)  0.000  0.196  0.196 -0.980  0.196  0.196  0.000  0.000  0.000 -0.197 -0.197  0.984 -0.197 -0.197  0.199  0.199
condition4:degrees1:I(minutes_cent^2)  0.000  0.196  0.196  0.196 -0.980  0.196  0.000  0.000  0.000 -0.197 -0.197 -0.197  0.984 -0.197  0.199  0.199
condition5:degrees1:I(minutes_cent^2)  0.000  0.196  0.196  0.196  0.196 -0.980  0.000  0.000  0.000 -0.197 -0.197 -0.197 -0.197  0.984  0.199  0.199
                                      cnd3:_ cnd4:_ cnd5:_ dgr1:_ c1:I(_ c2:I(_ c3:I(_ c4:I(_ c5:I(_ d1:I(_ c1:1:_ c2:1:_ c3:1:_ c4:1:_ c5:1:_ c1:1:I
condition1                                                                                                                                            
condition2                                                                                                                                            
condition3                                                                                                                                            
condition4                                                                                                                                            
condition5                                                                                                                                            
degrees1                                                                                                                                              
```

```
minutes_cent
I(minutes_cent^2)
condition1:degrees1
condition2:degrees1
condition3:degrees1
condition4:degrees1
condition5:degrees1
condition1:minutes_cent
condition2:minutes_cent
condition3:minutes_cent
condition4:minutes_cent               -0.200
condition5:minutes_cent               -0.200 -0.200
degrees1:minutes_cent                  0.000  0.000  0.000
condition1:I(minutes_cent^2)          -0.199 -0.199 -0.199  0.000
condition2:I(minutes_cent^2)          -0.199 -0.199 -0.199  0.000 -0.200
condition3:I(minutes_cent^2)           0.997 -0.199 -0.199  0.000 -0.200 -0.200
condition4:I(minutes_cent^2)          -0.199  0.997 -0.199  0.000 -0.200 -0.200 -0.200
condition5:I(minutes_cent^2)          -0.199 -0.199  0.997  0.000 -0.200 -0.200 -0.200 -0.200
degrees1:I(minutes_cent^2)             0.000  0.000  0.000  0.997  0.000  0.000  0.000  0.000  0.000
condition1:degrees1:minutes_cent       0.200  0.200  0.200  0.000 -0.997  0.199  0.199  0.199  0.199  0.000
condition2:degrees1:minutes_cent       0.200  0.200  0.200  0.000  0.199 -0.997  0.199  0.199  0.199  0.000 -0.200
condition3:degrees1:minutes_cent      -1.000  0.200  0.200  0.000  0.199  0.199 -0.997  0.199  0.199  0.000 -0.200 -0.200
condition4:degrees1:minutes_cent       0.200 -1.000  0.200  0.000  0.199  0.199  0.199 -0.997  0.199  0.000 -0.200 -0.200 -0.200
condition5:degrees1:minutes_cent       0.200  0.200 -1.000  0.000  0.199  0.199  0.199  0.199 -0.997  0.000 -0.200 -0.200 -0.200 -0.200
condition1:degrees1:I(minutes_cent^2)  0.199  0.199  0.199  0.000 -0.999  0.200  0.200  0.200  0.200  0.000  0.997 -0.199 -0.199 -0.199 -0.199
condition2:degrees1:I(minutes_cent^2)  0.199  0.199  0.199  0.000  0.200 -0.999  0.200  0.200  0.200  0.000 -0.199  0.997 -0.199 -0.199 -0.199 -0.200
condition3:degrees1:I(minutes_cent^2) -0.997  0.199  0.199  0.000  0.200  0.200 -0.999  0.200  0.200  0.000 -0.199 -0.199  0.997 -0.199 -0.199 -0.200
condition4:degrees1:I(minutes_cent^2)  0.199 -0.997  0.199  0.000  0.200  0.200  0.200 -0.999  0.200  0.000 -0.199 -0.199 -0.199  0.997 -0.199 -0.200
condition5:degrees1:I(minutes_cent^2)  0.199  0.199 -0.997  0.000  0.200  0.200  0.200  0.200 -0.999  0.000 -0.199 -0.199 -0.199 -0.199  0.997 -0.200
                                      c2:1:I c3:1:I c4:1:I
condition1
condition2
condition3
condition4
condition5
degrees1
minutes_cent
I(minutes_cent^2)
condition1:degrees1
condition2:degrees1
condition3:degrees1
condition4:degrees1
condition5:degrees1
condition1:minutes_cent
condition2:minutes_cent
condition3:minutes_cent
condition4:minutes_cent
condition5:minutes_cent
```

```
degrees1:minutes_cent
condition1:I(minutes_cent^2)
condition2:I(minutes_cent^2)
condition3:I(minutes_cent^2)
condition4:I(minutes_cent^2)
condition5:I(minutes_cent^2)
degrees1:I(minutes_cent^2)
condition1:degrees1:minutes_cent
condition2:degrees1:minutes_cent
condition3:degrees1:minutes_cent
condition4:degrees1:minutes_cent
condition5:degrees1:minutes_cent
condition1:degrees1:I(minutes_cent^2)
condition2:degrees1:I(minutes_cent^2)
condition3:degrees1:I(minutes_cent^2) -0.200
condition4:degrees1:I(minutes_cent^2) -0.200 -0.200
condition5:degrees1:I(minutes_cent^2) -0.200 -0.200 -0.200

Standardized Within-Group Residuals:
         Min           Q1          Med           Q3          Max
-4.481292451 -0.437991907 -0.007573324  0.510569896  3.375337072

Number of Observations: 630
Number of Groups:
                                   virus_batch                          date %in% virus_batch           test_tube %in% date %in% virus_batch
                                             2                                              5                                             30
unit %in% test_tube %in% date %in% virus_batch
                                            60
```

4.4.4 Model with Four Random Effects and a Correlation Structure (no Variance Structure)

```
Linear mixed-effects model fit by REML
 Data: dta
       AIC      BIC    logLik
  3375.728 3564.364 -1644.864

Random effects:
 Formula: ~1 | virus_batch
         (Intercept)
StdDev: 4.210617e-30

 Formula: ~1 | date %in% virus_batch
        (Intercept)
StdDev:    2.654779

 Formula: ~1 | test_tube %in% date %in% virus_batch
        (Intercept)
```

```
StdDev: 0.001887153

 Formula: ~1 | unit %in% test_tube %in% date %in% virus_batch
         (Intercept) Residual
StdDev: 2.385793e-25 6.040134

Correlation Structure: Exponential spatial correlation
 Formula: ~minutes_cent | virus_batch/date/test_tube/unit
 Parameter estimate(s):
       range       nugget
104.57180936   0.02453973
Fixed effects: mV ~ condition * degrees * minutes_cent + condition * degrees *      I(minutes_cent^2)
                                           Value Std.Error  DF   t-value p-value
(Intercept)                           -227.49198  3.193366 546 -71.23893  0.0000
condition1                              32.80191  6.628732  20   4.94844  0.0001
condition2                               7.62580  6.628732  20   1.15042  0.2635
condition3                              20.46451  6.628732  20   3.08724  0.0058
condition4                             -26.77063  6.628732  20  -4.03858  0.0006
condition5                             -34.19253  6.628732  20  -5.15823  0.0000
degrees1                                42.63618  2.964459  24  14.38245  0.0000
minutes_cent                            -2.45076  0.125421 546 -19.54028  0.0000
I(minutes_cent^2)                       -0.03414  0.001317 546 -25.91968  0.0000
condition1:degrees1                    -38.37766  6.628732  24  -5.78959  0.0000
condition2:degrees1                    -10.12736  6.628732  24  -1.52780  0.1396
condition3:degrees1                    -22.78745  6.628732  24  -3.43768  0.0021
condition4:degrees1                     30.91336  6.628732  24   4.66354  0.0001
condition5:degrees1                     38.25832  6.628732  24   5.77159  0.0000
condition1:minutes_cent                  2.37013  0.280450 546   8.45117  0.0000
condition2:minutes_cent                  0.29240  0.280450 546   1.04261  0.2976
condition3:minutes_cent                  0.94594  0.280450 546   3.37294  0.0008
condition4:minutes_cent                 -1.45232  0.280450 546  -5.17856  0.0000
condition5:minutes_cent                 -1.84435  0.280450 546  -6.57640  0.0000
degrees1:minutes_cent                    2.64947  0.125421 546  21.12461  0.0000
condition1:I(minutes_cent^2)             0.02996  0.002945 546  10.17484  0.0000
condition2:I(minutes_cent^2)             0.00310  0.002945 546   1.05403  0.2923
condition3:I(minutes_cent^2)             0.00982  0.002945 546   3.33318  0.0009
condition4:I(minutes_cent^2)            -0.01717  0.002945 546  -5.83166  0.0000
condition5:I(minutes_cent^2)            -0.02166  0.002945 546  -7.35376  0.0000
degrees1:I(minutes_cent^2)               0.03337  0.001317 546  25.33920  0.0000
condition1:degrees1:minutes_cent        -2.45849  0.280450 546  -8.76627  0.0000
condition2:degrees1:minutes_cent        -0.29997  0.280450 546  -1.06961  0.2853
condition3:degrees1:minutes_cent        -0.94543  0.280450 546  -3.37112  0.0008
condition4:degrees1:minutes_cent         1.52080  0.280450 546   5.42272  0.0000
condition5:degrees1:minutes_cent         1.90178  0.280450 546   6.78118  0.0000
condition1:degrees1:I(minutes_cent^2)   -0.02969  0.002945 546 -10.08249  0.0000
condition2:degrees1:I(minutes_cent^2)   -0.00284  0.002945 546  -0.96385  0.3355
condition3:degrees1:I(minutes_cent^2)   -0.00976  0.002945 546  -3.31303  0.0010
condition4:degrees1:I(minutes_cent^2)    0.01676  0.002945 546   5.69118  0.0000
```

```
condition5:degrees1:I(minutes_cent^2)    0.02143  0.002945 546   7.27605  0.0000
 Correlation: 
                                      (Intr) cndtn1 cndtn2 cndtn3 cndtn4 cndtn5 degrs1 mnts_c I(_^2) cnd1:1 cnd2:1 cnd3:1 cnd4:1 cnd5:1 cnd1:_ cnd2:_
condition1                             0.000                                                                                                         
condition2                             0.000 -0.200                                                                                                  
condition3                             0.000 -0.200 -0.200                                                                                           
condition4                             0.000 -0.200 -0.200 -0.200                                                                                    
condition5                             0.000 -0.200 -0.200 -0.200 -0.200                                                                             
degrees1                              -0.877  0.000  0.000  0.000  0.000  0.000                                                                      
minutes_cent                           0.893  0.000  0.000  0.000  0.000  0.000 -0.961                                                               
I(minutes_cent^2)                      0.866  0.000  0.000  0.000  0.000  0.000 -0.942  0.989                                                        
condition1:degrees1                    0.000 -0.944  0.189  0.189  0.189  0.189  0.000  0.000  0.000                                                 
condition2:degrees1                    0.000  0.189 -0.944  0.189  0.189  0.189  0.000  0.000  0.000 -0.200                                          
condition3:degrees1                    0.000  0.189  0.189 -0.944  0.189  0.189  0.000  0.000  0.000 -0.200 -0.200                                   
condition4:degrees1                    0.000  0.189  0.189  0.189 -0.944  0.189  0.000  0.000  0.000 -0.200 -0.200 -0.200                            
condition5:degrees1                    0.000  0.189  0.189  0.189  0.189 -0.944  0.000  0.000  0.000 -0.200 -0.200 -0.200 -0.200                     
condition1:minutes_cent                0.000  0.962 -0.192 -0.192 -0.192 -0.192  0.000  0.000  0.000 -0.961  0.192  0.192  0.192  0.192              
condition2:minutes_cent                0.000 -0.192  0.962 -0.192 -0.192 -0.192  0.000  0.000  0.000  0.192 -0.961  0.192  0.192  0.192 -0.200       
condition3:minutes_cent                0.000 -0.192 -0.192  0.962 -0.192 -0.192  0.000  0.000  0.000  0.192  0.192 -0.961  0.192  0.192 -0.200 -0.200
condition4:minutes_cent                0.000 -0.192 -0.192 -0.192  0.962 -0.192  0.000  0.000  0.000  0.192  0.192  0.192 -0.961  0.192 -0.200 -0.200
condition5:minutes_cent                0.000 -0.192 -0.192 -0.192 -0.192  0.962  0.000  0.000  0.000  0.192  0.192  0.192  0.192 -0.961 -0.200 -0.200
degrees1:minutes_cent                 -0.892  0.000  0.000  0.000  0.000  0.000  0.962 -0.997 -0.991  0.000  0.000  0.000  0.000  0.000  0.000  0.000
condition1:I(minutes_cent^2)           0.000  0.933 -0.187 -0.187 -0.187 -0.187  0.000  0.000  0.000 -0.942  0.188  0.188  0.188  0.188  0.989 -0.198
condition2:I(minutes_cent^2)           0.000 -0.187  0.933 -0.187 -0.187 -0.187  0.000  0.000  0.000  0.188 -0.942  0.188  0.188  0.188 -0.198  0.989
condition3:I(minutes_cent^2)           0.000 -0.187 -0.187  0.933 -0.187 -0.187  0.000  0.000  0.000  0.188  0.188 -0.942  0.188  0.188 -0.198 -0.198
condition4:I(minutes_cent^2)           0.000 -0.187 -0.187 -0.187  0.933 -0.187  0.000  0.000  0.000  0.188  0.188  0.188 -0.942  0.188 -0.198 -0.198
condition5:I(minutes_cent^2)           0.000 -0.187 -0.187 -0.187 -0.187  0.933  0.000  0.000  0.000  0.188  0.188  0.188  0.188 -0.942 -0.198 -0.198
degrees1:I(minutes_cent^2)            -0.875  0.000  0.000  0.000  0.000  0.000  0.933 -0.991 -0.994  0.000  0.000  0.000  0.000  0.000  0.000  0.000
condition1:degrees1:minutes_cent       0.000 -0.961  0.192  0.192  0.192  0.192  0.000  0.000  0.000  0.962 -0.192 -0.192 -0.192 -0.192 -0.997  0.199
condition2:degrees1:minutes_cent       0.000  0.192 -0.961  0.192  0.192  0.192  0.000  0.000  0.000 -0.192  0.962 -0.192 -0.192 -0.192  0.199 -0.997
condition3:degrees1:minutes_cent       0.000  0.192  0.192 -0.961  0.192  0.192  0.000  0.000  0.000 -0.192 -0.192  0.962 -0.192 -0.192  0.199  0.199
condition4:degrees1:minutes_cent       0.000  0.192  0.192  0.192 -0.961  0.192  0.000  0.000  0.000 -0.192 -0.192 -0.192  0.962 -0.192  0.199  0.199
condition5:degrees1:minutes_cent       0.000  0.192  0.192  0.192  0.192 -0.961  0.000  0.000  0.000 -0.192 -0.192 -0.192 -0.192  0.962  0.199  0.199
condition1:degrees1:I(minutes_cent^2)  0.000 -0.942  0.188  0.188  0.188  0.188  0.000  0.000  0.000  0.933 -0.187 -0.187 -0.187 -0.187 -0.991  0.198
condition2:degrees1:I(minutes_cent^2)  0.000  0.188 -0.942  0.188  0.188  0.188  0.000  0.000  0.000 -0.187  0.933 -0.187 -0.187 -0.187  0.198 -0.991
condition3:degrees1:I(minutes_cent^2)  0.000  0.188  0.188 -0.942  0.188  0.188  0.000  0.000  0.000 -0.187 -0.187  0.933 -0.187 -0.187  0.198  0.198
condition4:degrees1:I(minutes_cent^2)  0.000  0.188  0.188  0.188 -0.942  0.188  0.000  0.000  0.000 -0.187 -0.187 -0.187  0.933 -0.187  0.198  0.198
condition5:degrees1:I(minutes_cent^2)  0.000  0.188  0.188  0.188  0.188 -0.942  0.000  0.000  0.000 -0.187 -0.187 -0.187 -0.187  0.933  0.198  0.198
                                      cnd3:_ cnd4:_ cnd5:_ dgr1:_ c1:I(_ c2:I(_ c3:I(_ c4:I(_ c5:I(_ d1:I(_ c1:1:_ c2:1:_ c3:1:_ c4:1:_ c5:1:_ c1:1:I
condition1                                                                                                                                           
condition2                                                                                                                                           
condition3                                                                                                                                           
condition4                                                                                                                                           
condition5                                                                                                                                           
degrees1                                                                                                                                             
minutes_cent                                                                                                                                         
I(minutes_cent^2)                                                                                                                                    
condition1:degrees1                                                                                                                                  
```

```
condition2:degrees1
condition3:degrees1
condition4:degrees1
condition5:degrees1
condition1:minutes_cent
condition2:minutes_cent
condition3:minutes_cent
condition4:minutes_cent               -0.200
condition5:minutes_cent               -0.200 -0.200
degrees1:minutes_cent                  0.000  0.000  0.000
condition1:I(minutes_cent^2)          -0.198 -0.198 -0.198  0.000
condition2:I(minutes_cent^2)          -0.198 -0.198 -0.198  0.000 -0.200
condition3:I(minutes_cent^2)           0.989 -0.198 -0.198  0.000 -0.200 -0.200
condition4:I(minutes_cent^2)          -0.198  0.989 -0.198  0.000 -0.200 -0.200 -0.200
condition5:I(minutes_cent^2)          -0.198 -0.198  0.989  0.000 -0.200 -0.200 -0.200 -0.200
degrees1:I(minutes_cent^2)             0.000  0.000  0.000  0.989  0.000  0.000  0.000  0.000  0.000
condition1:degrees1:minutes_cent       0.199  0.199  0.199  0.000 -0.991  0.198  0.198  0.198  0.198  0.000
condition2:degrees1:minutes_cent       0.199  0.199  0.199  0.000  0.198 -0.991  0.198  0.198  0.198  0.000 -0.200
condition3:degrees1:minutes_cent      -0.997  0.199  0.199  0.000  0.198  0.198 -0.991  0.198  0.198  0.000 -0.200 -0.200
condition4:degrees1:minutes_cent       0.199 -0.997  0.199  0.000  0.198  0.198  0.198 -0.991  0.198  0.000 -0.200 -0.200 -0.200
condition5:degrees1:minutes_cent       0.199  0.199 -0.997  0.000  0.198  0.198  0.198  0.198 -0.991  0.000 -0.200 -0.200 -0.200 -0.200
condition1:degrees1:I(minutes_cent^2)  0.198  0.198  0.198  0.000 -0.994  0.199  0.199  0.199  0.199  0.000  0.989 -0.198 -0.198 -0.198 -0.198
condition2:degrees1:I(minutes_cent^2)  0.198  0.198  0.198  0.000  0.199 -0.994  0.199  0.199  0.199  0.000 -0.198  0.989 -0.198 -0.198 -0.198 -0.200
condition3:degrees1:I(minutes_cent^2) -0.991  0.198  0.198  0.000  0.199  0.199 -0.994  0.199  0.199  0.000 -0.198 -0.198  0.989 -0.198 -0.198 -0.200
condition4:degrees1:I(minutes_cent^2)  0.198 -0.991  0.198  0.000  0.199  0.199  0.199 -0.994  0.199  0.000 -0.198 -0.198 -0.198  0.989 -0.198 -0.200
condition5:degrees1:I(minutes_cent^2)  0.198  0.198 -0.991  0.000  0.199  0.199  0.199  0.199 -0.994  0.000 -0.198 -0.198 -0.198 -0.198  0.989 -0.200
                                      c2:1:I c3:1:I c4:1:I
condition1
condition2
condition3
condition4
condition5
degrees1
minutes_cent
I(minutes_cent^2)
condition1:degrees1
condition2:degrees1
condition3:degrees1
condition4:degrees1
condition5:degrees1
condition1:minutes_cent
condition2:minutes_cent
condition3:minutes_cent
condition4:minutes_cent
condition5:minutes_cent
degrees1:minutes_cent
condition1:I(minutes_cent^2)
condition2:I(minutes_cent^2)
```

```
condition3:I(minutes_cent^2)
condition4:I(minutes_cent^2)
condition5:I(minutes_cent^2)
degrees1:I(minutes_cent^2)
condition1:degrees1:minutes_cent
condition2:degrees1:minutes_cent
condition3:degrees1:minutes_cent
condition4:degrees1:minutes_cent
condition5:degrees1:minutes_cent
condition1:degrees1:I(minutes_cent^2)
condition2:degrees1:I(minutes_cent^2)
condition3:degrees1:I(minutes_cent^2) -0.200
condition4:degrees1:I(minutes_cent^2) -0.200 -0.200
condition5:degrees1:I(minutes_cent^2) -0.200 -0.200 -0.200

Standardized Within-Group Residuals:
        Min          Q1         Med          Q3         Max
-2.97777854 -0.51702088 -0.01250641  0.44773568  2.75112919

Number of Observations: 630
Number of Groups:
                                virus_batch                          date %in% virus_batch           test_tube %in% date %in% virus_batch
                                          2                                              5                                             30
unit %in% test_tube %in% date %in% virus_batch
                                          60
```

4.5 Model Diagnostic Plots

Diagnostic plots for the primary (complex) model, the simple comparison model, and the two intermediate-stage models are shown below.

4.5.1 Model with Four Random Effects and Variance and Correlation Structures

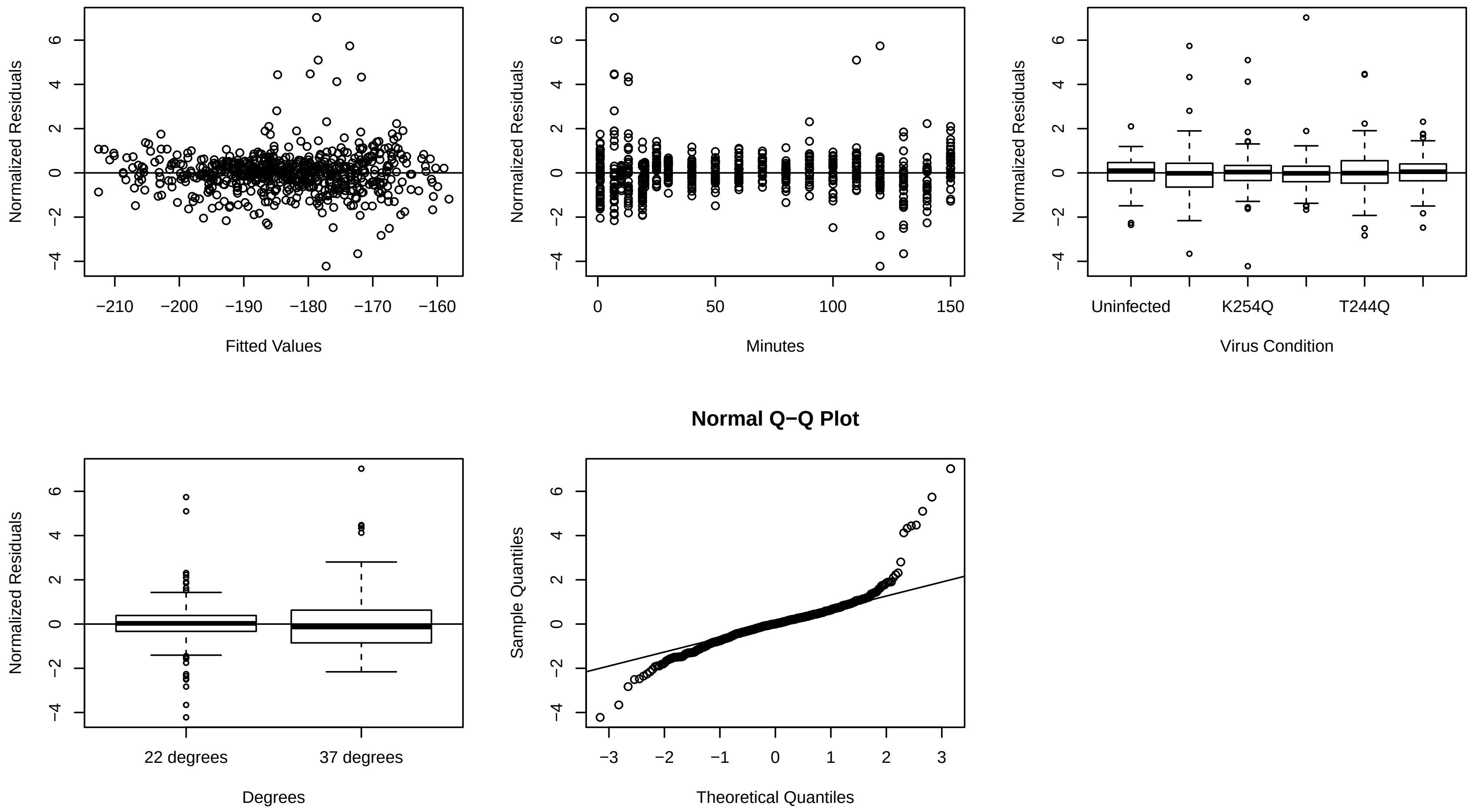

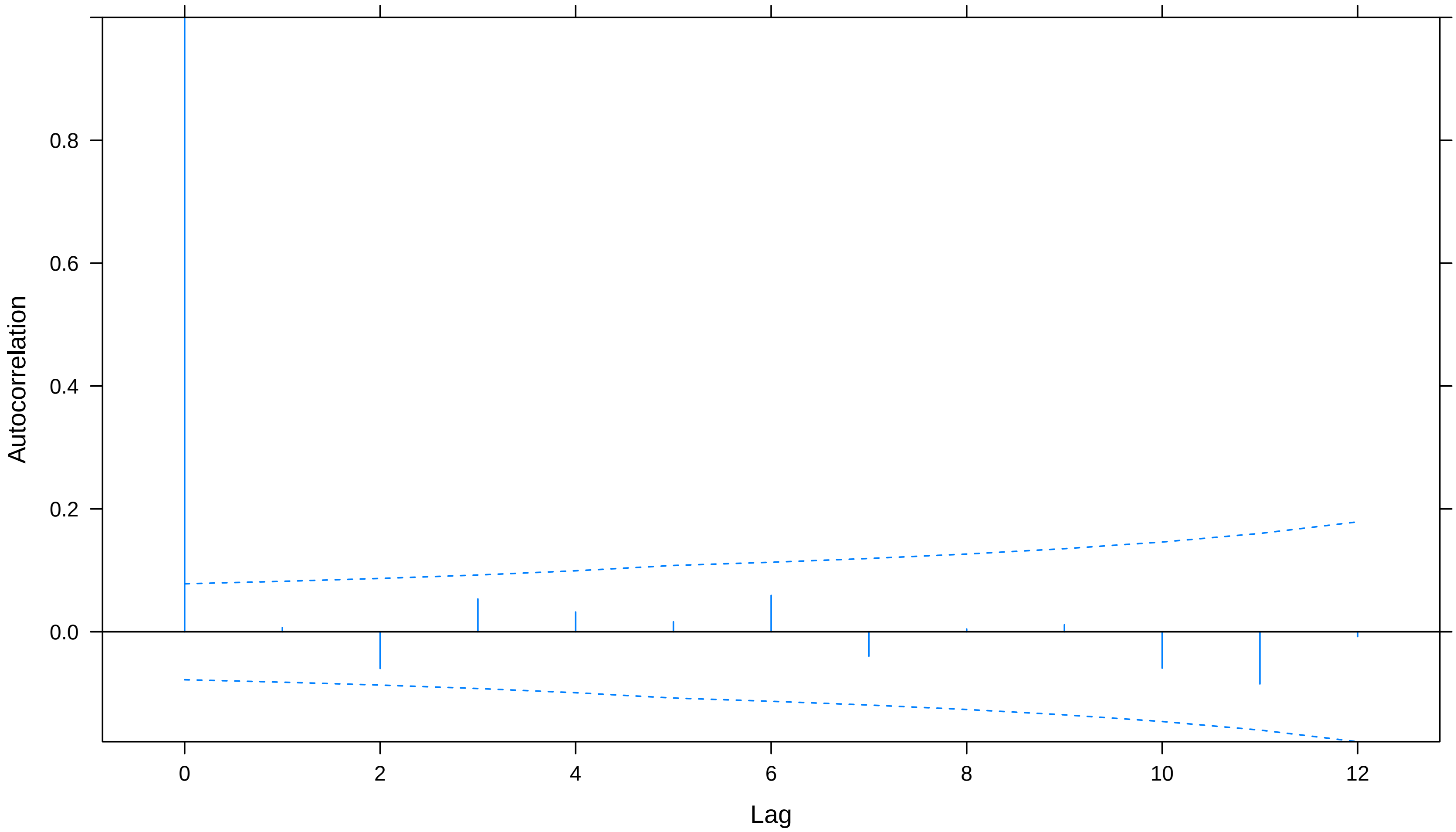

0.8
0.6
0.4
0.2
0.0
Autocorrelation
0
2
4
6
8
10
12
Lag

4.5.2 Model with Two Random Effects (no Variance or Correlation Structures)

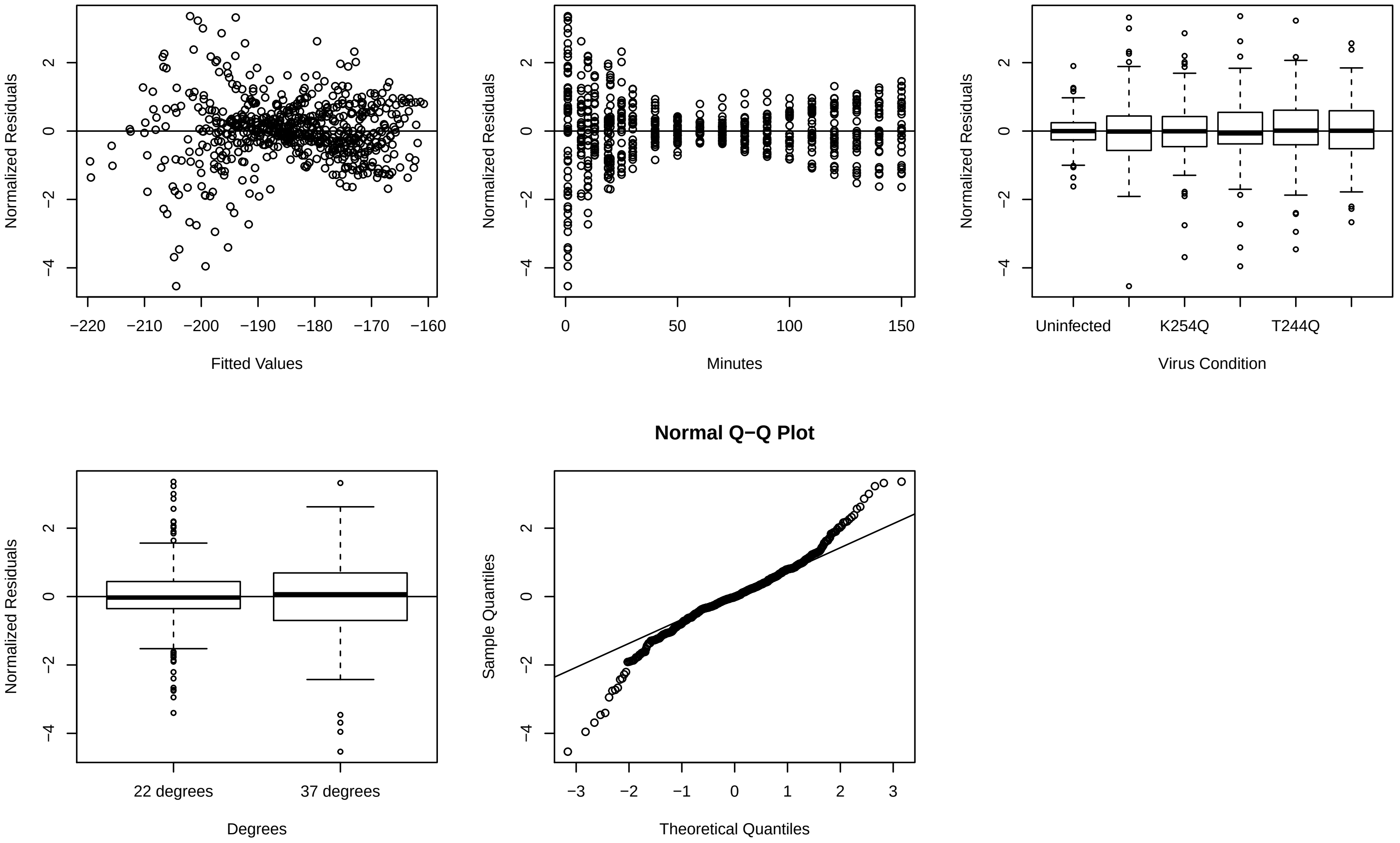

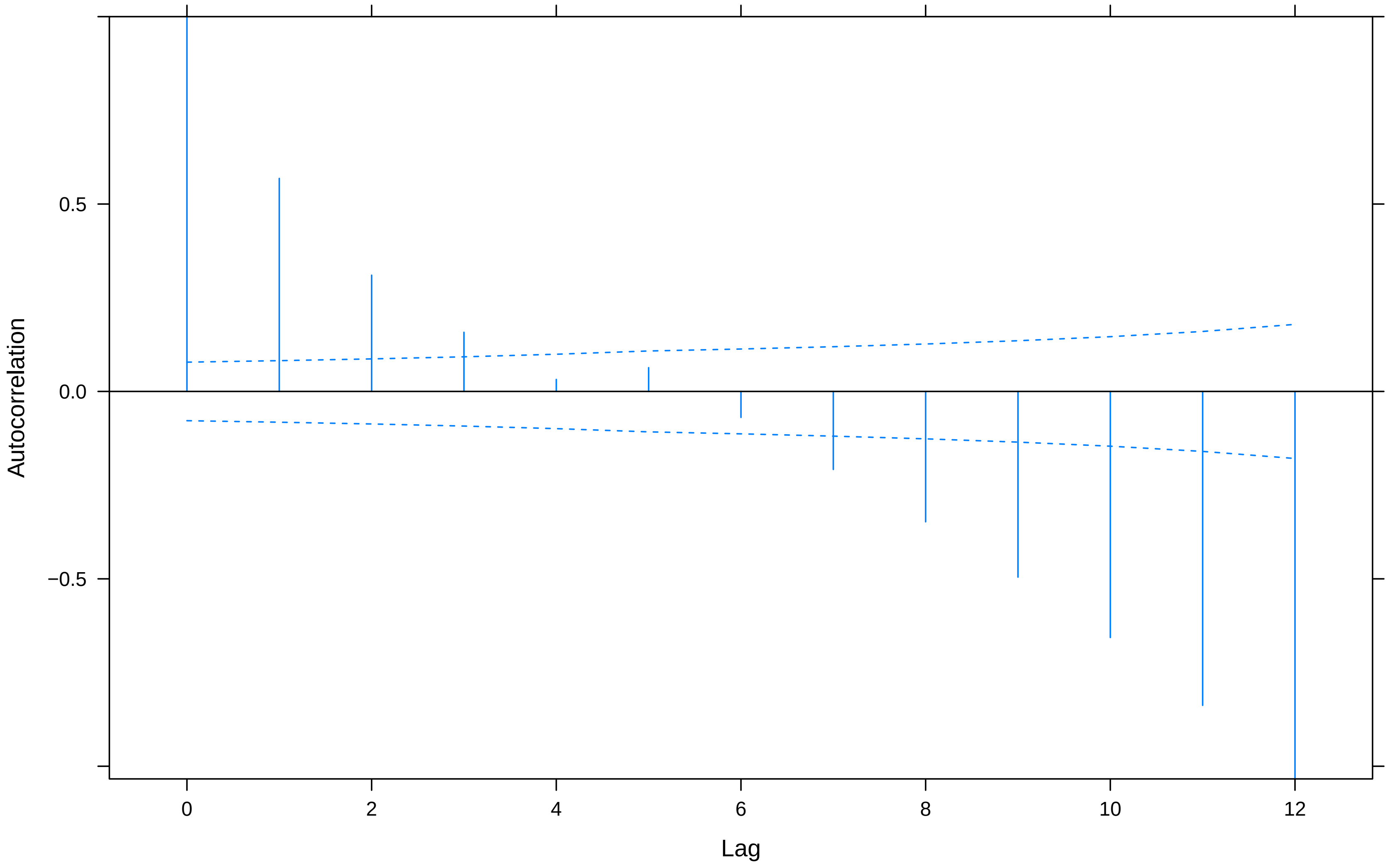

Autocorrelation
0.5
0.0
−0.5
0
2
4
6
8
10
12
Lag

4.5.3 Model with Four Random Effects (no Variance or Correlation Structures)

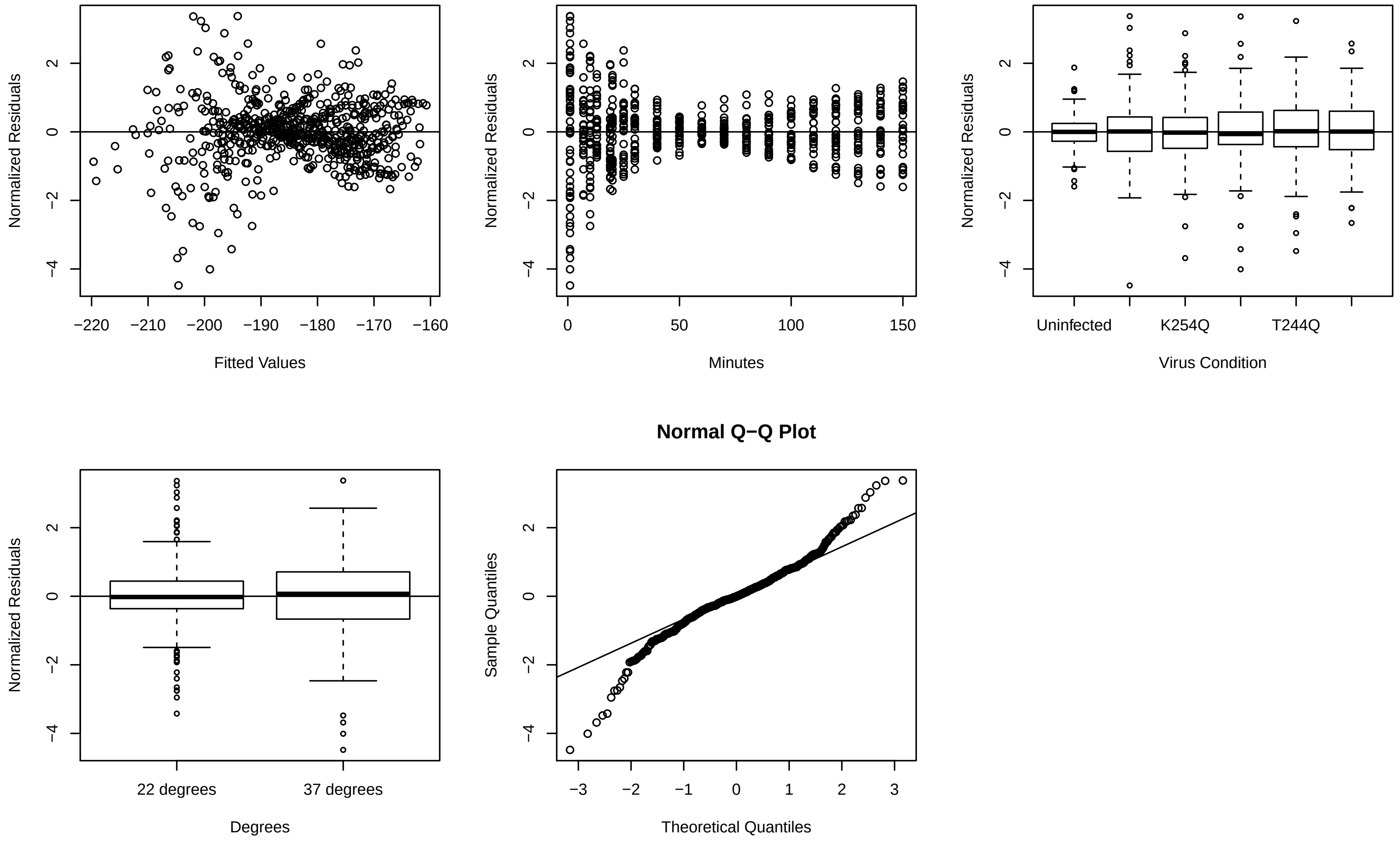

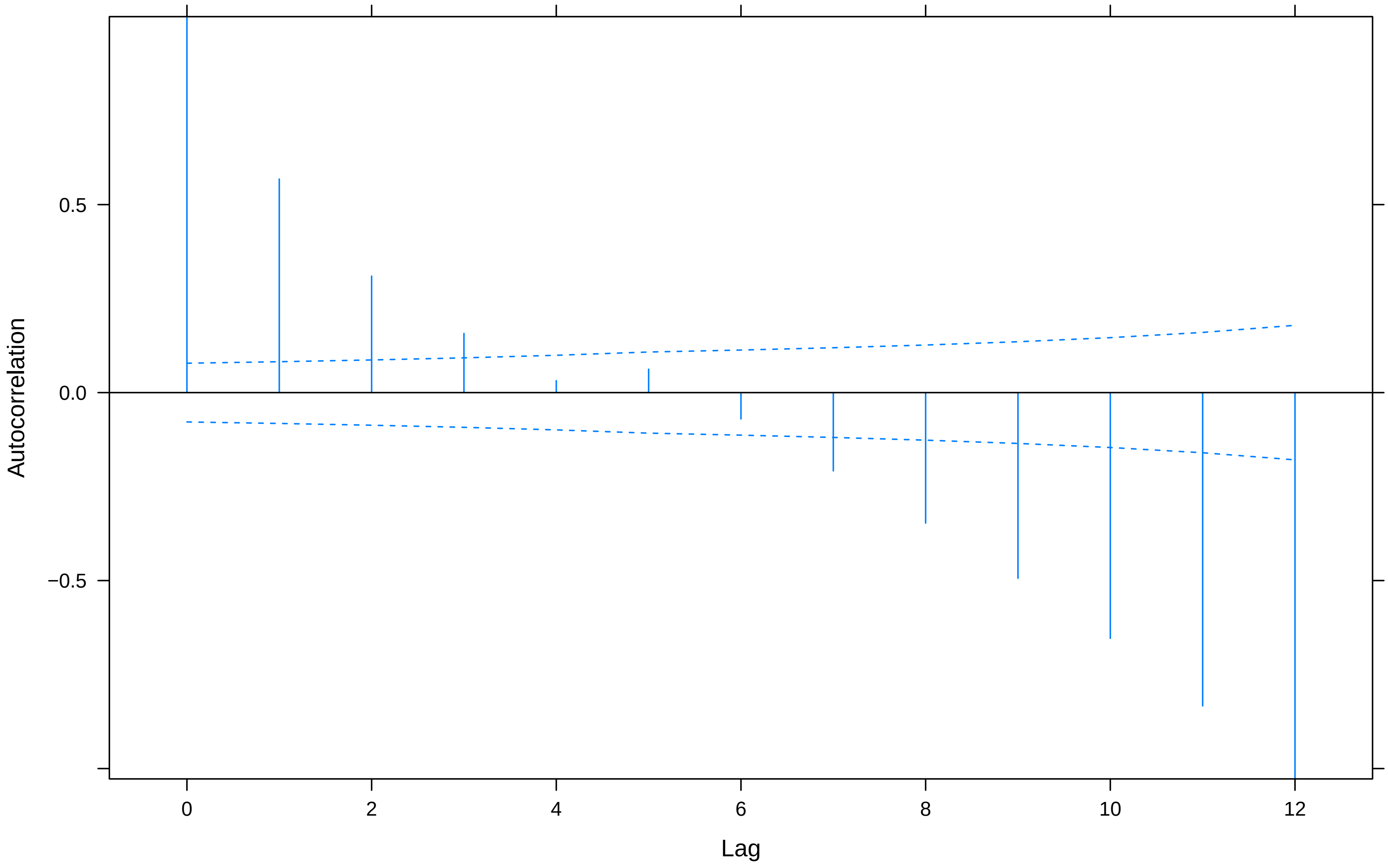

Autocorrelation
0.5
0.0
−0.5
0
2
4
6
8
10
12
Lag

4.5.4 Model with Four Random Effects and a Correlation Structure (no Variance Structure)

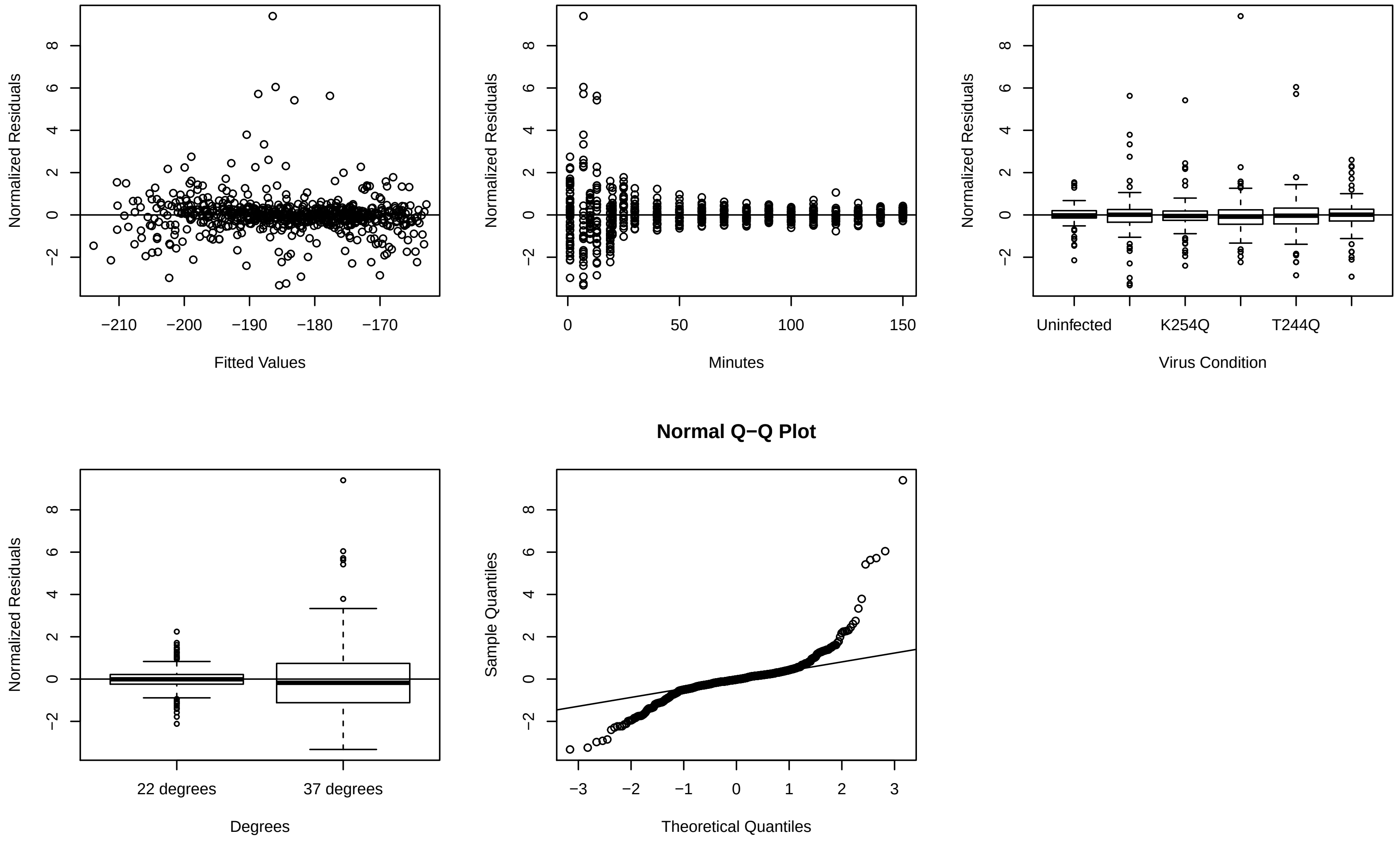

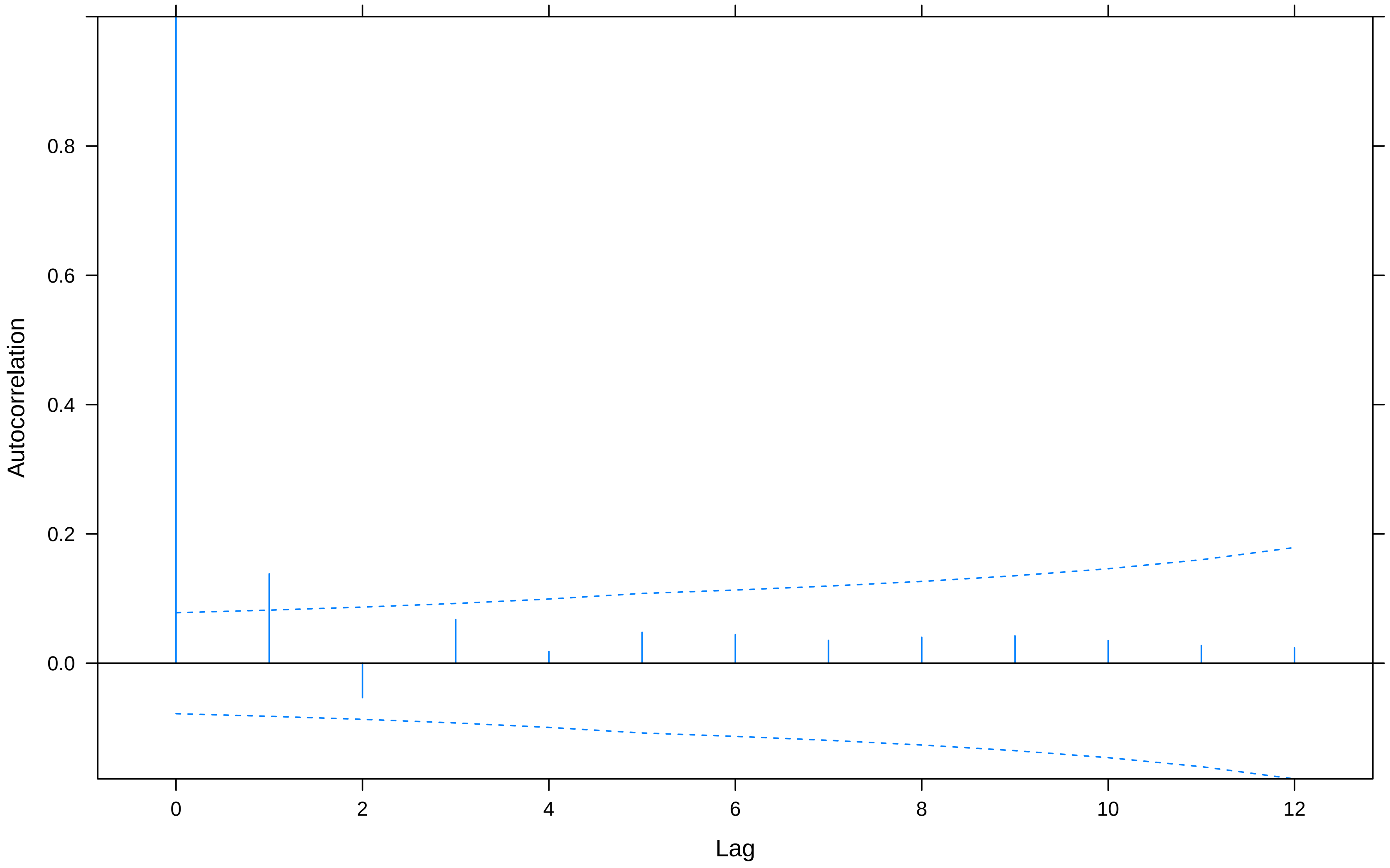

Autocorrelation
0.0
0.2
0.4
0.6
0.8
0
2
4
6
8
10
12
Lag

4.6 Code

All code that went into this document is printed below.

```
# Code Chunk: chunk_options

# This is where we set basic knitr options.
opts_chunk$set(echo=FALSE, message=FALSE, warning = FALSE, cache = FALSE, comment="")
options(width=150) # This sets how wide the R printout can be.
options(contrasts=c("contr.sum", "contr.poly")) # I'm getting weird degrees of freedom from treatment contrasts probably because of thow they treat the intercept degrees of freedom.

# Code Chunk: load_libraries

dyn.load('/Library/Java/JavaVirtualMachines/jdk1.8.0_40.jdk/Contents/Home/jre/lib/server/libjvm.dylib')
library(tidyr)
library(dplyr)
library(ggplot2)
library(lme4)
library(lsmeans)
library(car)
library(pbkrtest)
library(xtable)
library(xlsx)
library(nlme)

# Code Chunk: data_initialization

# Julia home:
# base.path <- "/Users/Julia/Documents/StatsLab_UA/projects/Roznowski/KplusEffluxAssay/data/"
# Julia work:
base.path <- "/Users/jmfisher/Documents/Projects/Roznowski/KplusEffluxAssay/data/"
dataset.file22 <- paste0(base.path, "PhiX174RawPotassiumEfflux22Degrees.csv")
dataset.file37 <- paste0(base.path, "PhiX174RawPotassiumEfflux37Degrees.csv") # I had to change the last column name in this file from T244Q.M251QNov7 to T244Q.M251QNov07.  It was just an added 0.  Now, the column names match between the two files.
dta22 <- read.csv(dataset.file22) %>%
  mutate(degrees = "22 degrees")
dta37 <- read.csv(dataset.file37) %>%
  mutate(degrees = "37 degrees")
dta <- rbind(dta22, dta37) %>%
  gather(key, value, 2:(ncol(.) - 1)) %>%
  mutate(condition = factor(gsub("(Oct17)|(Oct23)|(Oct24)|(Oct30)|(Nov07)", "", key),
                            levels = c("Uninfected", "WildType", "K254Q", "M251Q", "T244Q", "T244Q.M251Q")),
         date = factor(gsub(paste0("(", paste0(unique(condition), collapse = ")|("), ")"), "", key), levels = c("Oct17", "Oct23", "Oct24", "Oct30", "Nov07")),
         virus_batch = ifelse(date %in% c("Oct17", "Oct23", "Oct24", "Oct30"), "1", "2"),
         test_tube = as.character(as.numeric(factor(key))),
         unit = as.character(as.numeric(factor(paste0(key, degrees)))),
         minutes_fac = factor(as.character(Minutes), levels = as.character(sort(unique(.$Minutes)))),
         minutes_cent = Minutes - mean(.$Minutes),
         minutes_cent_sq = minutes_cent^2,
         degrees = as.factor(degrees)) %>%
  rename(minutes = Minutes,
         mV = value) %>%
  dplyr::select(date, virus_batch, test_tube, unit, condition, degrees, minutes, minutes_fac, minutes_cent, minutes_cent_sq, mV)

dta22 <- dta %>%
  filter(degrees == "22 degrees") %>%
  mutate(minutes_cent = minutes - mean(.$minutes),
         minutes_cent_sq = minutes_cent^2)

dta37 <- dta %>%
  filter(degrees == "37 degrees") %>%
  mutate(minutes_cent = minutes - mean(.$minutes),
         minutes_cent_sq = minutes_cent^2)


# Code Chunk: initial_plots

ggplot(dta[which(dta$degrees == "22 degrees"), ],
       aes(x = minutes, y = mV, colour = condition, fill = condition)) +
  facet_grid(degrees ~ date) +
  geom_line()

ggplot(dta[which(dta$degrees == "37 degrees"), ],
       aes(x = minutes, y = mV, colour = condition, fill = condition)) +
```

```r
  facet_grid(degrees ~ date) +
  geom_line()




# Code Chunk: combined_22_37_models_quadratic

# Models without variance and correlation structures:

# A version of mdl1 but with only random effects for unit and day:
mdl1.basicrand <- lme(mV ~ condition*degrees*minutes_cent + condition*degrees*I(minutes_cent^2),
                      random = ~ 1 | date/unit,
                      data = dta)
acf.mdl1.basicrand <- plot(ACF(mdl1.basicrand), alpha = 0.05)

# This is the complete 'basic' model (random effects but no variance or correlation structure):
mdl1 <- lme(mV ~ condition*degrees*minutes_cent + condition*degrees*I(minutes_cent^2),
            random = ~ 1 | virus_batch/date/test_tube/unit,
            data = dta)
acf.mdl1 <- plot(ACF(mdl1), alpha = 0.05)

###############################################

# Adding Correlation Structures:

# Let's try an exponential correlation model:
mdl1.corexp <- update(mdl1, correlation = corExp(form = ~ minutes_cent, nugget = TRUE))
acf.mdl1.corexp <- plot(ACF(mdl1.corexp, resType = "n"), alpha = 0.05) # Probably still a bit worse than acf.mdl1.car1.
# anova(mdl1, mdl1.corexp) # difference

###############################################

# Adding Variance Structures:

# Now, let's see if using the exponential correlation structure and exponential variance structure is better.
mdl1.corexp.varexp <- update(mdl1.corexp, weights = varExp(form = ~ minutes))
acf.mdl1.corexp.varexp <- plot(ACF(mdl1.corexp.varexp, resType = "n"), alpha = 0.05)
# anova(mdl1.corexp, mdl1.corexp.varexp) # difference

# Just check for basic differences between the no-correlation structure, no-variance structure
# models and mdl1.corexp.varexp.
# anova(mdl1, mdl1.corexp.varexp) # difference
# anova(mdl1.basicrand, mdl1.corexp.varexp) # difference


# Code Chunk:  other_reasonable_quadratic_combined_models

# I explored a wide range of models while considering this analysis.
# I went through these models and compared them to the model with an
# exponential correlation and variance structure.  I found these to
# not perform as well as indicated by autocorrelation functions.

# Other reasonable explored models:

# A version of mdl1 but with fixed effects.
# We (unsurprisingly) get a lot of non-estimable stuff.
mdl1.fixed <- lm(mV ~ condition*degrees*minutes_cent + condition*degrees*I(minutes_cent^2) +
                   unit,
                 data = dta)

# Let's try a continuous AR(1) model:
mdl1.car1 <- update(mdl1, correlation = corCAR1(form = ~ minutes_cent))
acf.mdl1.car1 <- plot(ACF(mdl1.car1, resType = "n"), alpha = 0.05)
plot(residuals(mdl1.car1, type = "normalized") ~ fitted(mdl1.car1))
abline(h = 0)
plot(residuals(mdl1.car1, type = "normalized") ~ dta$minutes)
abline(h = 0) # heteroscedasticity
anova(mdl1, mdl1.car1) # difference

# Now, let's model the variance within group as an exponential function of time.
mdl1.car1.varexp <- update(mdl1.car1, weights = varExp(form = ~ minutes))
acf.mdl1.car1.varexp <- plot(ACF(mdl1.car1.varexp, resType = "n"), alpha = 0.05)
plot(residuals(mdl1.car1.varexp, type = "normalized") ~ fitted(mdl1.car1.varexp))
abline(h = 0)
plot(residuals(mdl1.car1.varexp, type = "normalized") ~ dta$minutes_cent)
abline(h = 0)
```

```r
qqnorm(mdl1.car1.varexp, ~ resid(., type = "n"))
anova(mdl1.car1, mdl1.car1.varexp) # difference

# Now, let's see if modeling the variance within group as an exponential function of time
# and stratifying by degrees helps.
mdl1.car1.varexps <- update(mdl1.car1, weights = varExp(form = ~ minutes | degrees))
acf.mdl1.car1.varexps <- plot(ACF(mdl1.car1.varexps, resType = "n"), alpha = 0.05)
plot(residuals(mdl1.car1.varexps, type = "normalized") ~ fitted(mdl1.car1.varexps))
abline(h = 0)
plot(residuals(mdl1.car1.varexps, type = "normalized") ~ dta$minutes_cent)
abline(h = 0)
anova(mdl1.car1.varexp, mdl1.car1.varexps) # no difference from the unstratified

# Let's try an ARMA(1,1) model:
mdl1.arma11 <- update(mdl1, correlation = corARMA(p = 1, q = 1))
acf.mdl1.arma11 <- plot(ACF(mdl1.arma11, resType = "n"), alpha = 0.05) # Clearly worse.

# Now, let's see if modeling the variance within group as an exponential function of time
# and stratifying by degrees helps.
mdl1.corexp.varexps <- update(mdl1.corexp, weights = varExp(form = ~ minutes | degrees))
acf.mdl1.corexp.varexps <- plot(ACF(mdl1.corexp.varexps, resType = "n"), alpha = 0.05)
plot(residuals(mdl1.corexp.varexps, type = "normalized") ~ fitted(mdl1.corexp.varexps))
abline(h = 0)
plot(residuals(mdl1.corexp.varexps, type = "normalized") ~ dta$minutes_cent)
abline(h = 0)
anova(mdl1.corexp.varexp, mdl1.corexp.varexps) # yes difference from unstratified, but the autocorrelation increases.

# Now, let's see if modeling the variance within group as an exponential function of time
# and stratifying by degrees AND conditions helps.
mdl1.corexp.varexps2 <- update(mdl1.corexp, weights = varExp(form = ~ minutes | degrees*condition)) # won't converge
acf.mdl1.corexp.varexps2 <- plot(ACF(mdl1.corexp.varexps2, resType = "n"), alpha = 0.05)
plot(residuals(mdl1.corexp.varexps2, type = "normalized") ~ fitted(mdl1.corexp.varexps))
abline(h = 0)
plot(residuals(mdl1.corexp.varexps2, type = "normalized") ~ dta$minutes_cent)
abline(h = 0)
anova(mdl1.corexp.varexp, mdl1.corexp.varexps2)

# I'm not evaluating this code chunk because I don't plan on printing out any of the results.


# Code Chunk: combined_22_37_models_quartic

# I was just curious about whether or not mdl1 (the generic, pre-variance or correlation components model)
# would benefit from cubic or quartic effects of time.
mdl1.cubic <- lme(mV ~ condition*degrees*(minutes_cent + I(minutes_cent^2) + I(minutes_cent^3)),
                    random = ~ 1 | virus_batch/date/test_tube/unit,
                    data = dta)
plot(residuals(mdl1.cubic, type = "pearson") ~ fitted(mdl1.cubic))
abline(h = 0)
plot(resid(mdl1.cubic, type = "pearson") ~ dta$minutes)
abline(h = 0)
anova(update(mdl1, method = "ML"), update(mdl1.cubic, method = "ML")) # Not significantly different

mdl1.quartic <- lme(mV ~ condition*degrees*(minutes_cent + I(minutes_cent^2) + I(minutes_cent^3) + I(minutes_cent^4)),
                    random = ~ 1 | virus_batch/date/test_tube/unit,
                    data = dta)
plot(residuals(mdl1.quartic, type = "pearson") ~ fitted(mdl1.quartic))
abline(h = 0)
plot(resid(mdl1.quartic, type = "pearson") ~ dta$minutes)
abline(h = 0)
anova(update(mdl1, method = "ML"), update(mdl1.quartic, method = "ML")) # Not significantly different

# Basically, the above tells me that we don't need more than quadratic effects of time.
# With that in mind, I'll stick with the quadratic models.
# Also, I stopped evaluating this chunk because I don't need it when I'm printing out the document for Aaron.


# Code Chunk:  separate_22_models

# I briefly considered separating the data into separate temperature groups
# and running separate models on each dataset.  I ran into some model fitting
# issues and bagged this idea.

mdl1.22 <- lme(mV ~ condition*minutes_cent + condition*minutes_cent_sq, random = ~ 1 | virus_batch/date/test_tube,
             data = dta22)
plot(residuals(mdl1.22, type = "pearson") ~ fitted(mdl1.22))
abline(h = 0)
```

```
plot(resid(mdl1.22, type = "pearson") ~ dta22$minutes)
abline(h = 0)
acf1.22 <- plot(ACF(mdl1.22), alpha = 0.05)
plot(Variogram(mdl1.22, form = ~ minutes))

# This doesn't work...
# Error in logLik.reStruct(object, conLin) :
#   NA/NaN/Inf in foreign function call (arg 3)
# mdl2.22 <- update(mdl1.22, correlation = corCAR1(form = ~ minutes_cent))

# Try a basic AR(1) model:
# This didn't work either.
# mdl2.22 <- update(mdl1.22, correlation = corAR1())

# Try a Exponential decay on the correlation structure:
mdl2.22 <- update(mdl1.22, correlation = corExp(form = ~ minutes_cent, nugget = TRUE)) # Yeah!  This worked.
plot(residuals(mdl2.22, type = "pearson") ~ fitted(mdl2.22))
abline(h = 0)
plot(resid(mdl2.22, type = "pearson") ~ dta22$minutes)
abline(h = 0)
acf2.22 <- plot(ACF(mdl2.22), alpha = 0.05)


# Other useful model bits:
corexp <- corExp(form = ~ minutes | unit, nugget = TRUE)
corexp <- Initialize(corexp, data = dta)
as.matrix(corexp) # the initial correlation matrices for each innermost level of grouping (60 groups here)
# By default, the range is initialized to 0.9*minimum distance (which is 6) and
# nugget is initialized to 0.1.
coef(corexp) # the final coefficients for the correlation structure.
# So, with this in mind, I can create the final correlation matrices.
my.expcor <- function(dist, nugget, range) {
  if (class(dist) == "matrix") {
    out <- (1 - nugget)*exp(-dist/range)
    diag(out) <- 1
    out[which(is.na(out))] <- 0
    return(out)
  } else {
    if (dist == 0) {
      return(1)
    } else {
      return(as.numeric((1 - nugget)*exp(-dist/range)))
    }
  }
}
# Compare:
my.expcor(6, 0.1, 6*0.9)
my.expcor(6, coef(corexp)['nugget'], coef(corexp)['range'])
# So, I could use the above to generate the correlation matrices.  This is the C_i in Lambda_i

# Get the coefficients for the exponential variance function:
expvar <- varExp(form = ~ minutes)
coef(expvar) # Hmmm... This isn't useful to me.

# Try this out:
getData(mdl1.corexp.varexp) # This just pulls out the dataframe.
getVarCov(mdl1.corexp.varexp) # This doesn't work for the multiple levels of nesting.  Drat.


# I am uncertain about whether or not lsmeans is doing the correct thing when estimating confidence intervals and contrasts.
# I want to test this by hand to the best of my ability.
vcovfixed <- vcov(mdl1.corexp.varexp)
my.fixef <-fixef(mdl1.corexp.varexp)
my.df <- data.frame(num = 1:36, fixef = my.fixef)
# Wildtype at 25 minutes and 37 degrees
contrast <- rep(0, 36)
contrast[c(1, 3)] <- 1
contrast[c(7, 11)] <- -1
contrast[c(8, 16)] <- 25 - mean(dta$minutes)
contrast[c(20, 28)] <- -(25 - mean(dta$minutes))
contrast[c(9, 22)] <- (25 - mean(dta$minutes))^2
contrast[c(26, 33)] <- -(25 - mean(dta$minutes))^2
se <- sqrt(t(contrast) %*% vcovfixed %*% contrast)
contrast.est <- my.fixef %*% contrast
lb <- contrast.est - se*qnorm(0.975, lower.tail = TRUE)
ub <- contrast.est + se*qnorm(0.975, lower.tail = TRUE)
# These are not what lsmeans is giving me.  The estimate and the se are the same, but the confidence intervals are *totally* off.  So weird.
```

```r
# Ok.  I want to estimate the vcovfixed.  To do this, I need a bunch of block diagonal matrices where
# each block corresponds to a group.  Multiply these by the appropriate random effect variance estimate.
X <- as.matrix(model.matrix(mdl1.corexp.varexp, data = dta))
Z.virus <- model.matrix( ~ virus_batch - 1, data = dta)
Z.date <- model.matrix( ~ date - 1, data = dta)
Z.testtube <- model.matrix( ~ test_tube, data = dta)
Z.unit <- model.matrix( ~ unit, data = dta)

# Estimated variances:
estvar.virus <- as.numeric(VarCorr(mdl1.corexp.varexp)[2, 1])
estvar.date <- as.numeric(VarCorr(mdl1.corexp.varexp)[4, 1])
estvar.testtube <- as.numeric(VarCorr(mdl1.corexp.varexp)[6, 1])
estvar.unit <- as.numeric(VarCorr(mdl1.corexp.varexp)[8, 1])
sigma.sq <- as.numeric(VarCorr(mdl1.corexp.varexp)[9, 1])

# Z(var)Z^T
rand.virus.mat <- estvar.virus*(Z.virus %*% t(Z.virus))
rand.date.mat <- estvar.date*(Z.date %*% t(Z.date))
rand.testtube.mat <- estvar.testtube*(Z.testtube %*% t(Z.testtube))
rand.unit.mat <- estvar.unit*(Z.unit %*% t(Z.unit))

# Sum of Z(var)Z^T
rand.component <- rand.virus.mat + rand.date.mat + rand.testtube.mat + rand.unit.mat

# Now get the correlation matrices:
nugget <- coef(mdl1.corexp.varexp$modelStruct$corStruct, unconstrained = FALSE)['nugget']
range <- coef(mdl1.corexp.varexp$modelStruct$corStruct, unconstrained = FALSE)['range']
distances <- attr(mdl1.corexp.varexp$modelStruct$corStruct, 'covariate')

# Variance structure parameter:
cor.mat <- matrix(NA, nrow = nrow(dta), ncol = nrow(dta))
for (i in 1:nrow(dta)) {
 for (j in 1:nrow(dta)) {
   if (dta[i, 'unit'] == dta[j, 'unit']) {
   dist <- abs(dta[i, 'minutes'] - dta[j, 'minutes'])
   cor.mat[i, j] <- dist
   }
 }
}
cor.mat2 <- my.expcor(cor.mat, nugget, range)

# Now get the standard deviation matrices V.
# Each diagonal is exp(delta*minutes)
delta <- as.numeric(coef(mdl1.corexp.varexp$modelStruct$varStruct, unconstrained = FALSE))
sds <- exp(delta*dta$minutes)
sd.mat <- matrix(0, nrow = nrow(dta), ncol = nrow(dta))
diag(sd.mat) <- sds

var.y.mat <- sigma.sq*(rand.component + sd.mat %*% cor.mat2 %*% sd.mat)
var.y.mat.inv <- solve(var.y.mat)
var.beta.mat.tmp <- t(X) %*% var.y.mat.inv %*% X
var.beta.mat <- solve(var.beta.mat.tmp) # Okay.  This should be the variance covariance matrix of the beta hats. Compare to vcovfixed.

var.y.mat <- rand.component + sigma.sq*(sd.mat %*% cor.mat2 %*% sd.mat)
var.y.mat.inv <- solve(var.y.mat)
var.beta.mat.tmp <- t(X) %*% var.y.mat.inv %*% X
var.beta.mat <- solve(var.beta.mat.tmp)

# From this, we see 9 likely meaningful differences.
head(sort(as.numeric(vcovfixed - var.beta.mat)), 10)
tail(sort(as.numeric(vcovfixed - var.beta.mat)), 10)

diff <- vcovfixed - var.beta.mat
diff2 <- abs(diff) > 0.001
sum(as.numeric(diff2)) # 9
which(diff2 == T)
colnames(diff)

# (Intercept)
# conditionK254Q
# conditionK254Q:degrees37 degrees

# Code Chunk:  lsmeans

# Get estimated values at few time points.  Plot those estimated curves over the data.
# Print out 95% confidence intervals at each point.  Maybe make some kind of multiple comparisons correction.
```

```
mdl1.corexp.varexp.lsmeans <- lsmeans(mdl1.corexp.varexp, ~ condition*degrees*minutes_cent,
                                      at = list(minutes_cent = c(unique(dta$minutes_cent))))
mdl1.corexp.varexp.lsmeans.df <- summary(mdl1.corexp.varexp.lsmeans) %>%
  filter((degrees == "22 degrees" & minutes_cent %in% unique(dta[which(dta$degrees == "22 degrees") , 'minutes_cent'])) |
           (degrees == "37 degrees" & minutes_cent %in% unique(dta[which(dta$degrees == "37 degrees") , 'minutes_cent']))) %>%
  mutate(lower.CL.24df = lsmean - qt(0.975, df = 24, lower.tail = T)*SE,
         upper.CL.24df = lsmean + qt(0.975, df = 24, lower.tail = T)*SE)

# Plot
ggplot(mdl1.corexp.varexp.lsmeans.df,
       aes(x = minutes_cent + mean(dta$minutes), y = lsmean, fill = condition)) +
  facet_grid(. ~ degrees, scales = "free") +
  geom_ribbon(aes(ymin = lower.CL.24df, ymax = upper.CL.24df), alpha = 0.1) +
  geom_line(aes(colour = condition)) +
  theme(legend.position = 'bottom') +
  labs(x = "Minutes",
       y = "Model-Estimated Means",
       title = "Model-Estimated Means and Pointwise 95% Confidence Intervals") +
  theme(plot.title = element_text(hjust = 0.5))


# Test difference of wildtype and mutants at time = 25 minutes
mdl1.corexp.varexp.lsmeans.for.contrasts <- lsmeans(mdl1.corexp.varexp, ~ condition*degrees*minutes_cent,
                          at = list(minutes_cent = c(25 - mean(dta$minutes),
                                                     150 - mean(dta$minutes))))

mdl1.corexp.varexp.contrasts.of.interest <- summary(contrast(mdl1.corexp.varexp.lsmeans.for.contrasts,
                                                             list('K254Q vs. WildType at 25 minutes in 37 degrees' = c(rep(0, 6), 0, -1, 1, rep(0, 3), rep(0, 12)),
                                                                  'M251Q vs. WildType at 25 minutes in 37 degrees' = c(rep(0, 6), 0, -1, 0, 1, rep(0, 2), rep(0, 12)),
                                                                  'T244Q vs. WildType at 25 minutes in 37 degrees' = c(rep(0, 6), 0, -1, 0, 0, 1, 0, rep(0, 12)),
                                                                  'T244Q.M251Q vs. WildType at 25 minutes in 37 degrees' = c(rep(0, 6), 0, -1, 0, 0, 0, 1, rep(0, 12)),
                                                                  'K254Q vs. Uninfected at 25 minutes in 37 degrees' = c(rep(0, 6), -1, 0, 1, rep(0, 3), rep(0, 12)),
                                                                  'M251Q vs. Uninfected at 25 minutes in 37 degrees' = c(rep(0, 6), -1, 0, 0, 1, rep(0, 2), rep(0, 12)),
                                                                  'T244Q vs. Uninfected at 25 minutes in 37 degrees' = c(rep(0, 6), -1, 0, 0, 0, 1, 0, rep(0, 12)),
                                                                  'T244Q.M251Q vs. Uninfected at 25 minutes in 37 degrees' = c(rep(0, 6), -1, 0, 0, 0, 0, 1, rep(0, 12)),
                                                                  'K254Q vs. WildType at 150 minutes in 22 degrees' = c(rep(0, 12), 0, -1, 1, rep(0, 3), rep(0, 6)),
                                                                  'M251Q vs. WildType at 150 minutes in 22 degrees' = c(rep(0, 12), 0, -1, 0, 1, rep(0, 2), rep(0, 6)),
                                                                  'T244Q vs. WildType at 150 minutes in 22 degrees' = c(rep(0, 12), 0, -1, 0, 0, 1, 0, rep(0, 6)),
                                                                  'T244Q.M251Q vs. WildType at 150 minutes in 22 degrees' = c(rep(0, 12), 0, -1, 0, 0, 0, 1, rep(0, 6)),
                                                                  'K254Q vs. Uninfected at 150 minutes in 22 degrees' = c(rep(0, 12), -1, 0, 1, rep(0, 3), rep(0, 6)),
                                                                  'M251Q vs. Uninfected at 150 minutes in 22 degrees' = c(rep(0, 12), -1, 0, 0, 1, rep(0, 2), rep(0, 6)),
                                                                  'T244Q vs. Uninfected at 150 minutes in 22 degrees' = c(rep(0, 12), -1, 0, 0, 0, 1, 0, rep(0, 6)),
                                                                  'T244Q.M251Q vs. Uninfected at 150 minutes in 22 degrees' = c(rep(0, 12), -1, 0, 0, 0, 0, 1, rep(0, 6))))) %>%
  mutate(lower.95CL = estimate - SE*qt(0.975, df = df, lower.tail = TRUE),
         upper.95CL = estimate + SE*qt(0.975, df = df, lower.tail = TRUE),
         CI = paste0("(", round(lower.95CL, 2), ", ", round(upper.95CL, 2), ")")) %>%
  dplyr::select(-lower.95CL, -upper.95CL)
  colnames(mdl1.corexp.varexp.contrasts.of.interest) <- c("Contrast", "Estimated Difference", "SE", "df", "T", "P-Value", "95\\% CI")

  print(xtable(mdl1.corexp.varexp.contrasts.of.interest, digits = c(2, 2, 2, 2, 0, 2, 2, 2), align = "cccccccc"),
        include.rownames = F,
        sanitize.colnames = function (x) {x})


# Code Chunk:  lsmeans_mdl1_basicrand

# Get estimated values a few time points.  Plot those estimated curves over the data.
# Print out 95% confidence intervals at each point.
mdl1.basicrand.lsmeans <- lsmeans(mdl1.basicrand, ~ condition*degrees*minutes_cent,
                                  at = list(minutes_cent = c(unique(dta$minutes_cent))))
mdl1.basicrand.lsmeans.df <- summary(mdl1.basicrand.lsmeans) %>%
  filter((degrees == "22 degrees" & minutes_cent %in% unique(dta[which(dta$degrees == "22 degrees") , 'minutes_cent'])) |
           (degrees == "37 degrees" & minutes_cent %in% unique(dta[which(dta$degrees == "37 degrees") , 'minutes_cent']))) %>%
  mutate(lower.CL.44df = lsmean - qt(0.975, df = 44, lower.tail = T)*SE,
         upper.CL.44df = lsmean + qt(0.975, df = 44, lower.tail = T)*SE)

# Plot
ggplot(mdl1.basicrand.lsmeans.df,
       aes(x = minutes_cent + mean(dta$minutes), y = lsmean, fill = condition)) +
  facet_grid(. ~ degrees, scales = "free") +
  geom_ribbon(aes(ymin = lower.CL.44df, ymax = upper.CL.44df), alpha = 0.1) +
  geom_line(aes(colour = condition)) +
  theme(legend.position = 'bottom') +
  labs(x = "Minutes",
       y = "Model-Estimated Means",
       title = "Model-Estimated Means and Pointwise 95% Confidence Intervals") +
  theme(plot.title = element_text(hjust = 0.5))
```

```
# Test difference of wildtype and mutants at time = 25 minutes
mdl1.basicrand.lsmeans.for.contrasts <- lsmeans(mdl1.basicrand, ~ condition*degrees*minutes_cent,
                                               at = list(minutes_cent = c(25 - mean(dta$minutes),
                                                                          150 - mean(dta$minutes))))

mdl1.basicrand.contrasts.of.interest <- summary(contrast(mdl1.basicrand.lsmeans.for.contrasts,
                                                              list('K254Q vs. WildType at 25 minutes in 37 degrees' = c(rep(0, 6), 0, -1, 1, rep(0, 3), rep(0, 12)),
                                                                   'M251Q vs. WildType at 25 minutes in 37 degrees' = c(rep(0, 6), 0, -1, 0, 1, rep(0, 2), rep(0, 12)),
                                                                   'T244Q vs. WildType at 25 minutes in 37 degrees' = c(rep(0, 6), 0, -1, 0, 0, 1, 0, rep(0, 12)),
                                                                   'T244Q.M251Q vs. WildType at 25 minutes in 37 degrees' = c(rep(0, 6), 0, -1, 0, 0, 0, 1, rep(0, 12)),
                                                                   'K254Q vs. Uninfected at 25 minutes in 37 degrees' = c(rep(0, 6), -1, 0, 1, rep(0, 3), rep(0, 12)),
                                                                   'M251Q vs. Uninfected at 25 minutes in 37 degrees' = c(rep(0, 6), -1, 0, 0, 1, rep(0, 2), rep(0, 12)),
                                                                   'T244Q vs. Uninfected at 25 minutes in 37 degrees' = c(rep(0, 6), -1, 0, 0, 0, 1, 0, rep(0, 12)),
                                                                   'T244Q.M251Q vs. Uninfected at 25 minutes in 37 degrees' = c(rep(0, 6), -1, 0, 0, 0, 0, 1, rep(0, 12)),
                                                                   'K254Q vs. WildType at 150 minutes in 22 degrees' = c(rep(0, 12), 0, -1, 1, rep(0, 3), rep(0, 6)),
                                                                   'M251Q vs. WildType at 150 minutes in 22 degrees' = c(rep(0, 12), 0, -1, 0, 1, rep(0, 2), rep(0, 6)),
                                                                   'T244Q vs. WildType at 150 minutes in 22 degrees' = c(rep(0, 12), 0, -1, 0, 0, 1, 0, rep(0, 6)),
                                                                   'T244Q.M251Q vs. WildType at 150 minutes in 22 degrees' = c(rep(0, 12), 0, -1, 0, 0, 0, 1, rep(0, 6)),
                                                                   'K254Q vs. Uninfected at 150 minutes in 22 degrees' = c(rep(0, 12), -1, 0, 1, rep(0, 3), rep(0, 6)),
                                                                   'M251Q vs. Uninfected at 150 minutes in 22 degrees' = c(rep(0, 12), -1, 0, 0, 1, rep(0, 2), rep(0, 6)),
                                                                   'T244Q vs. Uninfected at 150 minutes in 22 degrees' = c(rep(0, 12), -1, 0, 0, 0, 1, 0, rep(0, 6)),
                                                                   'T244Q.M251Q vs. Uninfected at 150 minutes in 22 degrees' = c(rep(0, 12), -1, 0, 0, 0, 0, 1, rep(0, 6))))) %>%
  mutate(lower.95CL = estimate - SE*qt(0.975, df = df, lower.tail = TRUE),
         upper.95CL = estimate + SE*qt(0.975, df = df, lower.tail = TRUE),
         CI = paste0("(", round(lower.95CL, 2), ", ", round(upper.95CL, 2), ")")) %>%
  dplyr::select(-lower.95CL, -upper.95CL)
  colnames(mdl1.basicrand.contrasts.of.interest) <- c("Contrast", "Estimated Difference", "SE", "df", "T", "P-Value", "95\\% CI")

  print(xtable(mdl1.basicrand.contrasts.of.interest, digits = c(2, 2, 2, 2, 0, 2, 2, 2), align = "cccccccc"),
        include.rownames = F,
        sanitize.colnames = function (x) {x})

# Code Chunk: session_info
sessionInfo()
# Code Chunk: citations

  packages <- gsub(pattern = "package:",
                   replacement = "",
                   x = grep(pattern = "package", x = search(), value = TRUE))
  for(p in 1:length(packages)){
    cat("\n======================================================================",
        "\npackage:",packages[p],
        "\n----------------------------------------------------------------------\n")
    print(citation(package=packages[p]))
  }

# Code Chunk: model_summary_mdl1_corexp_varexp

summary(mdl1.corexp.varexp)

# Code Chunk: model_summary_mdl1_basicrand

summary(mdl1.basicrand)

# Code Chunk: model_summary_mdl1

summary(mdl1)

# Code Chunk: model_summary_mdl1_corexp

summary(mdl1.corexp)

# Code Chunk: model_diagnostic_plots_mdl1_corexp_varexp

par(mfrow = c(2,3))
plot(residuals(mdl1.corexp.varexp, type = "normalized") ~ fitted(mdl1.corexp.varexp),
     xlab = "Fitted Values", ylab = "Normalized Residuals")
abline(h = 0)
plot(residuals(mdl1.corexp.varexp, type = "normalized") ~ dta$minutes,
     xlab = "Minutes", ylab = "Normalized Residuals")
abline(h = 0)
plot(residuals(mdl1.corexp.varexp, type = "normalized") ~ dta$condition,
     xlab = "Virus Condition", ylab = "Normalized Residuals")
abline(h = 0)
plot(residuals(mdl1.corexp.varexp, type = "normalized") ~ factor(dta$degrees),
     xlab = "Degrees", ylab = "Normalized Residuals")
```

```r
abline(h = 0)
qqnorm(residuals(mdl1.corexp.varexp, type = "normalized"))
qqline(residuals(mdl1.corexp.varexp, type = "normalized"))
par(mfrow = c(1,1))
plot(acf.mdl1.corexp.varexp)



# Code Chunk: model_diagnostic_plots_mdl1_basicrand

par(mfrow = c(2,3))
plot(residuals(mdl1.basicrand, type = "normalized") ~ fitted(mdl1.basicrand),
     xlab = "Fitted Values", ylab = "Normalized Residuals")
abline(h = 0)
plot(residuals(mdl1.basicrand, type = "normalized") ~ dta$minutes,
     xlab = "Minutes", ylab = "Normalized Residuals")
abline(h = 0)
plot(residuals(mdl1.basicrand, type = "normalized") ~ dta$condition,
     xlab = "Virus Condition", ylab = "Normalized Residuals")
abline(h = 0)
plot(residuals(mdl1.basicrand, type = "normalized") ~ factor(dta$degrees),
     xlab = "Degrees", ylab = "Normalized Residuals")
abline(h = 0)
qqnorm(residuals(mdl1.basicrand, type = "normalized"))
qqline(residuals(mdl1.basicrand, type = "normalized"))
par(mfrow = c(1,1))
plot(acf.mdl1.basicrand)

# Code Chunk: model_diagnostic_plots_mdl1

par(mfrow = c(2,3))
plot(residuals(mdl1, type = "normalized") ~ fitted(mdl1),
     xlab = "Fitted Values", ylab = "Normalized Residuals")
abline(h = 0)
plot(residuals(mdl1, type = "normalized") ~ dta$minutes,
     xlab = "Minutes", ylab = "Normalized Residuals")
abline(h = 0)
plot(residuals(mdl1, type = "normalized") ~ dta$condition,
     xlab = "Virus Condition", ylab = "Normalized Residuals")
abline(h = 0)
plot(residuals(mdl1, type = "normalized") ~ factor(dta$degrees),
     xlab = "Degrees", ylab = "Normalized Residuals")
abline(h = 0)
qqnorm(residuals(mdl1, type = "normalized"))
qqline(residuals(mdl1, type = "normalized"))
par(mfrow = c(1,1))
plot(acf.mdl1)
# plot(Variogram(mdl1, form = ~ minutes))


# Code Chunk: model_diagnostic_plots_mdl1_corexp

par(mfrow = c(2,3))
plot(residuals(mdl1.corexp, type = "normalized") ~ fitted(mdl1.corexp),
     xlab = "Fitted Values", ylab = "Normalized Residuals")
abline(h = 0)
plot(residuals(mdl1.corexp, type = "normalized") ~ dta$minutes,
     xlab = "Minutes", ylab = "Normalized Residuals")
abline(h = 0)
plot(residuals(mdl1.corexp, type = "normalized") ~ dta$condition,
     xlab = "Virus Condition", ylab = "Normalized Residuals")
abline(h = 0)
plot(residuals(mdl1.corexp, type = "normalized") ~ factor(dta$degrees),
     xlab = "Degrees", ylab = "Normalized Residuals")
abline(h = 0)
qqnorm(residuals(mdl1.corexp, type = "normalized"))
qqline(residuals(mdl1.corexp, type = "normalized"))
par(mfrow = c(1,1))
plot(acf.mdl1.corexp)

mychunks <- knitr::all_labels()
```
